# Prediction of aluminium distributions in zeolites using basin-hopping and machine learning potentials

**DOI:** 10.1039/d6dd00356g

**Published:** 2026-10-30

**Authors:** Oscar Bengtsson, Daniel Hewitt, Indranil Saha, Misbah Sarwar, Nicola Collis, Andreas Erlebach, Lukáš Grajciar, Christopher J. Heard, Ben Slater

**Affiliations:** a Department of Chemistry, University College London 20 Gordon Street London WC1H 0AJ UK oscar.bengtsson.21@ucl.ac.uk B.Slater@ucl.ac.uk; b Department of Physical and Macromolecular Chemistry, Faculty of Science, Charles University in Prague 12483 Czech Republic; c Johnson Matthey Technology Centre Sonning Common, Blounts Ct Rd, Sonning Common, Reading RG4 9NH UK

## Abstract

In this work, we present a systematic approach to predicting the structures of three sodiated aluminosilicate zeolites (Chabazite, Mordenite and RHO) by combining an accurate neural network potential with a custom basin-hopping algorithm. We show that the low energy, thermally accessible configurations are rare in comparison to the number of possible structures, accounting for a few percent of viable structures. For Chabazite and Mordenite, it is found that there is a tendency for aluminium to preferentially aggregate with 2 or 3 silicon atoms separating aluminium atoms with a strong disincentive to form Al–O–Si–O–Al linkages. However, clear and contrary topological preferences are observed in RHO where Al–O–Si–O–Al linkages are strongly favoured at moderate Al concentrations. Crucially, randomly generated or non-basin hopping approaches to structure generation results in configurations that are highly metastable and activated with respect to minimum energy structures. Using such activated structures for modelling catalysis processes could potentially yield qualitatively incorrect predictions of reactivity and transport properties.

## Abstract 2

We present a new method to predict the structures of aluminosilicate zeolites using an accurate neural network potential and a custom basin-hopping method and demonstrate it's utility on Chabazite, Mordenite and zeolite RHO.

## Graphical abstract

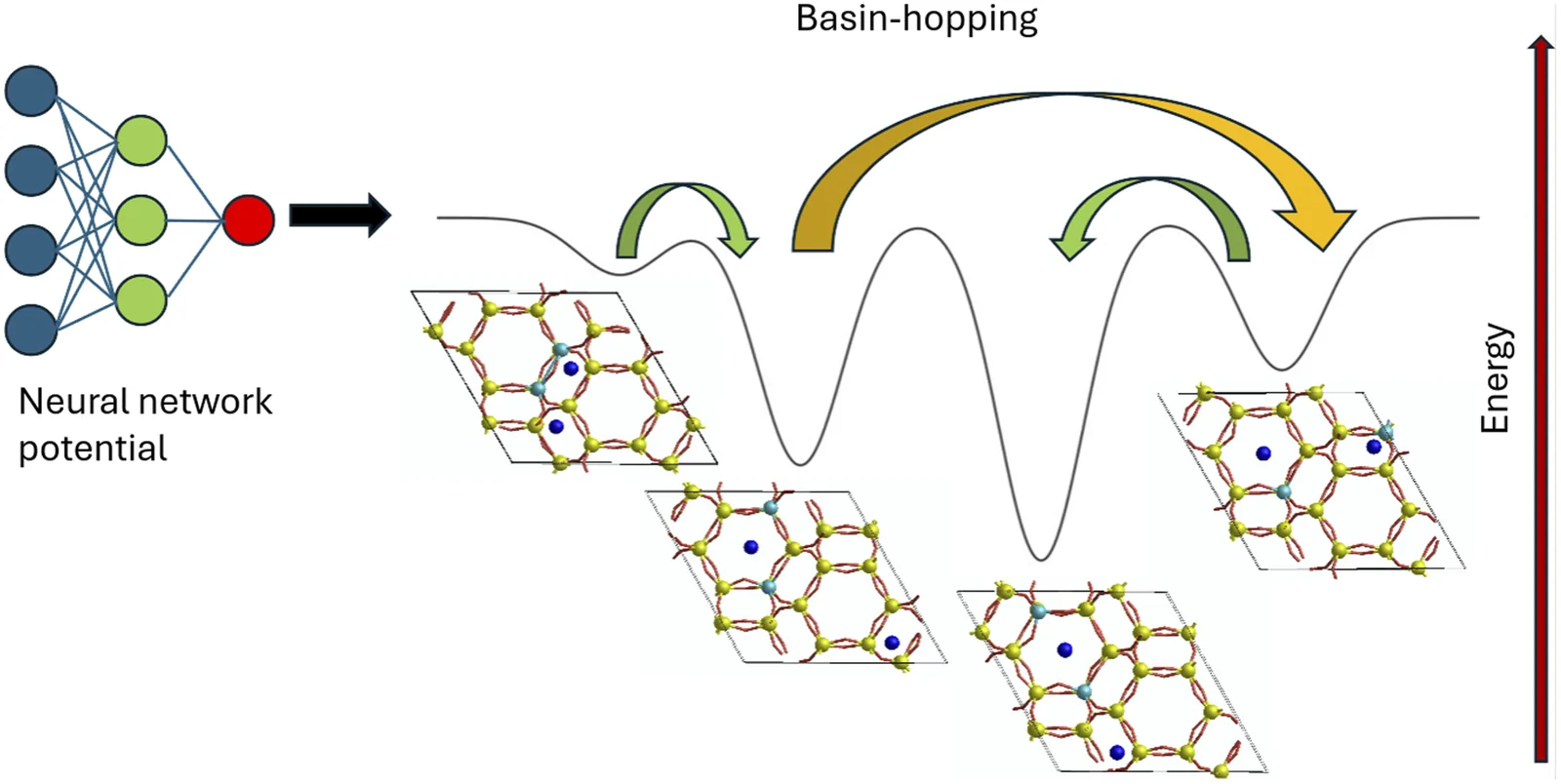

## 1 Introduction

Zeolites, framework aluminosilicates, are unsung heroes of catalysis, employed for petrochemical cracking, molecular sieving and numerous critical catalytic chemical transformations at multi-million tonnage levels per annum.^1,2^ Despite their unrivaled commercial importance for over 70 years, there is a very basic and longstanding problem with zeolites; the location of the acid or active sites in these materials is generally unknown.^3^ Hence, structure/property relationships cannot generally be extricated from the array of factors that can influence their synthesis. If the structures of these materials could be evaluated accurately, this could open up the possibility of systematically screening^4,5^ and optimising catalysts, potentially yielding a dramatic reduction in energy and associated costs.^6^

In this work, we present a novel approach to determining the aluminum distribution in zeolites by integrating a neural network potential (NNP), developed by Erlebach *et al.*,^7^ with a custom basin-hopping algorithm to identify low-energy configurations. This allows for high-throughput evaluation of millions of configurations while maintaining consistency with the underlying quantum chemical energetics. The model effectively captures both anhydrous and hydrated systems with either sodium or protons as charge-compensating ions.^7–9^ Here, we focus exclusively on sodium-compensated zeolites, as sodium is most commonly used as a structure-directing agent (SDA) during synthesis before being exchanged for other ions, such as protons.

The key problem in evaluating aluminosilicate zeolite structures is a combinatorial one; the number of possible configurations is vast even for relatively simple and single unit cells which prohibits direct evaluation by DFT. The addition of each aluminium atom factorially increases the number of distributions, and all possible permutations of the charge compensating ion position must be considered, which itself scales exponentially with the number of aluminium sites. This combinatorial challenge is particularly pronounced in low-symmetry zeolites and larger unit cells, where the solution space becomes intractable beyond a few aluminium atoms and their charge-compensating ions. Since each potential structure must be geometry optimised, requiring hundreds of energy evaluations, exhaustive evaluation by DFT is infeasible. However, using the NNP and exploiting methods from global optimisation can shed light on the pool of thermodynamically relevant structures. We now describe how we have generated structures, validated the accuracy of the potentials and show the adapted basin-hopping approach for zeolites; this enables to predict the structure and properties of the small subset of relatively stable aluminosilicates from the bewildering array of structural possibilities.

## 2 Zeolite structures and structure generation

Zeolites are a form of tectosilicate material and there are 264 reported distinct zeolite structures at the time of writing^10^ that differ in terms of density and accessible porosity; these microporous structures can contain cages, and/or networks of channels that facilitate catalysis. The structures are comprised of TO_4_ tetrahdra (T = Si, Al, Ge *etc*, O is oxygen) that are connected at their edges to form the topology of the framework,^11,12^ exemplified in Fig. 1 which shows Chabazite (CHA), a zeolite which is commonly used for methanol to olefin catalysis and selective catalytic reduction of nitrogen oxide. As the aluminium 3+ ion is negative with respect to silicon 4+, it requires a charge compensating counter-cation such as sodium, lithium, copper or a proton to achieve charge neutrality. The counter-cation affects the aluminium distribution due to differences in the size and effective charge of cations.^13^

**Fig. 1:**
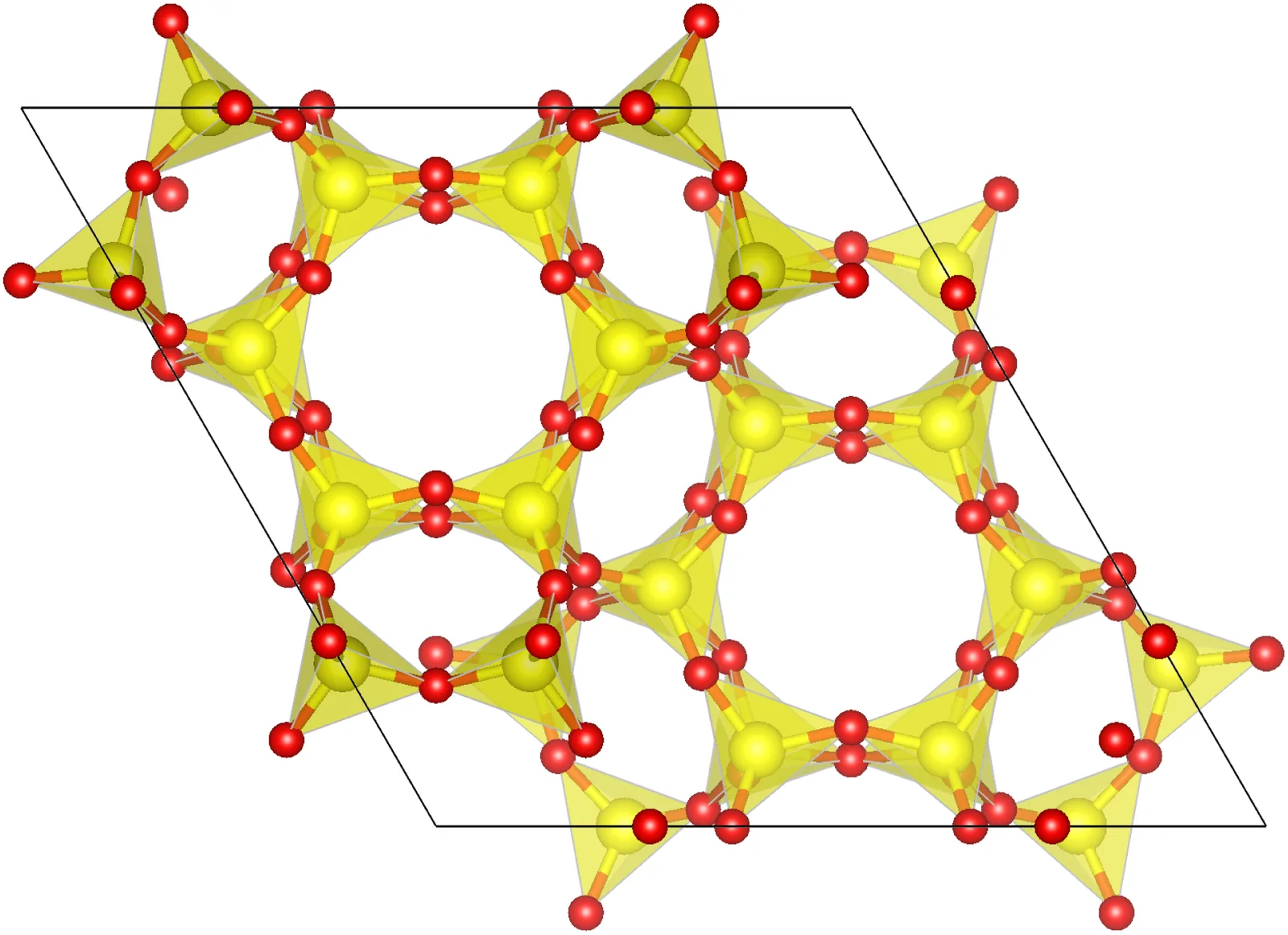
SiO_4_ tetrahedra defining the chabazite topology (CHA) where the silicon is shown in yellow and oxygen atoms are shown in red. The cell is viewed along the [001] axis.

It is generally accepted that so-called non-Löwenstein (NL) structures with Al–O–Al linkages are energetically unfavourable due to large electrostatic repulsion between aluminium atoms.^14^ However, as Fletcher *et al.*^15–17^ observed, proton-compensated frameworks tend to prefer these (NL) configurations. But since zeolites are primarily synthesised with metal ions (most commonly sodium) as the charge-compensating species before being exchanged for a proton, as-synthesised protonated zeolites are essentially kinetically trapped and metastable. Another factor is the effect of hydration as zeolites are most typically synthesised in water; Heard *et al.*^18^ have shown water content influences the preferred distribution for proton compensated frameworks, where beyond a certain degree of hydration NL frameworks are no longer favourable. Within this work, we focus entirely on anhydrous sodium-compensated frameworks. These structures are relevant to catalysis; post-synthesis, the zeolite is calcined to remove water, any SDA and encourage liberation of extra-framework species. The aluminium location is thus determined in part by the sodium, fixing the aluminium prior to exchange with protons to create Brønsted acid sites that can be active sites.

There are many examples of previous work to model the location of aluminium and counterions in the literature but we believe this is the first attempt to establish global minima structures in a large range of Si : Al ratios in an unbiased way. Crucially, we account for the thermodynamic reorganisation of the aluminium and counterion distribution including relaxation effects. Jeffroy *et al.*^19^ reported a Monte Carlo approach that allowed for Al and counterion reorganisation but excluding relaxation effects that are critically important in identifying the most stable structures (see SI and for example, Ruiz-Salvador *et al.*^20^). Muraoka *et al.*^21^ generated and optimised zeolite aluminosilicate structures for a range of topologies, however, just 40 distributions per Si : Al ratio, per topology were considered compared to millions in this work. Ruiz-Salvador and co-workers have published extensively in this area, for example ref. 20 where symmetry and spatial occupation constraints were used in the generation of cells – this differs critically from our approach that captures spatial inhomogeneity that we find is an important feature at particular Si : Al ratios (see 5.3). More recent work by Abatal *et al.*^22^ has used DFT and simulated annealing approaches to explore cation positioning but with a fixed distribution of aluminium in contrast to this work, where the position of both the aluminium and the counterion changes to optimise thermal stability of the structure. In summary, we believe our approach is the most comprehensive and unbiased way of assessing the true structures of aluminosilicate zeolites reported to date.

### 2.1 Sodium positioning

From chemical intuition, the sodium ion is expected to be located near a given aluminium atom, as this arrangement maximises the charge compensation. Several algorithms were developed in this work to determine the optimal sodium position. One of these we named the sum-vector-projection (SVP) algorithm (eqn (1)), which generates four possible sodium positions for each aluminium atom. Another method developed was the bond-vector-projection (BVP) or “edges algorithm,” which places a sodium atom at each of the six edges of the TO_4_ tetrahedron (see Fig. 2). The SVP approach considers all oxygen atoms bonded to a given aluminium atom and generates the possible sodium atoms by considering a vector projection *P⃑* from each oxygen^9^1
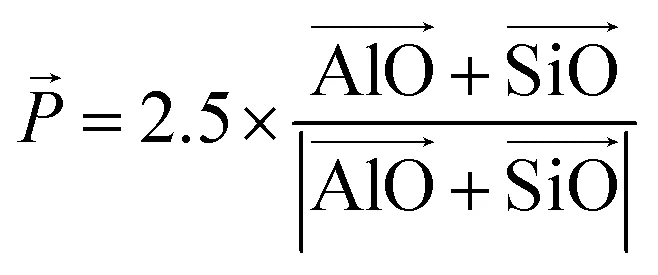

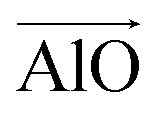
 and 
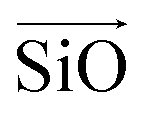
 correspond to cartesian vectors from the aluminium to the oxygen and silicon to the oxygen respectively, the vector projection is then normalised to unit length and scaled by a factor of 2.5 and added to the oxygen's position thereby placing the sodium 2.5 Å from the oxygen. The BVP algorithm (eqn (2)) was motivated by the observation that the true minimum of sodium in zeolite RHO (see the SI Section 1 and 1.1) was located along an “edge” of the AlO_4_ tetrahedron, which was not sampled within the SVP algorithm. In this approach, sodium positions were generated by identifying the midpoint of each tetrahedral edge and then placing the sodium 2 Å from the Al on a vector which passes through this midpoint, resulting in 6 possible positions (as shown in Fig. 2). If 
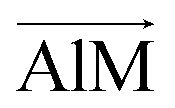
 is the vector joining the aluminium Al with the midpoint *M* of an edge, then the position of the sodium is given as2
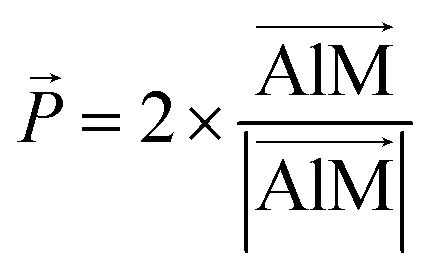


**Fig. 2:**
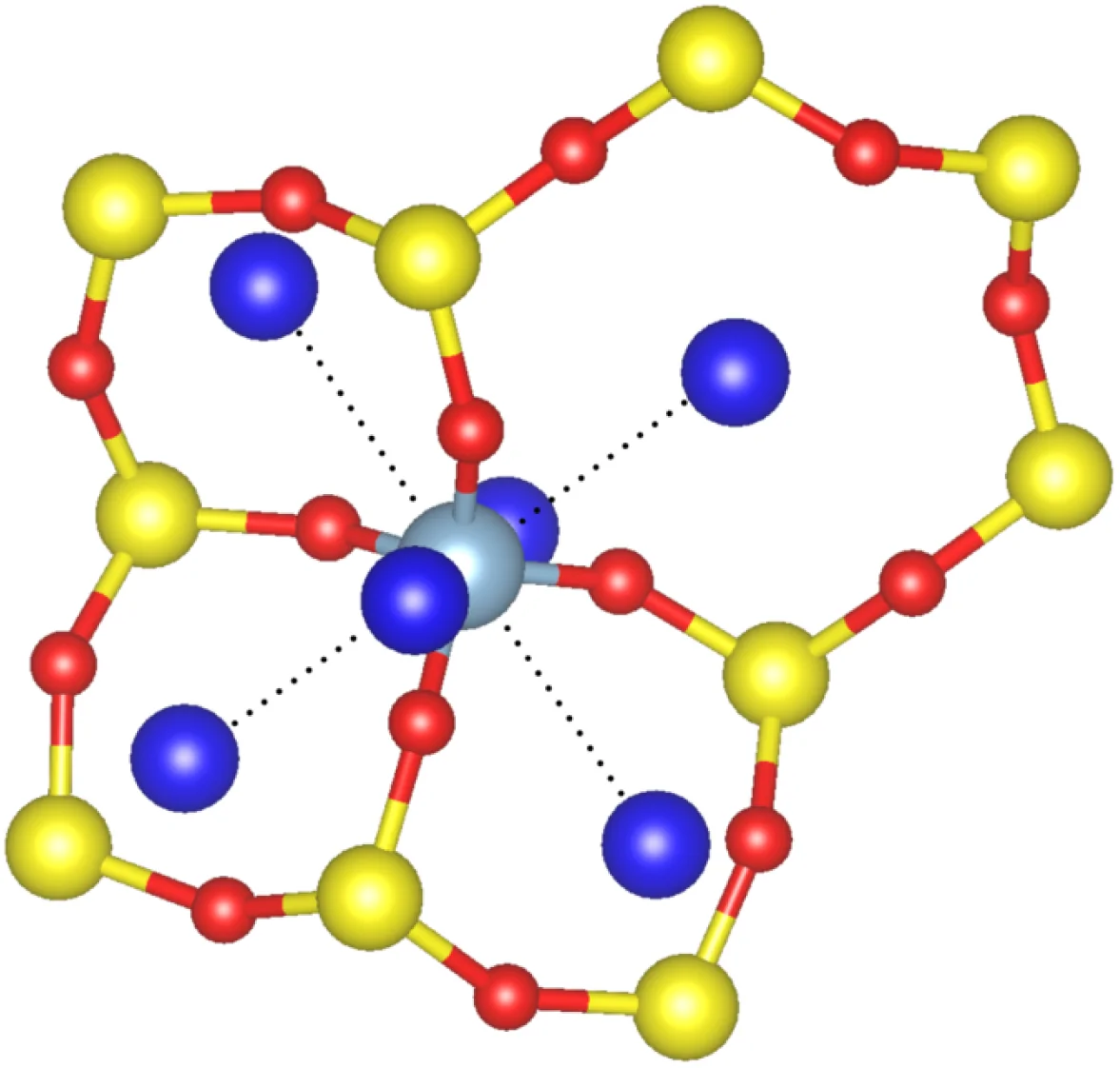
The six positions for Na predicted according to the BVP approach, where the dotted lines are guides to the eye, to show the octahedrally distributed locations. Aluminium is shown in cyan, oxygen in red and silicon in yellow.

In the custom basin-hopping approach described here, all topologies used the BVP approach to ensure a more comprehensive sampling of cation positions.

## 3 Benchmarking the NNP

In order to assess the veracity of the NNP compared to quantum chemical methods, the model was benchmarked against two levels of DFT (GGA and hybrid), with a wide range of configurations. Periodic DFT calculations were carried out using CP2K,^23^ a Gaussian and plane-wave based DFT code. A selection of NNP optimised configurations were re-relaxed using DFT to see how the relative energy rankings were affected for chabazite as a model material. We used different Si : Al ratios and different cell sizes to gauge how well the potential performs within different systems. Although the NNP was originally fitted to SCAN+D3 data, we measured the performance of the NNP against PBE+D3 and PBE0+D3 (ref. 24) as SCAN is notoriously sensitive to geometry and computational settings.^25^ We also note that the NNP used here has been extensively benchmarked against a range of sodiated aluminosilicate phases and water in the report of Erlebach *et al.*^7,9^ with a mean absolute error of 5.3 meV per atom (≈0.51 kJ mol^−1^ per atom). The potential was assessed in different Si : Al ratios, in a 36 T-site 1 × 1 × 1 cell and a larger 72 T-site 1 × 2 × 1 supercell, the motivation for using the larger cell was to gauge the effects of longer-ranged relaxation. Using the exhaustive symmetry structure generation procedure described in Section 6.3 of the SI, all symmetry unique configurations for 2 aluminium atoms in 36 T-site CHA were generated and then fully optimised (cell parameters and atom positions) using the NNP. The 393 configurations were then re-relaxed using PBE+D3 and their relative energies plotted as shown in Fig. 3. The *R*^2^ for this data is 0.91 and MAE is 6.47 kJ mol^−1^ which is a good level of correlation with an error that is smaller than the difference in commonly utilised density functionals.^24,26^ The degree of correlation appears slightly worse for higher energy configurations than low energy structures, but since our target is the latter, the NNP is demonstrably robust.

**Fig. 3:**
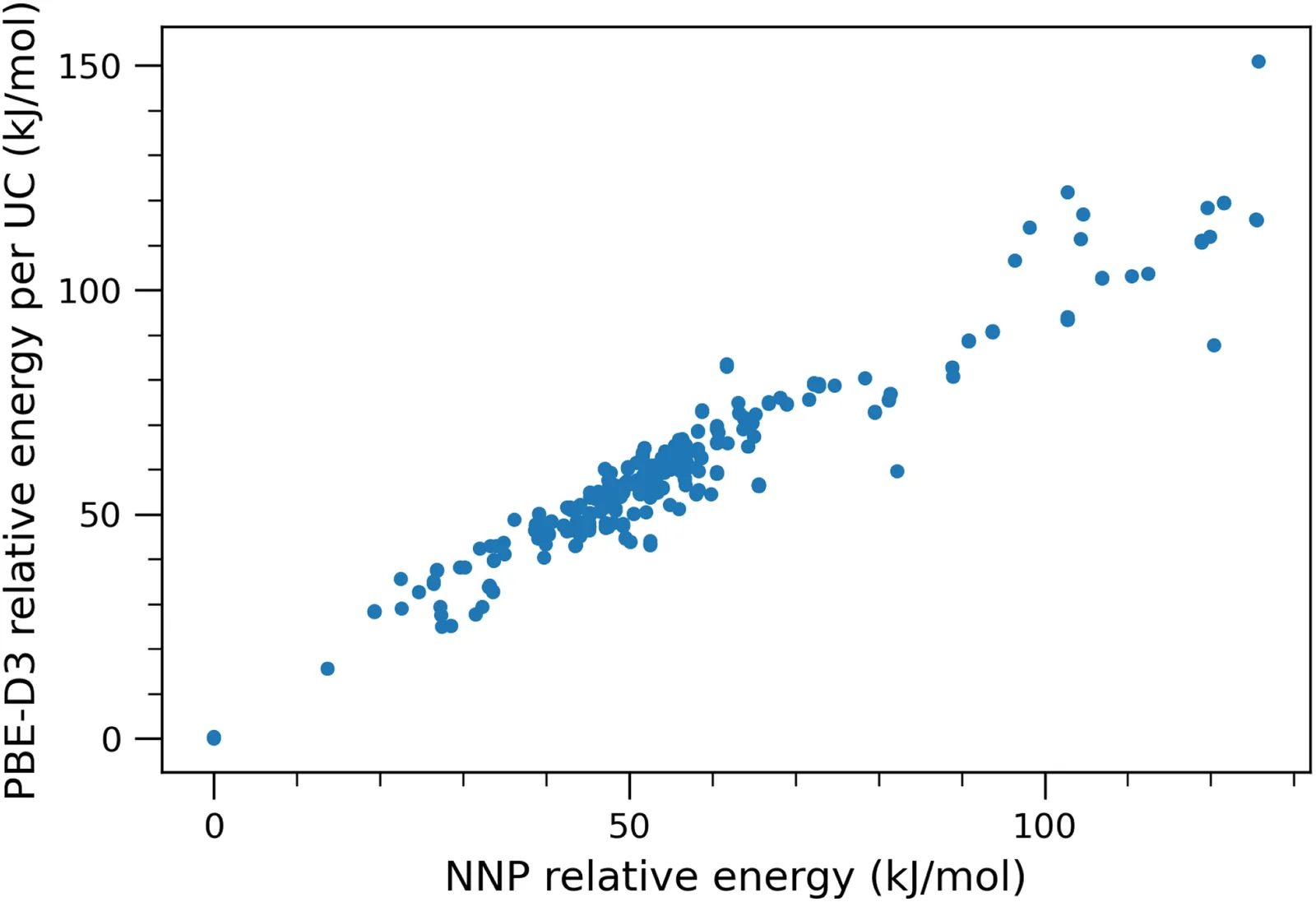
Relative energy of NNP *vs.* PBE+D3 for 393 different structures corresponding to 2 aluminium atoms in 36 T-site 1 × 1 × 1 CHA.

To compare other Si : Al ratios and long-ranged spatial relaxation effects, structures were generated using a stepwise configuration approach (as described in Section 6.5 of the SI) with the top 10 lowest energy configurations being re-relaxed under GGA PBE+D3 and hybrid functional PBE0+D3, the results of these calculations for the 36 and 72 T-site systems are shown in Tables 1 and 2 respectively. Note that the values reported for 2 aluminium atoms in the 36 T-site system correspond to the top 30 configurations (lowest energy) for both functionals. In Fig. 4, assuming the hybrid functional results have the lowest mean absolute error with respect to the true energy rankings, it seems the NNP (fitted to SCAN+D3) tends to underestimate the relative energy of configurations but the qualitative ranking is impressively consistent between all methods. Inspection of Tables 1 and 2 shows errors are remarkably small. Importantly, there is no evidence of the error increasing systematically with the number of aluminium and sodium atoms introduced. Hence, the potential performs well across varied concentrations of aluminium and different cell sizes, which instills confidence in predicting the most thermodynamically relevant configurations for different topologies with distinct compositions.

**Table 1:** Comparison of the mean absolute error (MAE) in relative energies of the 10 lowest energy configurations according to NNP relaxation with the same configurations re-optimised using PBE+D3 and PBE0+D3, in the 36 T-site CHA 1 × 1 × 1 cell

*N*_Al_	Si/Al ratio	PBE+D3 MAE (kJ mol^−1^)	PBE0+D3 MAE (kJ mol^−1^)
1	36.0	0.56	0.64
2	17.0	6.17	7.44
3	11.0	1.64	2.01
4	8.0	4.49	6.21

**Table 2:** Comparison of the mean absolute energy error for NNP relative energies compared to PBE+D3 and PBE0+D3 in 72 T-site 1 × 2 × 1 CHA

N_Al_	Si/Al ratio	PBE+D3 MAE (kJ mol^−1^)	PBE0+D3 MAE (kJ mol^−1^)
1	35.0	6.23	7.68
2	23.0	7.53	1.11
3	17.0	5.88	7.80
4	13.0	1.37	1.27
5	11.0	7.67	13.55
6	9.3	1.69	1.20
7	8.0	4.20	7.66

**Fig. 4:**
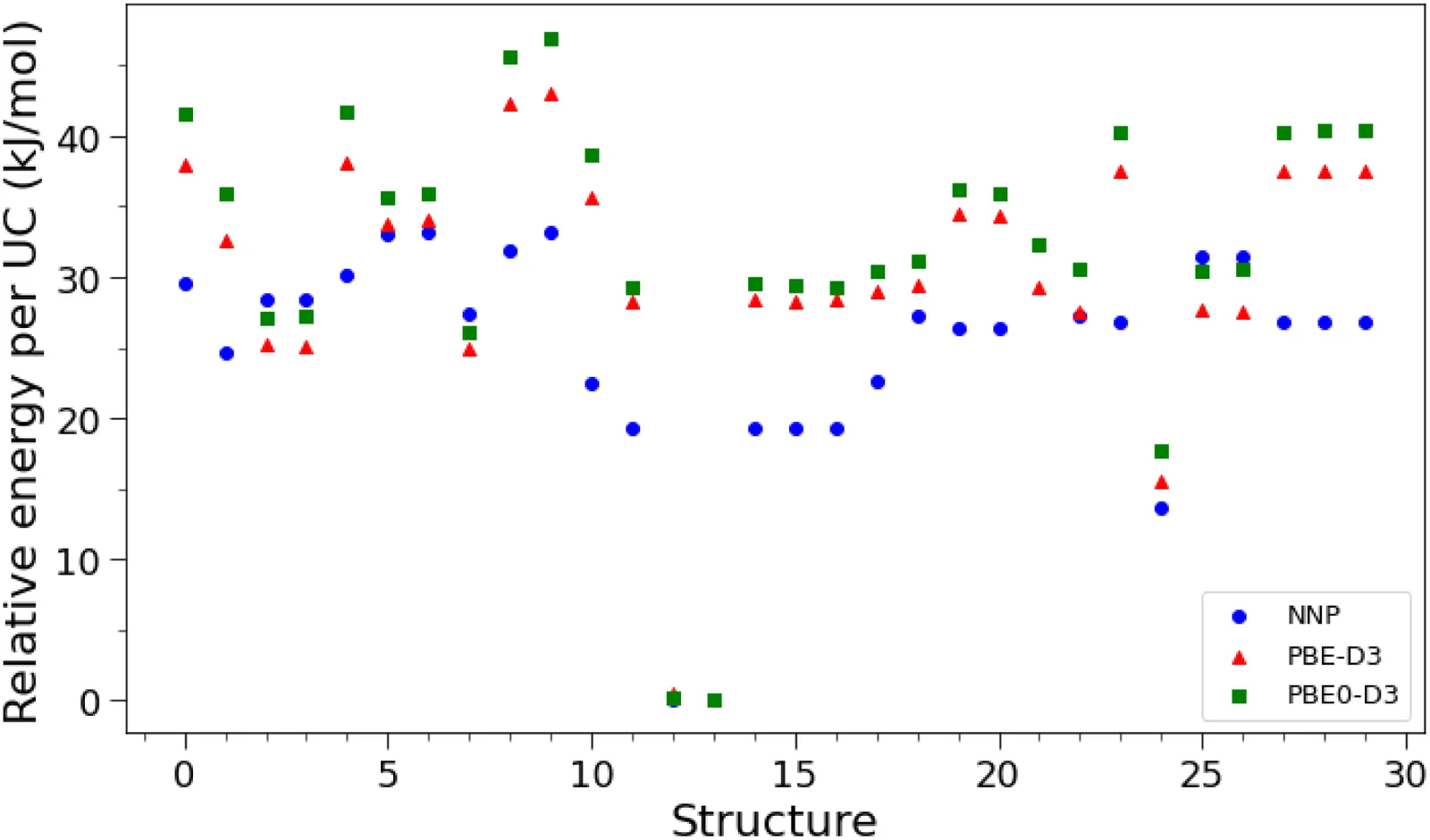
Relative energy of NNP *vs.* PBE+D3 *vs.* PBE0+D3 for 30 lowest energy structures corresponding to 2 aluminium atoms in 36 T-site 1 × 1 × 1 CHA.

To test how well the potential performs on other topologies, 20 structures in a 7 Al/Na 96 T-site 1 × 1 × 2 MOR system were generated. The structures corresponded to randomly generated configurations, meaning each Al was placed at a random T site, with the corresponding sodium placed at a randomly selected BVP position. These configurations were first relaxed with the NNP and then re-relaxed with PBE+D3, the relative NNP energies were well correlated with the corresponding PBE+D3 energies with an *R*^2^ of 0.99, indicating the potential is reliable across other topologies.

## 4 Methodology

Zeolite structure modelling using atomistic approaches has a long history.^21,27–35^ Forcefield approaches have been able to accurately rank purely siliceous zeolites for three decades.^30^ Systematic modelling of aluminosilicate zeolite structures is less well represented in the literature although there have been comprehensive studies probing the effects of silicon/aluminium ratio on stability^21^ and there has been extensive, albeit random, sampling of specific and technologically important structures like ZSM-5.^36^ Moreover, Monte Carlo and simulated-annealing-based approaches have been used to gain insight into systems like mordenite.^19,37,38^ Here, we describe a general approach to predicting the aluminosilcate zeolite structures where we use brute force (BF) exhaustive sampling where practicable and present an efficient basin-hopping^39^ (BH) approach when the configurational space is prohibitively large for exhaustive sampling. As a comparator to BH, we use the “stepwise” method, as previously adopted in Fletcher *et al.*^15^ for much lower aluminium content. In the stepwise method, the global minimum position of aluminium and sodium for 1 aluminium and 1 sodium is used as the seed structure for generating all structures containing 2 aluminium atoms and 2 sodium atoms (with the BVP approach for sodium positions) and so on (see details in Section 6.5 of SI).

### 4.1 Basin hopping algorithm

Within this work we tailor the “basin-hopping” algorithm to the problem of sodium compensated zeolites. Basin-hopping is a stochastic global optimisation algorithm that refines candidate structures by applying random perturbations, called the “move-class”, followed by local minimisation to reduce the objective function (energy).^39^ Perturbations are then accepted or rejected based on the Metropolis criterion.^40^ This technique has already been used successfully to study germanium distributions in zeolites^41^ where brute force energy minimisation of all configurations is typically intractable. However, since there is no charge compensating ion, this problem has fewer degrees of freedom than that of aluminosilicate zeolites, applying basin-hopping to the latter therefore requires additional considerations as to what constitutes an appropriate move class.

Whilst similar in some ways to Monte Carlo, basin-hopping incorporates local optimisation into the global search, this is crucial since initial and optimised zeolites are generally poorly correlated^20^ and crucially, insufficiently correlated to be able to eliminate configurations from direct relaxation (see Section 5.1 in the SI for further discussion). Thus, Monte Carlo approaches which rely upon single-point energies may not be optimal.

#### 4.1.1 Move-class

The move-class for this algorithm involves swapping a randomly selected aluminium with a silicon atom. The associated sodium with the aluminium is then moved to a random position near the new aluminium site. The full move-class is detailed below:

Replace random Al with Si, remove Na associated to that Al (see Section 5.2 of the SI), replace random Si with Al, assign new Na position *via* BVP at new Al site (Fig. 5).

**Fig. 5:**
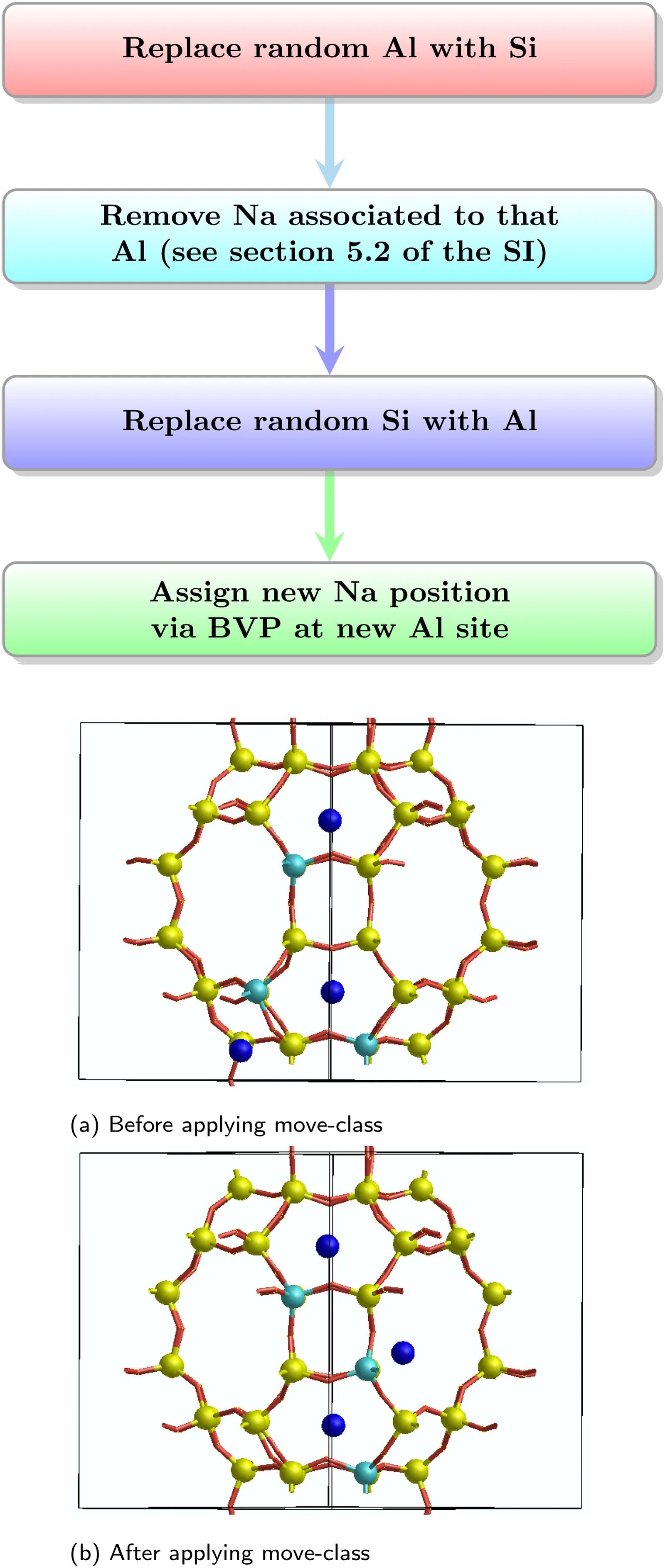
Move-class workflow and a comparison of 3 aluminium containing RHO structures before and after applying the basin-hopping move-class. Aluminium in cyan, silicon in yellow, sodium in blue and oxygen omitted for clarity.

The net effect of applying this move-class is that the positions of a randomly selected aluminium/sodium pair are updated, resulting in a perturbation to the structure. Additional checks are made to ensure the configuration is chemically reasonable and that all aluminium atoms in the structure are within 5 Å of a sodium atom. Once a valid move is selected, the structure is then optimised, and the move is accepted/rejected based on the standard Metropolis criterion. Within this approach the candidate configuration is optimised first at constant volume and then at constant pressure to allow for cell relaxation.

## 5 Results

We now present the outcome of extensive sampling of three distinct frameworks, CHA, MOR and RHO which differ in terms of structural complexity (symmetrically distinguishable T sites) and porosity (CHA and RHO are both essentially cage type zeolites while MOR is a channel type zeolite). We discuss qualitative features of the low energy structures as well as compare the respective landscapes for the different systems. Various properties of low energy configurations found by the basin-hopping algorithm are averaged using Boltzmann weighting across the ensemble. It is important to emphasise that basin-hopping is a global optimisation algorithm not a equilibrium thermal sampler, thus any Boltzmann weighted plots shown in the section (for example, Fig. 8) should be understood as averages over sampled local minima, not necessarily the true thermodynamic averages. In these averages an arbitrary energy window of 30 kJ mol^−1^ was chosen; discussion in Section 8 of the SI shows that the conclusions are not strongly dependent on this window. More on how the BH calculations were run, including discussion of convergence, is detailed in the SI.

### 5.1 CHA

The performance of the algorithm was tested on a 1 × 2 × 1 supercell with 72 T-sites. The algorithm was applied to 7 different Si : Al ratios corresponding to 2–8 aluminium atoms in the cell. It was then compared to the stepwise configuration approach for the same range of Si : Al ratios. BH typically matched or slightly outperformed the stepwise configuration approach, demonstrating that the algorithm is sampling relatively low-energy configurations (see Table S7 in SI for more details). Using the BH findings for CHA, it was found that the aluminium favors a NNN (next-next-nearest neighbour) arrangement as shown in Fig. 6. This is likely due to charge compensation effects where by this arrangement maximizes the degree to which each sodium can passivate the electronegative oxygen atoms bonded to aluminium atoms.

**Fig. 6:**
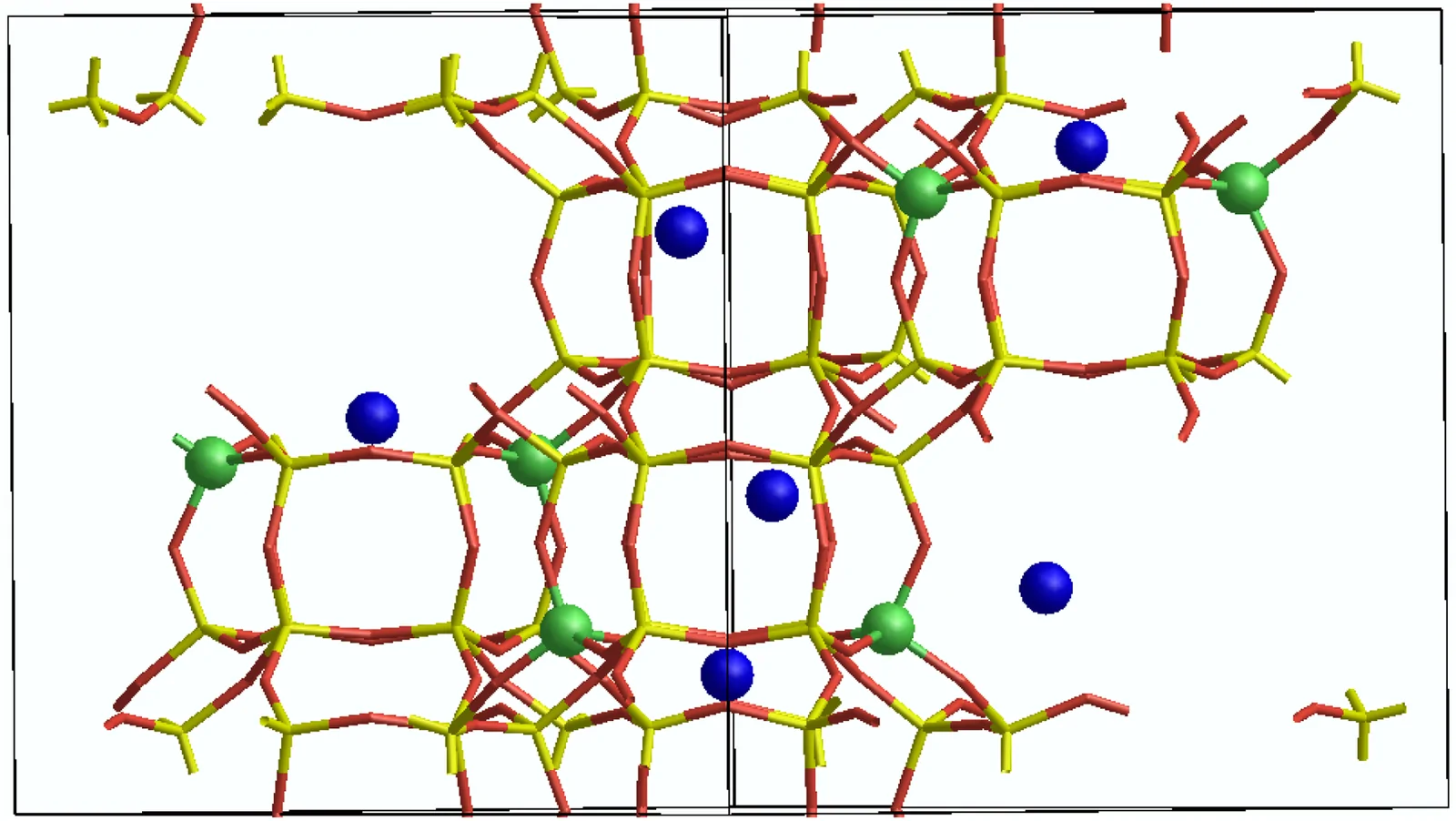
1 × 2 × 1 CHA global minimum for 6 aluminium atoms. Aluminium is in green and sodium is in blue; silicon and oxygen in yellow and red respectively.

Fig. 7 illustrates the full energy landscape for 2 aluminium atoms in a 1 × 2 × 1 cell, these configurations were generated using the exhaustive BF sampling method detailed Section 6.3 SI and optimised with the NNP. The figure shows that only a very small percentage of configurations are within 30 kJ mol^−1^ of the global minimum, thus selecting random configurations is highly unlikely to yield realistic structures that reflect the true structure of the catalyst. This finding holds for much lower Si : Al ratios; for example for 8 aluminium atoms in the 72 T-site cell it was found that for 1000 randomly generated configurations the mean energy across all structures was ≈270 kJ mol^−1^ above the global minimum predicted by basin-hopping. The lowest energy random configuration was ≈100 kJ mol^−1^ above the global minimum (GM), highly unstable compared to the preferred configuration.

**Fig. 7:**
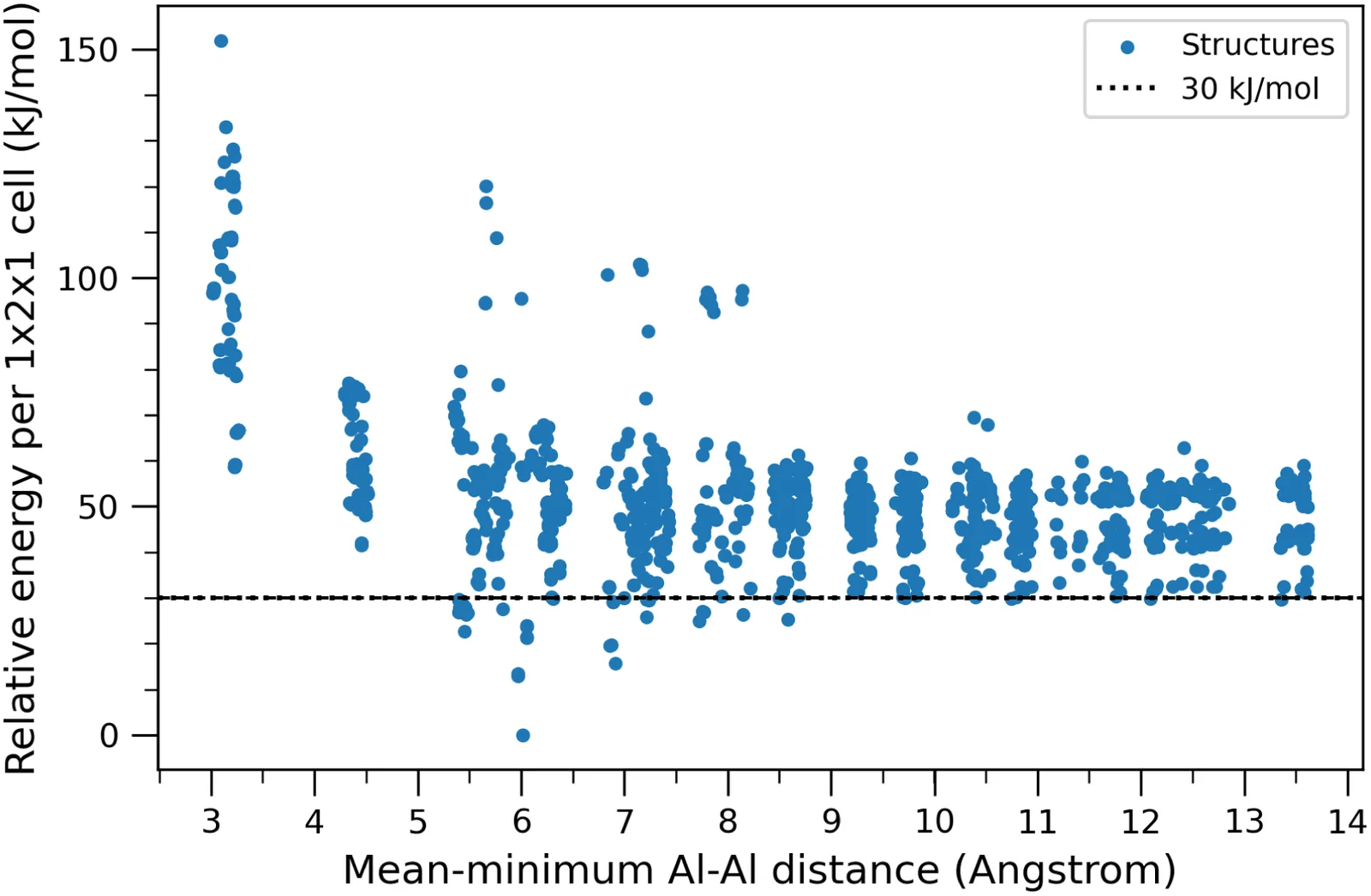
1 × 2 × 1 CHA optimised structures from the brute force approach with 2 aluminium atoms, 2049 configurations in total with 44 (2.15%) of configurations within an arbitrary 30 kJ mol^−1^ of the GM.

The plot would appear to contradict Dempsey's rule^42^ which states that aluminium tend to be maximally separated to minimise electrostatic repulsion, however the figure instead suggests that this becomes a negligible factor beyond around 8 Å, as previously found in Fletcher *et al.*^15^ The optimum energy structure occurs at around a mean Al–Al separation of approximately 6 Å, corresponding to a NNN (*i.e.* -Al-(O–Si–O)_2_–Al- arrangement. As seen in Fig. 8 and 9, showing a Boltzmann weighted average over Si separation counts for Al pairs in a single unit cell and 1 × 2 × 1 supercell, this preference for NNN separated aluminium is consistent across many Si : Al ratios and cell sizes. Although the concentrations of Al are unequal in these figures, it is very evident that the smaller cell restricts the maximum separation of Al for obvious geometric reasons and in the larger cell a much broader distribution of separations between Al pairs can be seen. Further evidence indicating the geometric constriction imposed by the single unit cell constraints are evidenced in S3, in the overpopulation analyses.

**Fig. 8:**
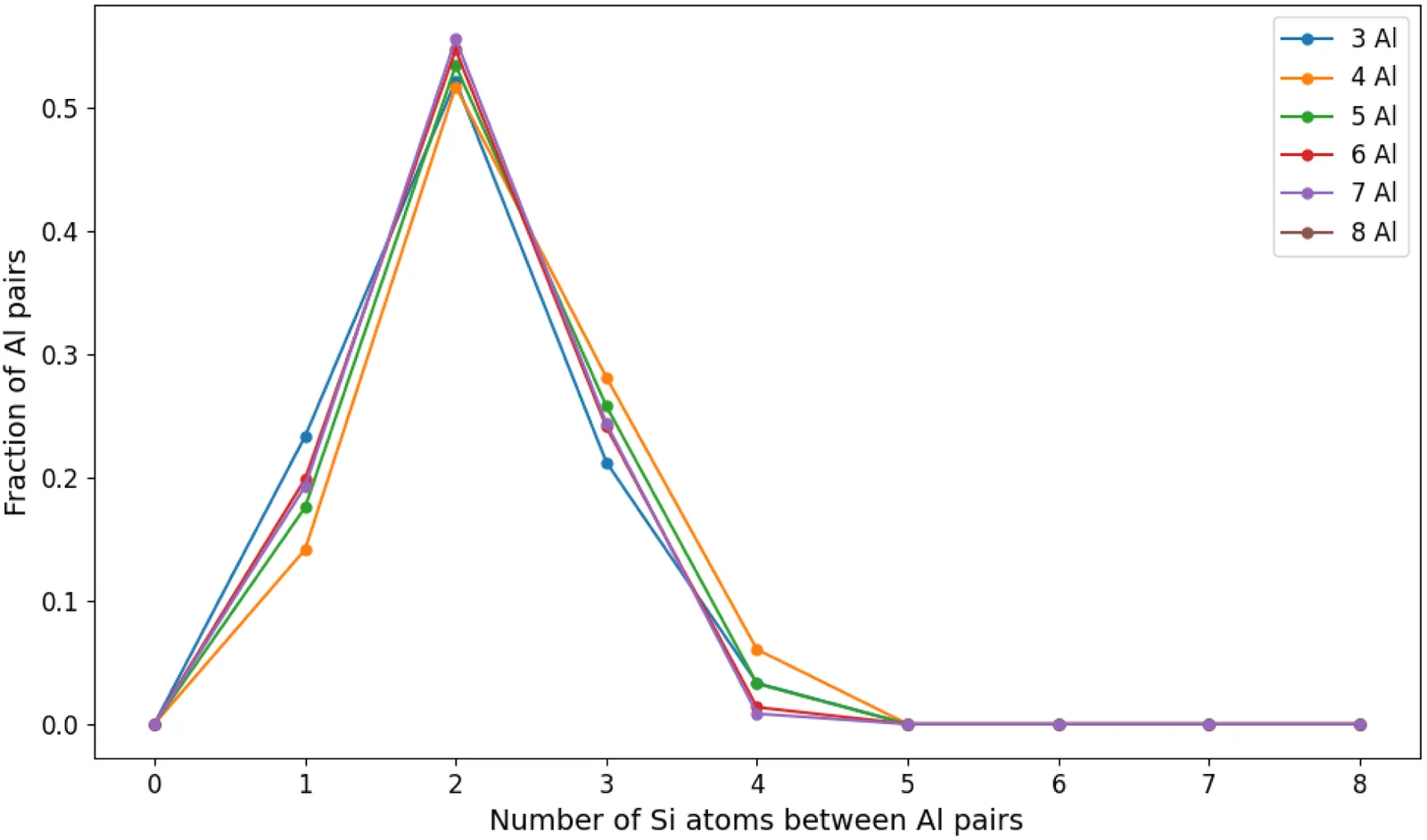
Number of Si atoms separating pairs of Al atoms in zeolite CHA (1 × 1 × 1), calculated over all structures within an arbitrary 30 kJ mol^−1^ of the global minimum, accounting for Boltzmann weighting at 450 K.

**Fig. 9:**
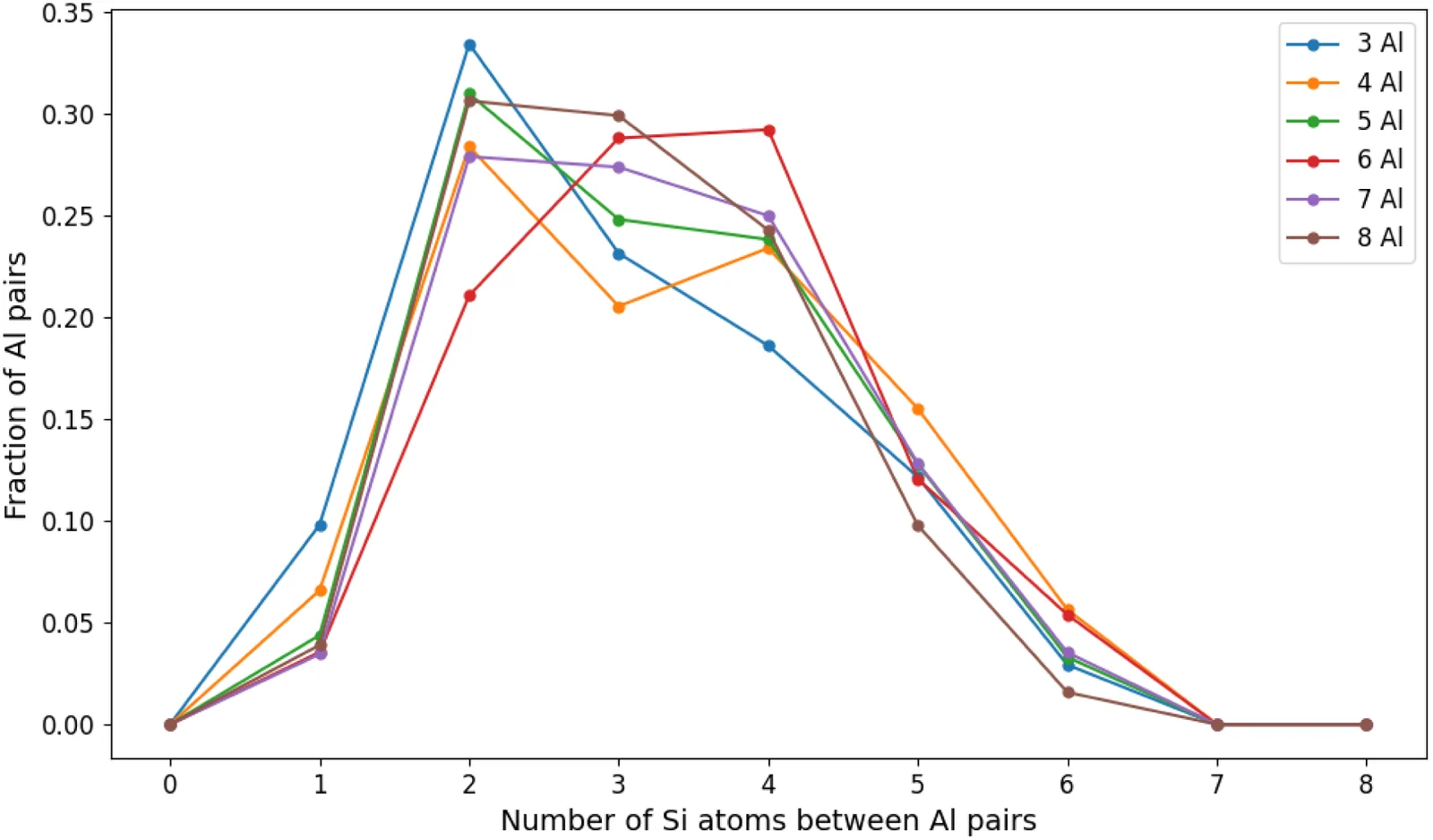
Number of Si atoms separating pairs of Al atoms in zeolite CHA (1 × 2 × 1), calculated over all structures within an arbitrary 30 kJ mol^−1^ of the global minimum, accounting for Boltzmann weighting at 450 K.

As a test of the BH approach, periodic boundary conditions and relaxation effects, we performed extensive studies of 8, 16 and 24 Al atoms in a 2 × 2 × 2 CHA cell and compared the structure and energies of equivalent Si : Al ratios in the 1 × 1 × 1 and 1 × 2 × 1 cell. In line with the 1 × 1 × 1 and 1 × 2 × 1 result for 1 and 2 Al atoms respectively, the 8 Al atom 2 × 2 × 2 cell was found to be in excess of 60 kJ mol^−1^ per cell more stable than a 2 × 2 × 2 expanded 1 Al 1 × 1 × 1 cell due to pairing stabilisation effects. Interestingly, expanding the cell to 1 × 2 × 1 appears to allow sufficient flexibility and relaxation to capture the lowest energy configurations permitted at these higher Si : Al ratios. Taking the global minima for 2 and 3 Al in 1 × 2 × 1 and creating 2 × 1 × 2 supercells to compare with a 2 × 2 × 2 structure revealed that the BH method was unable to find any lower energy structures even after >10^5^ moves.

A further noteworthy point is that there is general interest in answering the question of whether Al tends to segregate or aggregate in zeolites. Answering this question using DFT directly is prohibitively costly but the NNP reveals that for the Si : Al ratios 35–11, the Al tends to aggregate with multiple occupation of D6R's within the same *c*/(001) plane of the cell. Indeed starting from maximally separated Al in the 2 × 2 × 2, the accepted moves played as a trajectory show a systematic decrease in the Al–Al distance as a function of accepted moves as the Al atoms move to the same or neighbouring D6R rings as seen in Fig. 6. Fig. S1 and S2 in the SI (Section S2) show the thermodynamics of incorporating aluminium for 1 × 1 × 1 and 1 × 2 × 1 cells respectively (see S2 for the definition of the incorporation energy). Clearly, the formation of configurations with an even number of aluminum atoms in the cell are significantly more exothermic. The strong effect of pairing aluminium is emphasised by comparing 2 Al in 1 × 2 × 1 *versus* 1 Al in 1 × 1 × 1 that have an identical Si : Al ratio. In 1 × 1 × 1 the energy of incorporation is around −80 kJ mol^−1^ but for 2 Al in 1 × 2 × 1, the incorporation energy is around −110 kJ mol^−1^, showing that pairing in NNN fashion (which is geometrically forbidden in 1 × 1 × 1 due to the unit cell dimensions) releases around 30 kJ mol^−1^. It is noted that increasing the amount of aluminium present in the structure does not become more difficult *i.e.* there is no thermodynamic obstacle (neglecting solvent and SDA contributions) to achieving Si : Al ratios of around 8. This observation is consistent with experimental reports that show Si : Al ratios of approximately 2 are achievable in CHA.^43^

For Na-CHA, there is some limited calorimetry data due to Navrotsky and Tian.^44^ There is a single measurement at exceedingly low Si : Al ratio, Na_0_.459Al_0_.459Si_0_.541O_2_ which estimates the enthalpy of formation from oxides as −49.47 ± 15.9 kJ mol^−1^. For Na_0_.458Al_0.458_Si_0.542_O_2_ (33 aluminium and sodium atoms in a 1 × 2 × 1 cell) our lowest energy configuration was found to be −40.96 kJ mol^−1^ in fair quantitative agreement with the experimental measurement.

### 5.2 RHO

Zeolite RHO, a cage-like zeolite which can be used for applications such as CO_2_/CH_4_ separations,^45^ contains a single symmetry distinct T site within the 48 T site unit cell.

The performance of the stepwise approach and BH have been directly compared for this system (see table S8 in SI for more details). The BH structures were found to be substantially lower in energy (between 0.18 and 1.48 eV) indicating the BH approach identifies configurations significantly more representative of the ground state of the system. This contrasts with chabazite, where the stepwise and BH yield almost equivalent global minimum configurations. Clearly, this finding demonstrates the need for unbiased global optimisation approaches in particular systems.

Intriguingly, it was observed that the cell underwent a phase transition, which corresponded to a decrease in cell volume of around 13%. The transition was observed between 2 and 3 aluminium substitutions in the cell, corresponding to Si : Al ratios of 23 and 15 respectively. This phase transition has been observed by others,^46–48^ though to our knowledge, is has not been linked directly with the Si : Al ratio. Notably, the transition was only observed within the BH approach. In the stepwise configuration approach, the structure retained its original framework, biasing a more homogeneous aluminium distribution. Furthermore, it was observed that this phase transition could be reversed by exchanging the sodium for a proton. The homogeneous aluminium distribution as shown in Fig. 11 comprises of aluminium evenly distributed throughout the framework, with the sodium ions preferring to reside within the 6-rings. Conversely, within the BH structures the aluminium is much more clustered as shown in Fig. 10, which is likely mediated by similar charge compensation effects as in CHA.

**Fig. 10:**
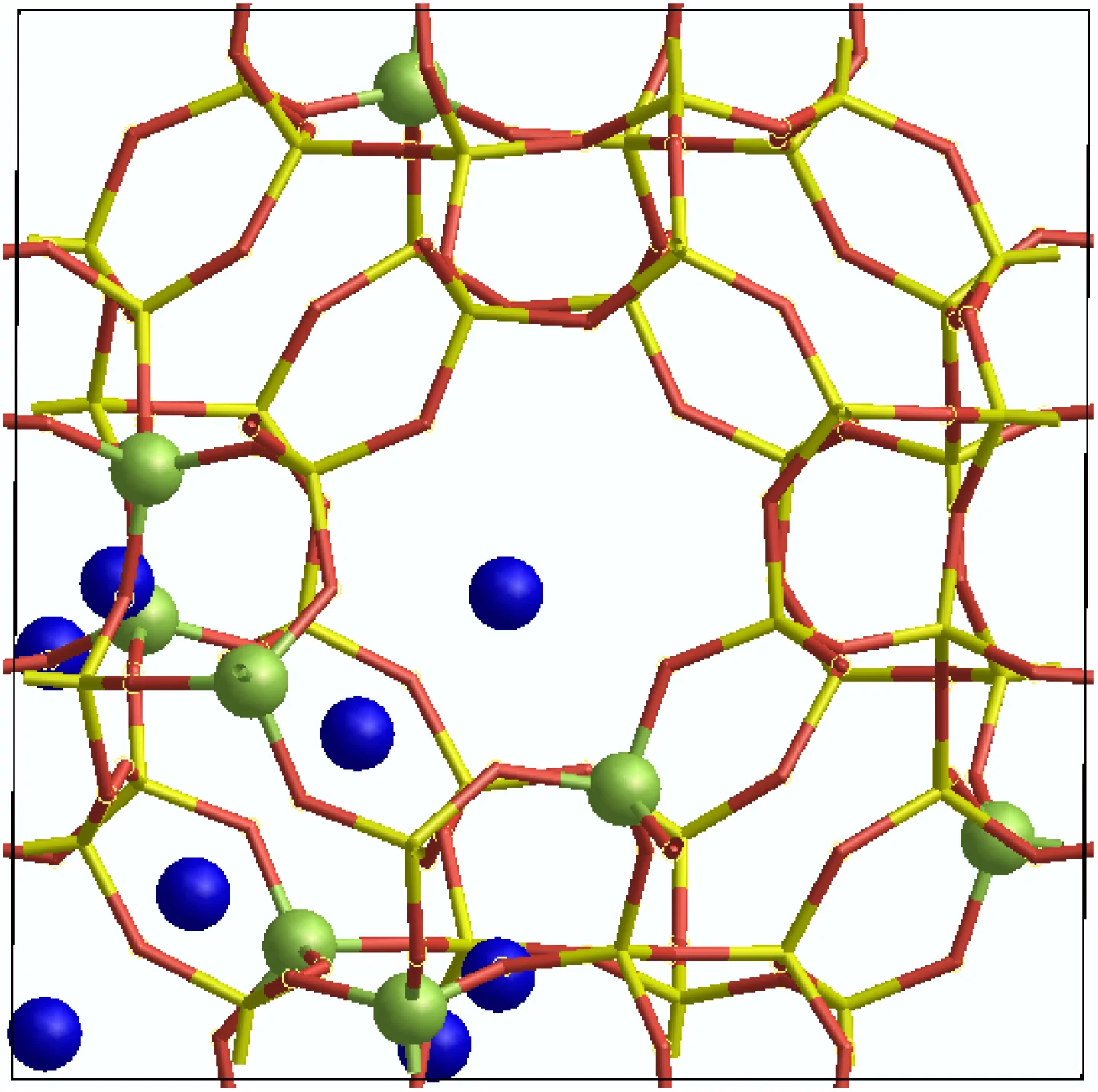
1 × 1 × 1 RHO global minimum with 8 aluminium atoms using basin-hopping. Viewed along the [100] axis. Clearly, the aluminium and sodium is relatively aggregated in comparison to Fig. 11. The puckered framework, due to the phase transition and arising from the higher Al content is also notable in comparison to 11.

**Fig. 11:**
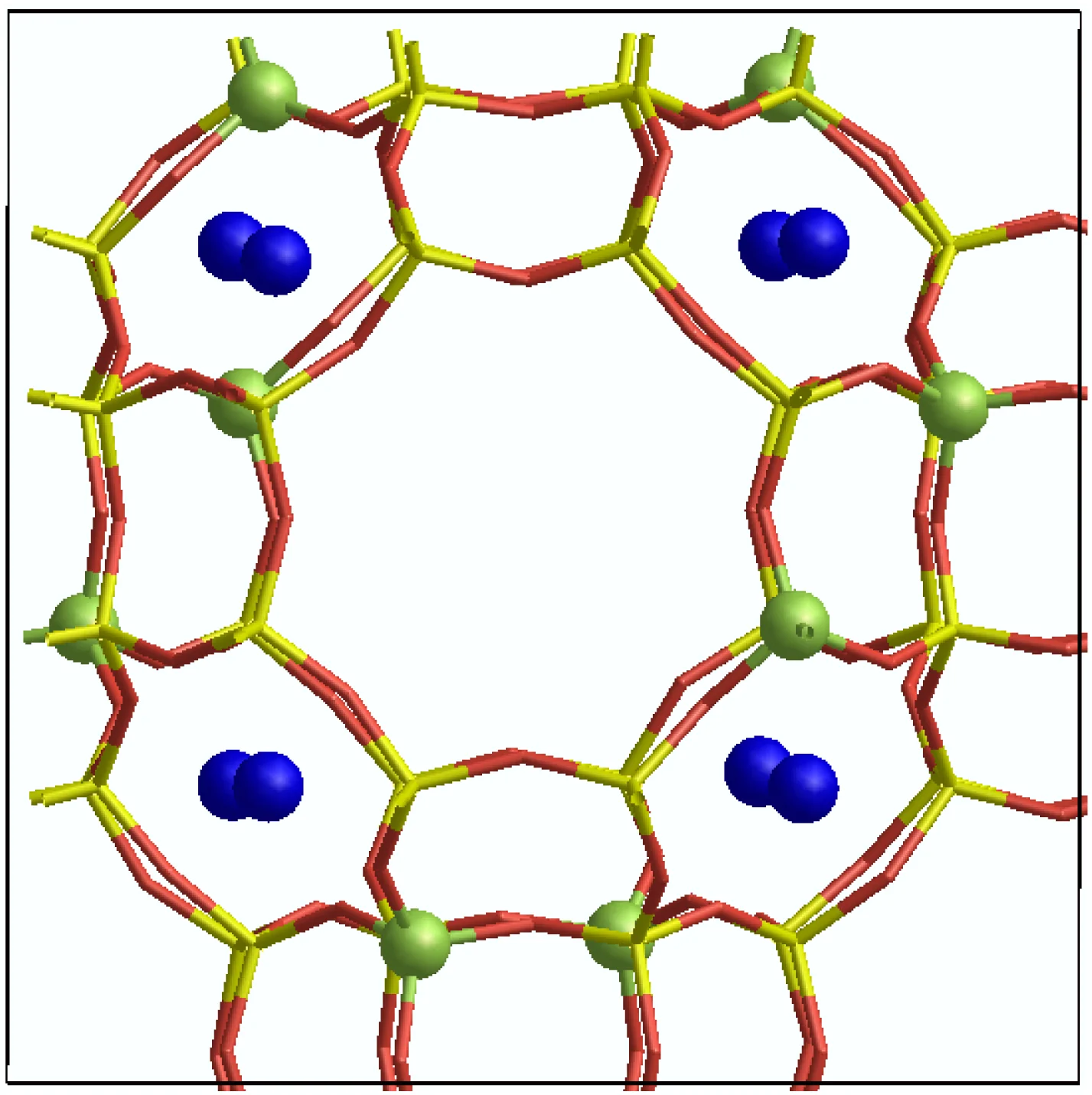
1 × 1 × 1 RHO global minimum with 8 aluminium atoms according to the stepwise algorithm. Viewed along the [100] axis. Interestingly, the structure is less dense than the configuration identified by basin-hopping and clearly the aluminium (green) and sodium (blue) is more homogenously and symmetrically distributed.

Fig. 12 shows an example of the energy landscape for 2 Al in a single unit cell obtained from a complete brute force evaluation of all possible configurations. Whilst the range in energies is very similar to that seen in CHA, the convex hull is notably flatter and a relatively high proportion of the configurations fall within an arbitrary 30 kJ mol^−1^ of the global minimum configuration. Although the lowest energy configurations show a preference for clustering through Al-(O–Si–O)_2_–Al– arrangements (as shown in Fig. 13) (see also S3, the overpopulation assessment in the SI), Fig. 12 shows configurations at much larger Al separations also have competitive energies indicating that at this Si : Al ratio, the aluminium could either be relatively dispersed or more clustered, contrasting to CHA where with 2 Al the Al is almost certainly separated by around 6 Å. Fig. 13 also shows the enhancement of the (Al–O–Si–O–Al–) linkages with increasing concentration of Al, in marked contrast to CHA. These effects can be most readily discerned from overpopulation assessments presented in the SI (Section S3).

**Fig. 12:**
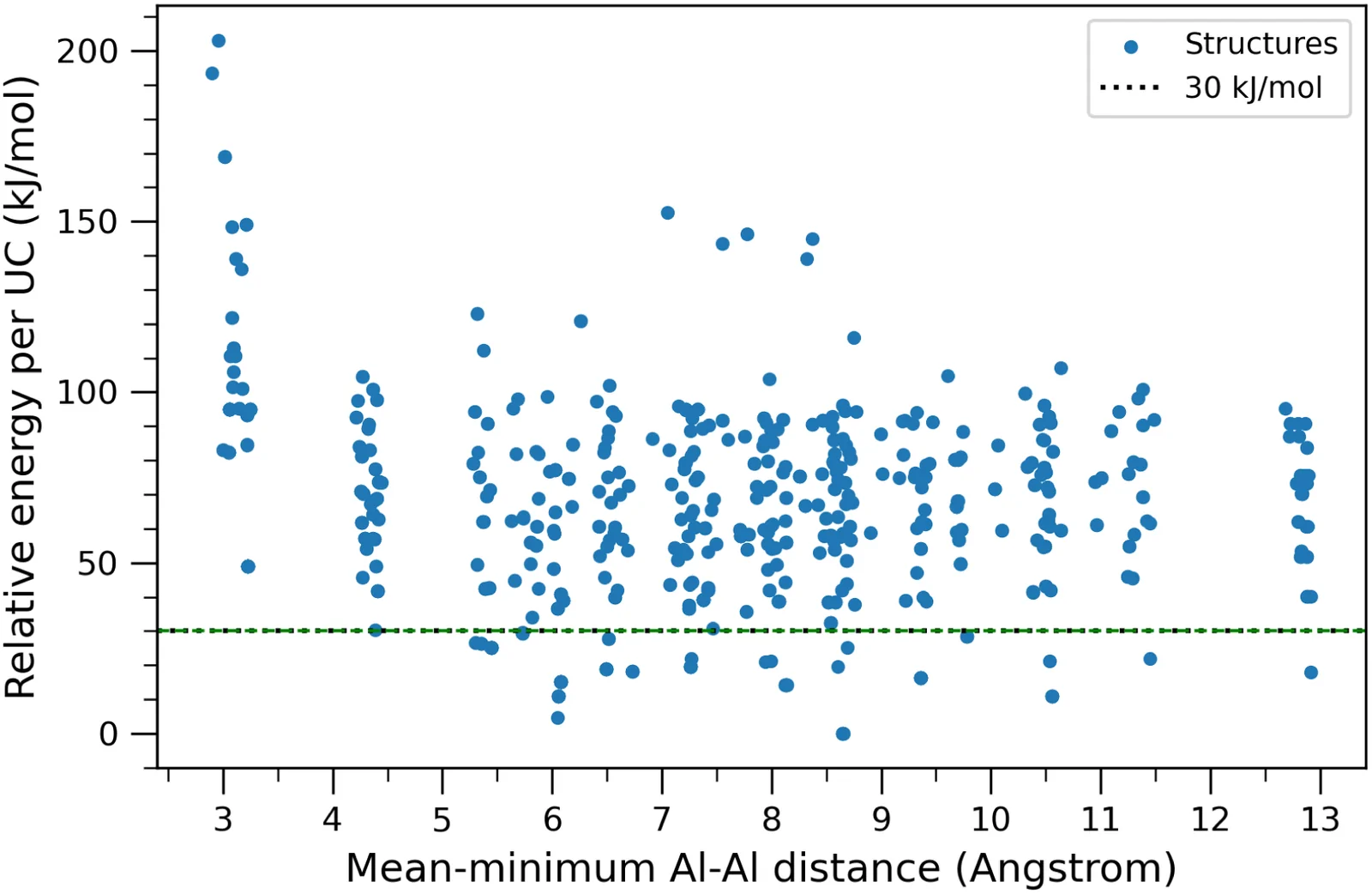
1 × 1 × 1 RHO optimised structure from brute force approach with 2 aluminium atoms, 823 configurations in total and 88 (10.7%) within an arbitrary 30 kJ mol^−1^ of the GM.

**Fig. 13:**
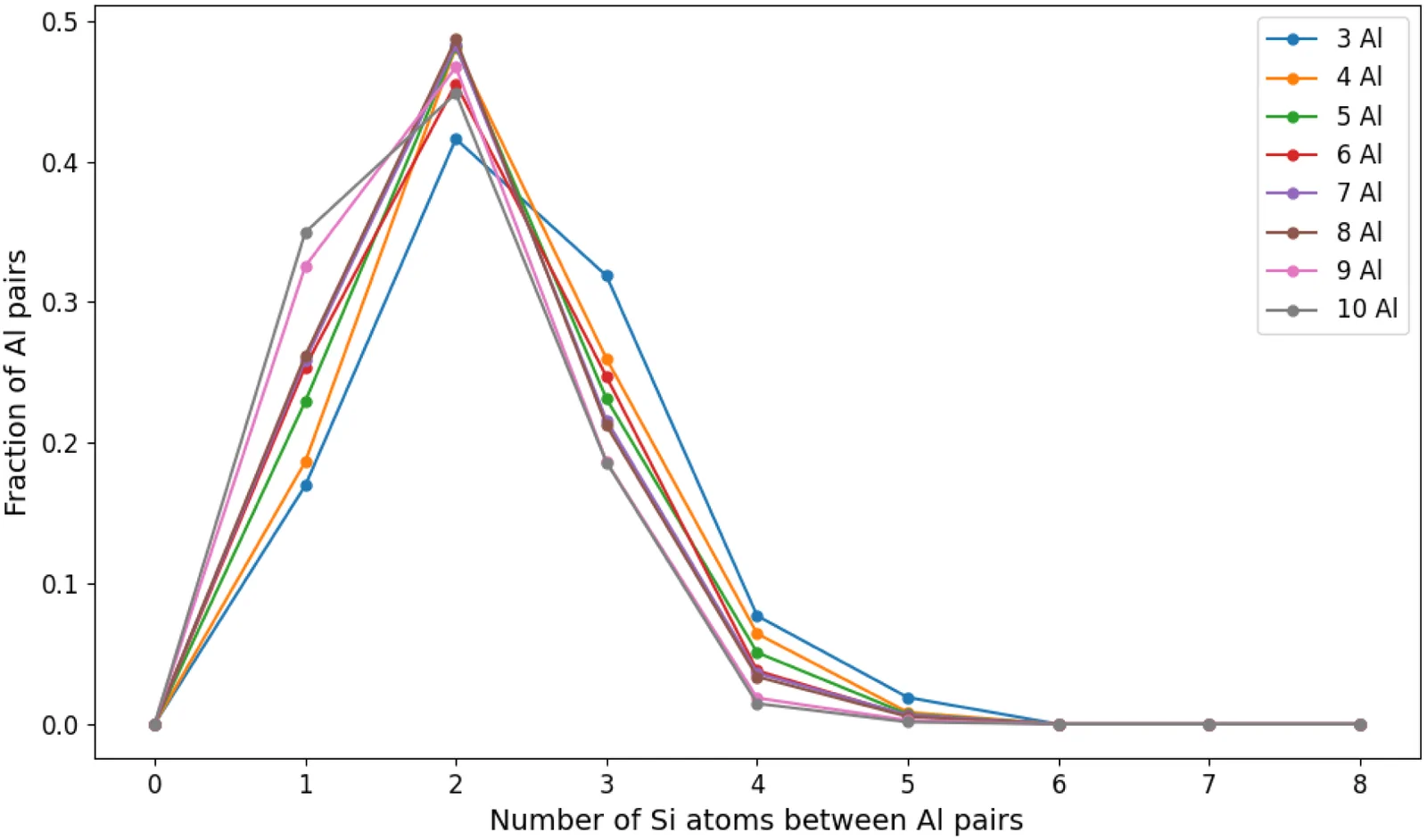
Number of Si atoms separating pairs of Al atoms in zeolite RHO (1 × 1 × 1). An increasing concentration of aluminium results in an increasing concentration of pairs of aluminium separated by one or two silicon atoms.

Addition of Al is found to be favourable as shown in Fig. S3 in the SI, with Si : Al = 11 (4 Al) being notably more exothermic than previous substitutions, which is presumably driven by the volumetric decrease. Further addition of Al, gives rise to approximately the same magnitude of incorporation energy.

### 5.3 MOR

Mordenite differs from CHA and RHO in that there are 4 distinct T-sites rather than a single distinct T site. We considered a 1 × 1 × 2 supercell of MOR since a single unit cell is much shorter along the *c* axis, which would inhibit relaxation unduly. Similar to CHA, the distribution appears to be governed by charge compensation effects; the Al preferring to cluster such that the coordination of the sodium is optimised Fig. 15.

The appearance of Fig. 14 is noticeably flatter than similar plots for the cage-like CHA and RHO structures; there is a band of configurations from around 4.5 Å to around 15.5 Å which have very similar energies, which would appear to suggest that this layered structure allows for far more distributed aluminium. However, Fig. 15 would suggest that for higher aluminium content a degree of clustering is thermodynamically preferred, similar to CHA and RHO, with the aluminium forming “pairs” that extend into chains which span the entire length of the cell.

**Fig. 14:**
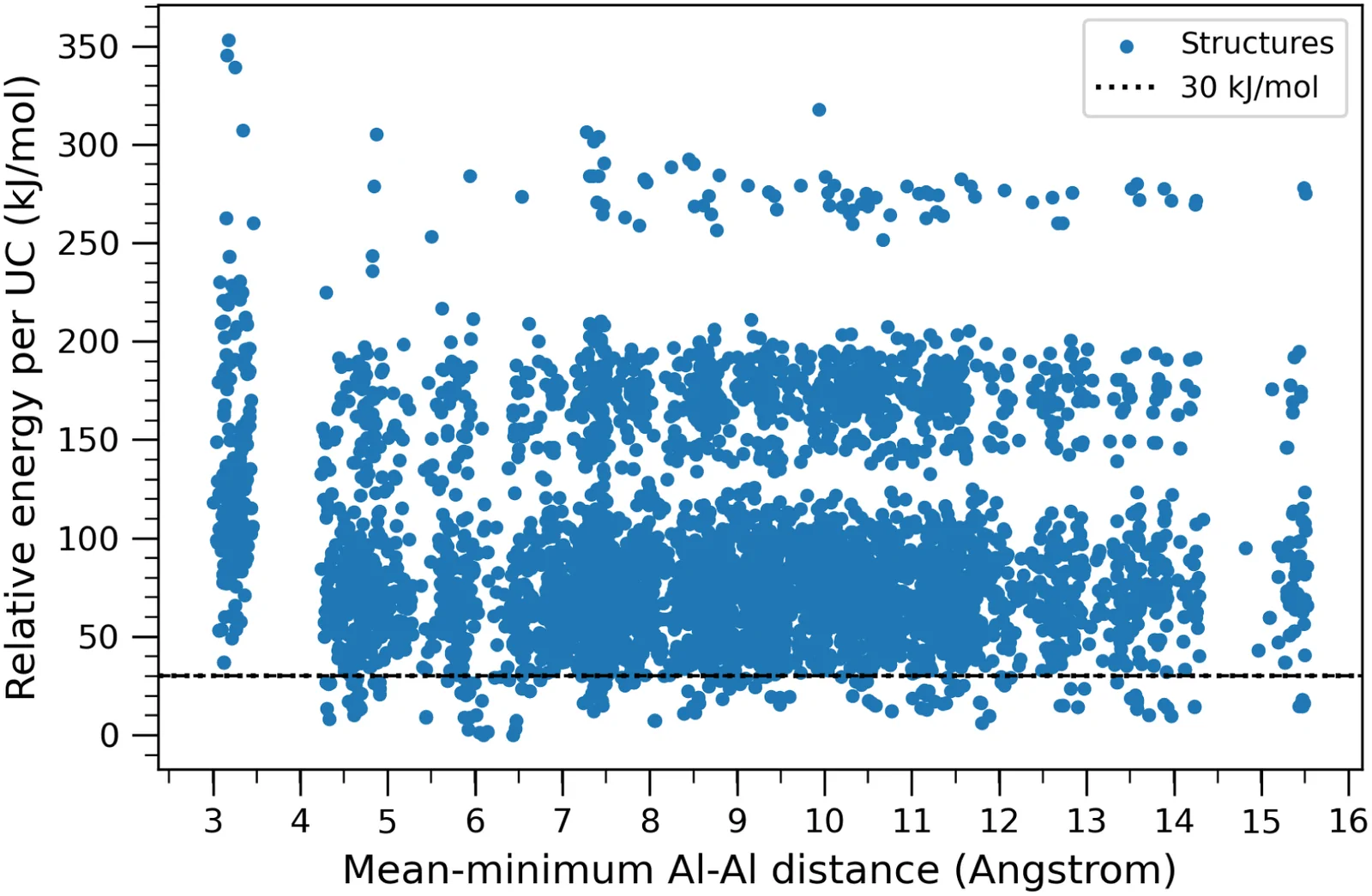
1 × 1 × 2 MOR optimised structures from brute force approach with 2 aluminium atoms, 6608 structures in total and 273 (4.13%) within an arbitrary 30 kJ mol^−1^ of the GM.

**Fig. 15:**
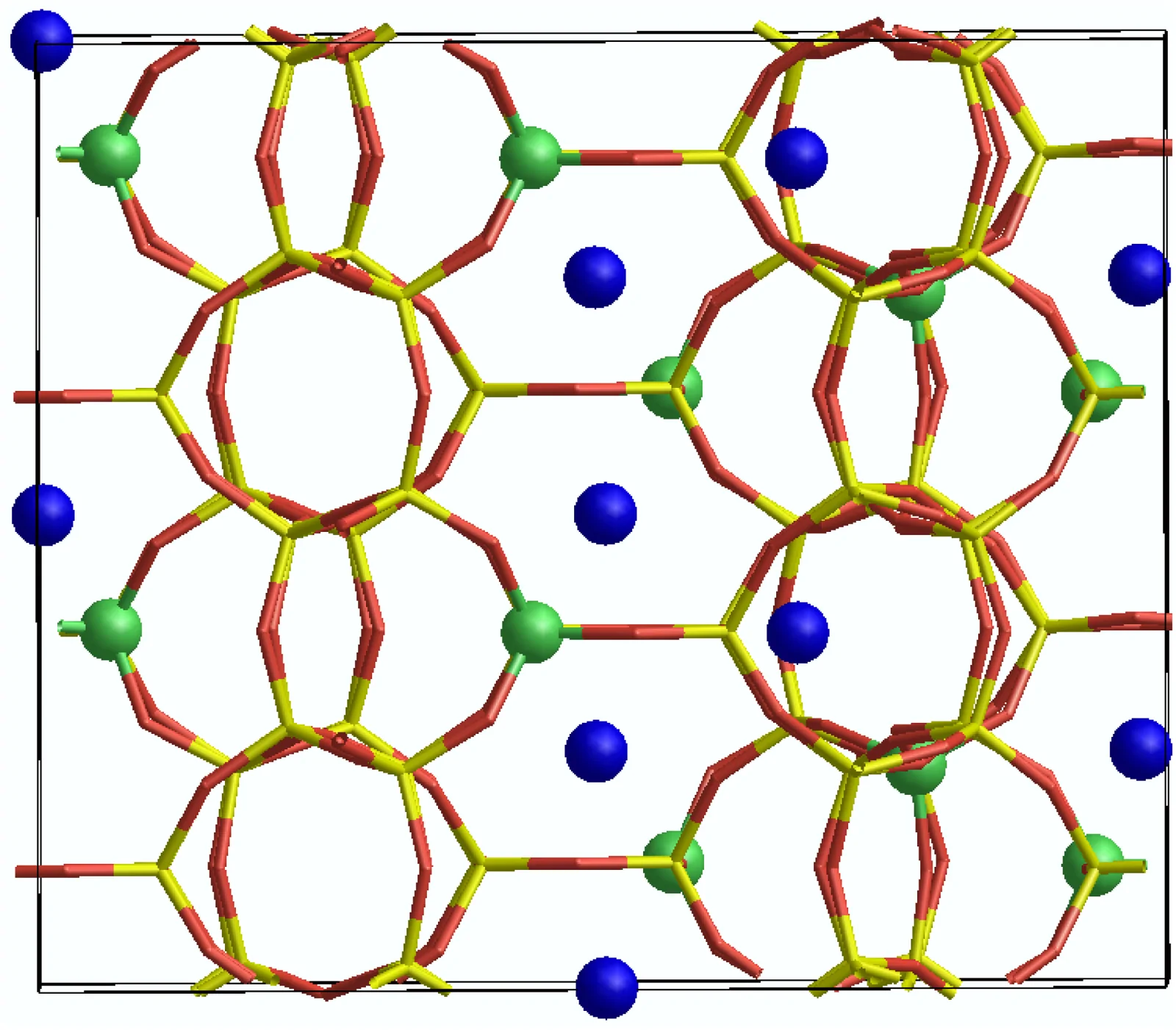
1 × 1 × 2 MOR global minimum with 10 aluminium atoms. Viewed along the [010] axis. The blue sodium ions are organised, forming chains along the *b* axis.

One noteworthy feature of the lowest energy structure identified through brute force methods *versus* BH, is that the structures were qualitatively distinct and the BH GM structure was also found to be more than 0.3 eV lower in energy than the brute force configuration. The BF structure featured one Al in a 5 ring and 1 in a 4 ring (T1 and T3 respectively) whereas BH finds two Al in 4 rings in T3 positions. We speculate that the multiple substitutions accepted in the BH run lead to relaxations and symmetry breaking, that permit a lower energy structure to be found. Moreover, within the BF approach, although all aluminium distributions are exhaustively sampled, the corresponding sodium positions are still constrained to the BVP positions. It is possible that within BH approaches, slightly different positions are sampled since although BH also uses the BVP method to generate sodium positions, it is generating these BVP positions based on different relaxed configurations, *e.g.* the last accepted move. Thus perhaps in BH, slightly better sodium positions are sometimes found compared to BF and/or the backbone structure has broken symmetry or undergone a relaxation that is forbidden due to higher symmetry/cancellation of forces in the BF approach.

As mordenite has 4 inequivalent T sites, we can assess whether there is a strong driving force to occupy particular T sites in the global minima as a function of increasing Al content. To help interpret the spatial distribution of aluminium the distinct T sites are distinguished in Fig. 18.

At maximum dilution, 1 aluminium, the T1 position is preferred, however the T3 site is almost isoenergetic (T3 is metastable with respect to T1 by less than 0.03 eV which is within the error bar associated with the NNP) and hence when configurations within 30 kJ mol^−1^, are considered and Boltzmann weighted, there is an almost even split between T1 and T3 (as can be seen in Fig. 16). Thereafter, with increasing aluminium content, the T3 position in the 8 MR is exclusively preferred until the Si : Al ratio goes to 5 and then 9 when the T1 position is again, selectively occupied in the 12 MR, as shown in Fig. 16.

**Fig. 16:**
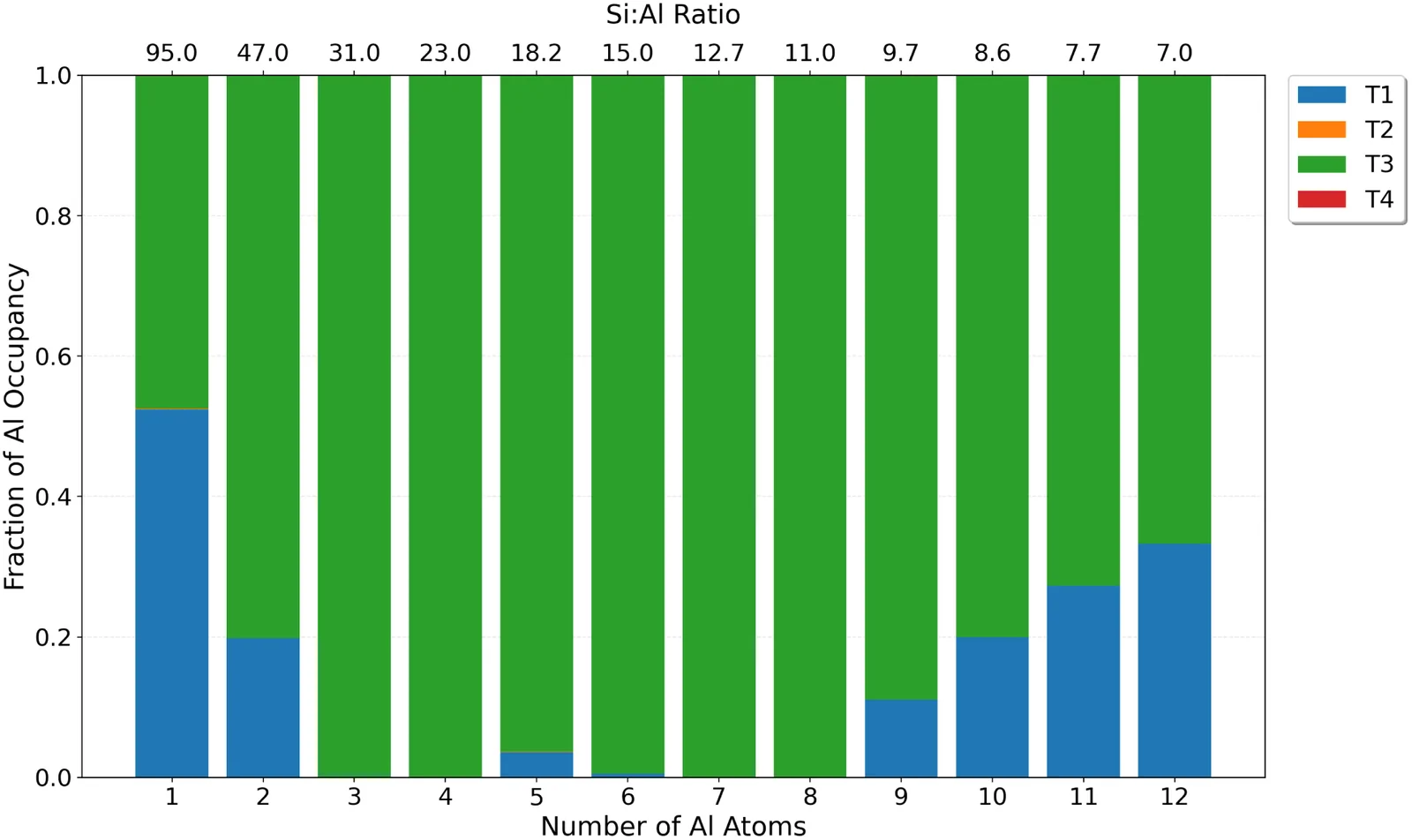
Symmetry inequivalent T site occupation as function of aluminium content for all configurations within an arbitrary 30 kJ mol^−1^ of the global minimum found, weighted by the Boltzmann factor at 450 K.

Our results are perfectly consistent (even quantitatively) with the periodic DFT studies of Antúnez-García *et al.*^49^ A minor difference between the results of Antúnez-García *et al.* and our findings are that because we use a larger cell, the Al populations at T1 are higher in our case. The selective occupation of T3 is well established^50^ and highly desirable for catalysis purposes.^51–53^

Fig. S4 in the SI shows that the energy of incorporation varies considerably as a function of the amount of Al added. In particular, for 5 and 9 Al atoms, the incorporation energy is notably higher; this is due to Al filling or “saturating” a single 8 MR channel with 4 Al atoms along the [001] or *c* direction and the 5th Al goes into a purely siliceous, unpopulated 8 MR channel. In the case of 9 Al atoms, at 8 Al per 1 × 1 × 2 cell, all the 8 MR channels are populated and the 9th Al goes into the 10 MR ring, which is purely siliceous, hence explaining why incorporation is relatively disfavoured for these Si : Al ratios. A more thorough discussion of the distribution of Al is presented in the SI, with an overpopulation analysis (S3).

Fig. 17 shows quite a variation in the distribution of Al pairs within the structure of MOR at different Al concentrations. In essence, the variation is due to populating channels along the *c* axis in a stepwise manner. Like CHA and unlike RHO, the (Al–O–Si–O–Al) linkages are again strongly disfavoured with a clear preference for forming (Al–(O–Si–O)_2_–Al) and (Al–(O–Si–O)_3_–Al) linkages.

**Fig. 17:**
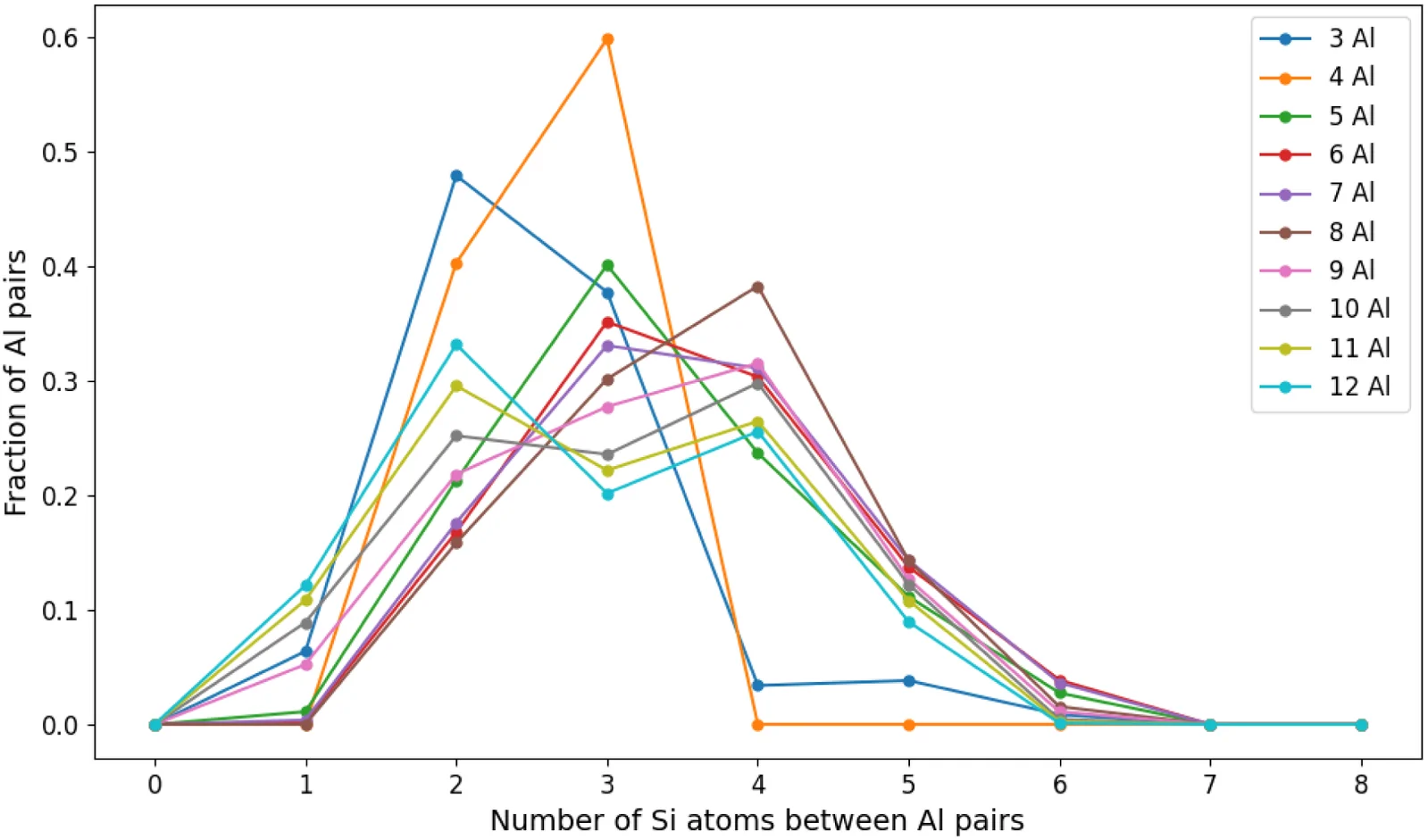
Number of Si atoms separating pairs of Al atoms in zeolite MOR (1 × 1 × 2), for all configurations within an arbitrary 30 kJ mol^−1^ of the global minimum found, weighted by the Boltzmann factor at 450 K.

**Fig. 18:**
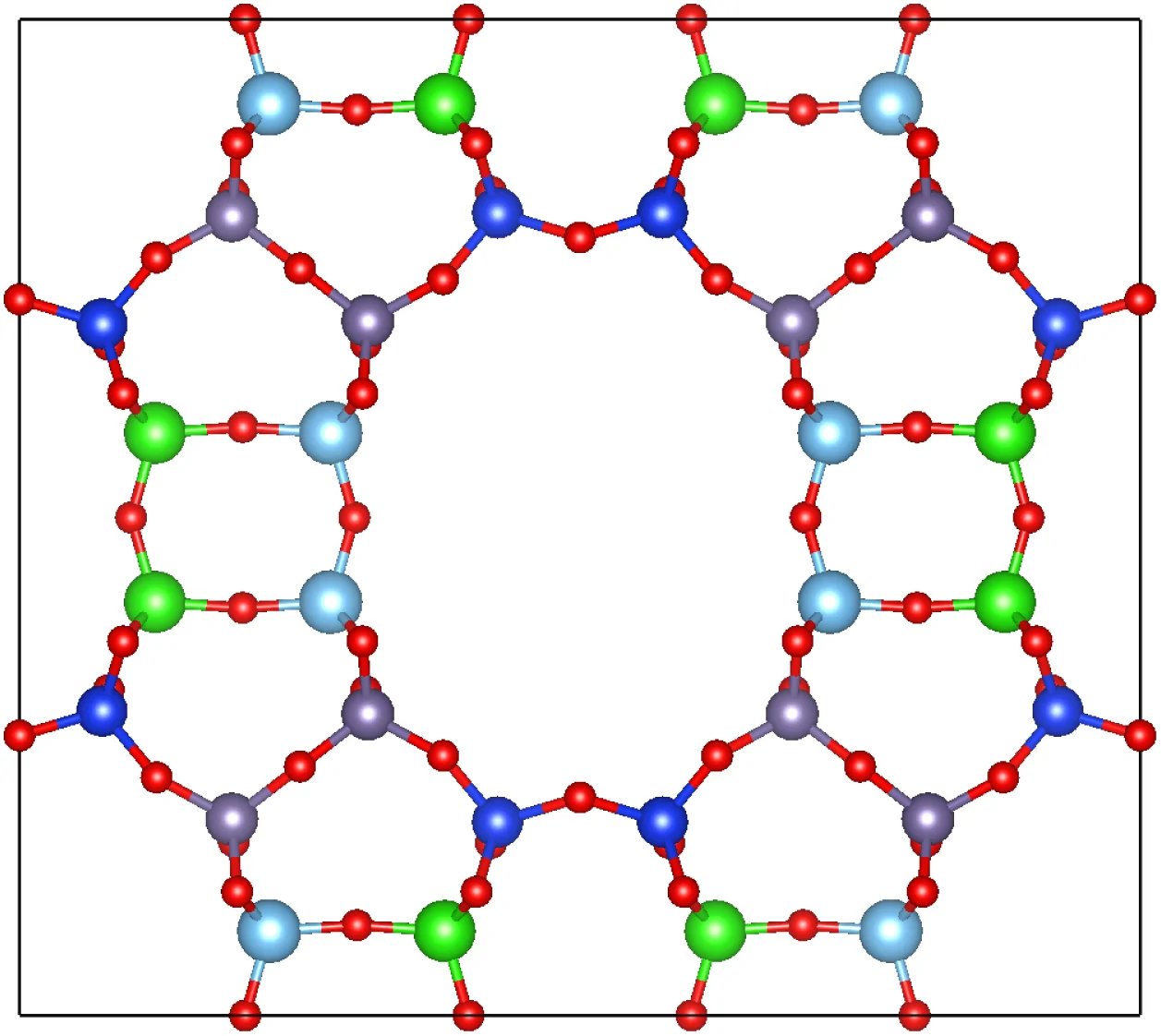
Symmetry distinct T sites for Mordenite (viewed along the [001] axis). T1 is dark blue, T2 is mauve, T3 is green and T4 is cyan.

## 6 Conclusions

We have developed a novel approach to determining the distribution of aluminum in zeolites by combining neural network potentials with a custom basin-hopping algorithm that we believe efficiently identifies low energy configurations. The approach can be applied to a wide range of Si : Al ratios and topologies beyond those discussed here and constitutes a robust and reliable approach to determining realistic aluminum distributions. Crucially, we have shown that generating distributions at random most likely yields highly metastable structures and that the band of physically relevant configurations is likely very small compared to the full landscape. Qualitative observations are that across all silica Si : Al ratios examined here, there is a tendency for zeolites CHA, RHO and MOR to principally favour –Al–(O–Si–O)_2_–Al– and –Al–(O–Si–O)_3_–Al– sequences, in line with previous findings (see for example Li *et al.*^54^ for a very thorough review) though there are significant differences in distributions for each of these topologies. For example, in the case of RHO, for decreasing Si : Al ratios, there is a shift from –Al–(O–Si–O)_3_–Al– to -Al-(O–Si–O)_2_–Al– and –Al–(O–Si–O)–Al– which is associated with aggregating Al within a supercage. Conversely, for the channel type zeolite MOR, decreasing Si : Al ratios result in more distributed Al with Al present in –Al–(O–Si–O)–Al– to –Al–(O–Si–O)_5_–Al– as the Al favours occupying a single channel before another is occupied. An assessment of T-site occupation in MOR shows the T3 site is selectively occupied in agreement with experimental findings. Notably for this channel type zeolite, in contrast to the cage zeolites CHA and MOR, many more configurations are found with similar energies over range of aluminium–aluminium distances, implying a less discriminating topology. It remains to be seen whether this is a general property of channel based zeolites.

We find that working with single unit cells for predicting aluminium distributions can lead to activated structures with (presumably) enhanced reactivity but unrealistically distributed aluminium which would have ramifications for reactivity and screening studies for researchers using periodic *ab initio*/DFT approaches as well as machine learning interatomic potentials.

In general, it appears that there is an almost topology invariant enthalpic benefit for introducing increasing amounts of aluminium into structures which is consistent with calorimetric data.^44^ Zeolite RHO appears to show an increasing enthalpy gain but this appears to be driven by a phase transition accompanied by a very large cell contraction and increase in density associated with increasing Al content.

Future work will include examining different move classes including multiswaps (multiple aluminium and sodium atoms interchanged/moved in a single step) and temperature schedules such as adaptive temperature regimes for improved efficiency in larger systems. Moreover, the effects of hydration, SDA and the extra-framework charge compensating metal cation on the preferred distribution would be of interest, as this would more closely mimic experimental synthesis conditions.

Finally, we note that although we find the number of structures that are stable is low in comparison to possible structures, there are factors we cannot realistically describe through atomistic modelling. Kinetic factors^2,55^ such as rising pH, supersaturation conditions in the gel or solution can give rise to effects such as zoning^56^ with inhomogeneous aluminium distributions, even from crystal to crystal, within a single batch synthesis. Therefore, we see that one of the key uses of the approach described for predicting aluminum distribution in zeolites, is to identify a pool of candidate structures and to take an ensemble approach to studying how variations in structure translate to changes in reactivity and transport properties.

## Author contributions

OB and BS conceived the study with inputs from MS, NC, AE, LG and CJH. Analysis was performed by OB and BS with input from IS, AE, LG and CJH. Funding was acquired by BS, MS and NC. OB and BS wrote the original draft with reviews and editing from all authors. OB wrote the software and OB and BS performed the analyses with input from all authors.

## Conflicts of interest

There are no conflicts to declare.

## Supplementary Material

DD-OLF-D6DD00356G-s001

## Data Availability

The source code for sodium BH along with instructions for running the code are available from https://doi.org/10.5281/zenodo.21775861. The pool of low energy structures for CHA, RHO and MOR are available *via* the data repository at the following link https://doi.org/10.5281/zenodo.21775861. Supplementary information (SI) is available. See DOI: https://doi.org/10.1039/d6dd00356g.
