# Supplementary material for: Prediction of aluminium distributions in zeolites using basin-hopping and machine learning potentials
Source: Digit Discov. 2026 Oct 30. Online ahead of print. doi: 10.1039/d6dd00356g (PMC13721988; doi:10.1039/d6dd00356g)
Supplement: Supplementary file 1 [file DD-OLF-D6DD00356G-s001.pdf]

Supplementary Information (SI) for Digital Discovery.
This journal is © The Royal Society of Chemistry

SUPPORTING INFORMATION

Prediction of aluminium distributions in zeolites using basin-hopping and machine learning potentials

Oscar Bengtsson,[a,*], Daniel Hewitt[a], Indranil Saha[c], Misbah Sarwar[b], Nicola Collis[b], Andreas Erlebach[c],[b] Lukas Grajciar[c], Christopher J. Heard[c] and Ben Slater[a,*]

Contents

1 Counter-cation Positioning **3**
1.1 Molecular Dynamics mobility tests on sodium positions 4

2 Substitution Thermodynamics **4**

3 Structural Analysis **6**
3.1 Analysis of Al distribution separated by Si atoms 7
3.1.1 CHA 1x1x1 overpopulation analysis 8
3.1.2 CHA 1x2x1 overpopulation analysis 9
3.1.3 RHO 1x1x1 overpopulation analysis 11
3.1.4 MOR 1x1x2 overpopulation analysis 13

4 CP2K settings **15**

5 Implementation of Basin Hopping **15**
5.1 Investigating relaxation effects 16
5.2 Move-class details 17
5.3 Improving efficiency 18

6 Sampling **18**
6.1 Assessing convergence 18
6.2 Algorithm evolution 19
6.3 Comparison with exhaustive search 20
6.4 Algorithm modification to assess sodium bias 20
6.5 Stepwise configuration approach 21

7 Comparing Topologies **22**

8 Validation of chosen energy window **22**

[a] *Department of Chemistry, University College London, 20 Gordon Street, London WC1H 0AJ, UK.*
[b] *Johnson Matthey Technology Centre, Sonning Common, Blounts Ct Rd, Sonning Common, Reading RG4 9NH, UK*
[c] *Department of Physical and Macromolecular Chemistry, Faculty of Science, Charles University in Prague, 12483, Czech Republic.*

9 Additional Structure Figures **23**

10 Regression **25**

10.1 Coordination Descriptors 25

1 Counter-cation Positioning

Three distinct methods have been explored for selecting the initial position of the sodium counterion: 1) the sum-vector-projection (SVP), 2) the bond-vector-projection (BVP) and 3) face projection (FP). The first two methods are discussed in section 2.1 of the main text, the FP method meanwhile, generates sodium positions by considering each face of the TO4 tetrahedron and generating a sodium position for each. For a given face the sodium position is determined by obtaining the Cartesian vector connecting the central aluminium atom to the geometric centre of the face. This vector is first normalised to unit length and then scaled by 3, defining the sodium position at 3 Å from the aluminium.

In the case of Chabazite the lowest energy position for sodium was found to be identical for the SVP and BVP approaches. We now illustrate the sensitivity of the method by presenting results for other frameworks. Firstly for RHO, table S1 displays the relaxed energies for a single sodium (and a single aluminium) in the framework using the BVP, SVP and FP methods. Similarly, table S2 corresponds to a single sodium and aluminium in a MOR $1 \times 1 \times 2$ supercell, again with BVP, SVP and FP methods. Since MOR has 4 unique T-sites the algorithms were tested across all.

Table S1 Comparison of the total energies in 1x1x1 RHO for different methods, fully cell relaxed structures using the NNP

Configuration	BVP (eV)	SVP (eV)	FP (eV)
1	**-1524.233642578125**	-1524.02685546875	**-1523.8988037109375**
2	-1524.02685546875	**-1524.027099609375**	**-1523.8988037109375**
3	-1523.8988037109375	-1524.02685546875	-1523.8353271484375
4	-1523.8988037109375	-1524.02685546875	-1523.8353271484375
5	-1524.02685546875	N/A	N/A
6	-1523.835327	N/A	N/A

Table S2 Comparison of the total energies in 1x1x2 MOR for different methods, fully cell relaxed structures using the NNP for all 4 aluminium sites

Configuration	BVP (eV)	SVP (eV)	FP (eV)
**Al1**			
1	-3046.212890625	-3046.30053710937	-3045.34838867187
2	-3046.30078125	**-3046.30078125**	-3046.12939453125
3	-3046.30078125	-3045.30932617187	**-3046.33642578125**
4	-3046.30078125	-3046.11499023437	**-3046.33642578125**
5	**-3046.30102539062**	N/A	N/A
6	**-3046.30102539062**	N/A	N/A
**Al2**			
1	-3046.5263671875	-3045.30004882812	-3046.5263671875
2	-3046.16088867187	**-3046.5546875**	-3046.52661132812
3	-3046.1611328125	-3046.52685546875	-3045.30419921875
4	-3046.5263671875	-3046.5263671875	**-3046.55468755**
5	**-3046.52661132812**	N/A	N/A
6	-3045.98291015625	N/A	N/A
**Al3**			
1	-3046.27514648437	**-3046.55517578125**	-3046.27490234375
2	**-3046.55517578125**	-3046.34790039062	-3046.209716796875
3	-3046.10375976562	**-3046.55517578125**	**-3046.55541992187**
4	-3045.232421875	-3046.34765625	-3046.34790039062
5	-3046.34765625	N/A	N/A
6	-3046.41284179687	N/A	N/A
**Al4**			
1	-3046.06640625	-3046.25170898437	-3046.06640625
2	-3046.30029296875	-3046.16235351562	-3045.40576171875
3	-3046.301269531255	**-3046.30712890625**	**-3046.30102539062**
4	-3046.21313476562	**-3046.30712890625**	-3046.13354492187
5	-3046.27294921875	N/A	N/A
6	**-3046.30737304687**	N/A	N/A

1.1 Molecular Dynamics mobility tests on sodium positions

Static structure generation and geometry optimisation methods inherently risk missing global minimum positions. In the latter method, since this approach is downhill only, there is the possibility that deeper minima may exist adjacent to local minima separated by barriers. To probe whether this is a particular hazard, we undertook some tests using high temperature MD to allow for ions to migrate to other, potentially lower energy positions in an unbiased fashion. Arguably, the temperature used is a biasing function but we choose a temperature which is similar to the melting temperature of the material, to allow for accelerated migrations.

It was found that in chabazite, the SVP algorithm accurately identified the most stable sodium position, whereas in RHO it did not. This was determined by optimising all four configurations using the NNP for one aluminium atom in both CHA and RHO frameworks, followed by 30 ps of NPT 2500K molecular dynamics on the lowest-energy configuration. Snapshots were taken every 100 steps, and these configurations were subsequently optimised to check if the sodium ion had found a lower-energy position. In chabazite, no further improvements were observed when the lowest-energy configuration was annealed. However, in RHO, a minimum energy -0.22 eV lower was found, suggesting that this topology required a different algorithm. It was found that the BVP algorithm sampled this lower energy configuration and hence this justifies our selection of this method throughout this work as reported in the main paper. All structure optimisations and molecular dynamics trajectories were computed using the ASE library[1] with the NNP as the energy calculator.

2 Substitution Thermodynamics

In order to estimate the reaction enthalpy and hence thermodynamic cost of introducing aluminium and sodium into a siliceous zeolite framework, we start from the general equation for the first substitution of a single aluminium ion and sodium counterion into the purely siliceous structure;

$$((\Delta H_f(SiAlNaO_2)) + \Delta H_f(SiO_2(quartz))) - ((\Delta H_f SiO_2(zeolite)) + \Delta H_f \frac{1}{2}(Al_2O_3) + \Delta H_f \frac{1}{2} * (Na_2O)) \quad (1)$$

Where $SiAlNaO_2$ is the substituted zeolite phase, SiO_2(quartz) is the ground state of silica at STP, Al_2O_3 is the ground state of alumina at STP, i.e. corundum and Na_2O is an arbitrary ground state simple oxide source of sodium. The published crystal structures for corundum, quartz and sodium oxide were taken from the Materials Project (`www.materialsproject.org`). These were then optimised, along with the siliceous zeolite host, using the NNP.

More generally, for an arbitrary substitution of n Al atoms, this becomes;

$$((\Delta H_f(SiAl_nNa_nO_2)) + \Delta H_f(nSiO_2(quartz))) - ((\Delta H_f SiO_2(zeolite)) + \Delta H_f \frac{n}{2}(Al_2O_3) + \Delta H_f \frac{n}{2} * (Na_2O)) \quad (2)$$

Below are plots (figures S1,S2,S3, S4) showing the formation enthalpies associated with the systematic addition of Al/Na to various topologies. The displayed points are based on the tentative global minimum found for each composition. Comments upon the noteworthy features are given in the captions.

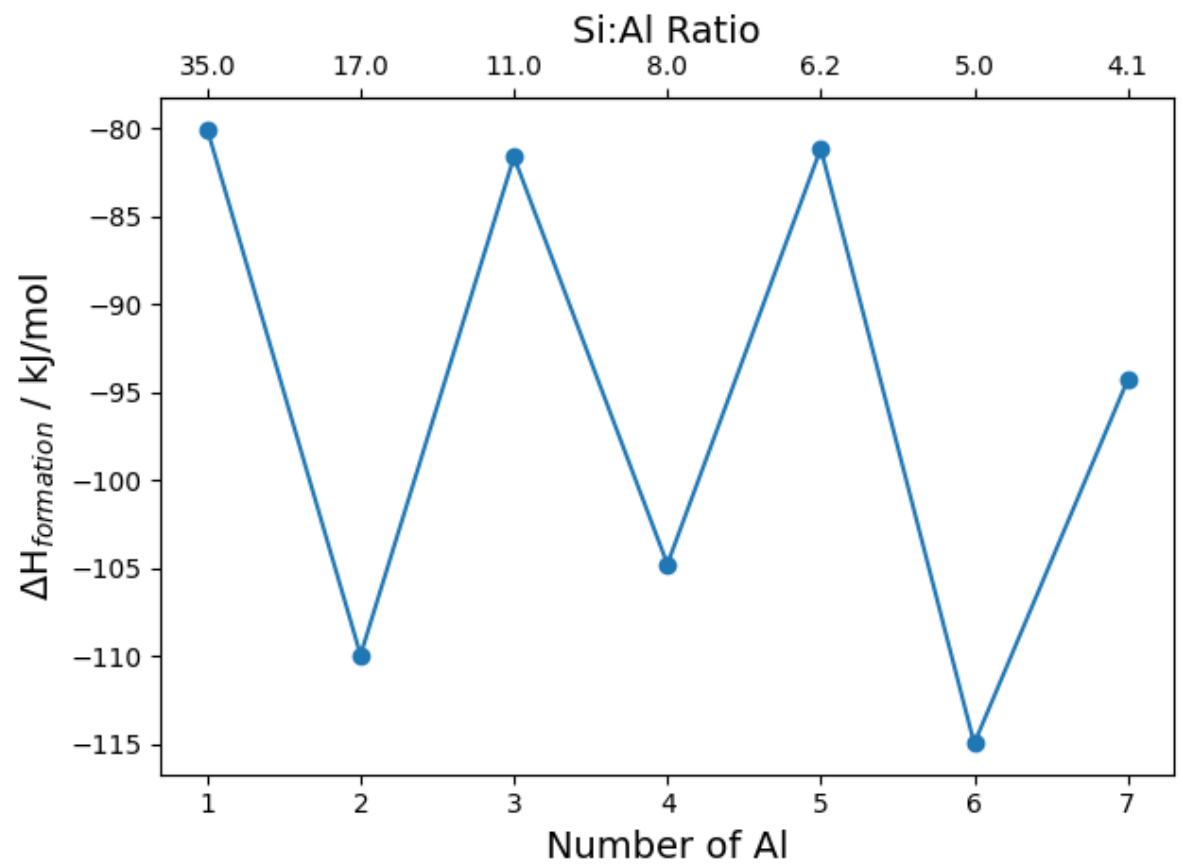

Fig. S1 Enthalpy change of formation for adding increasing amounts of aluminium in zeolite CHA (1x1x1). The Si:Al ratio is given above the figure for reference. An odd even effect is clearly visible where even numbers of Al are associated with occupation of the same S6R.

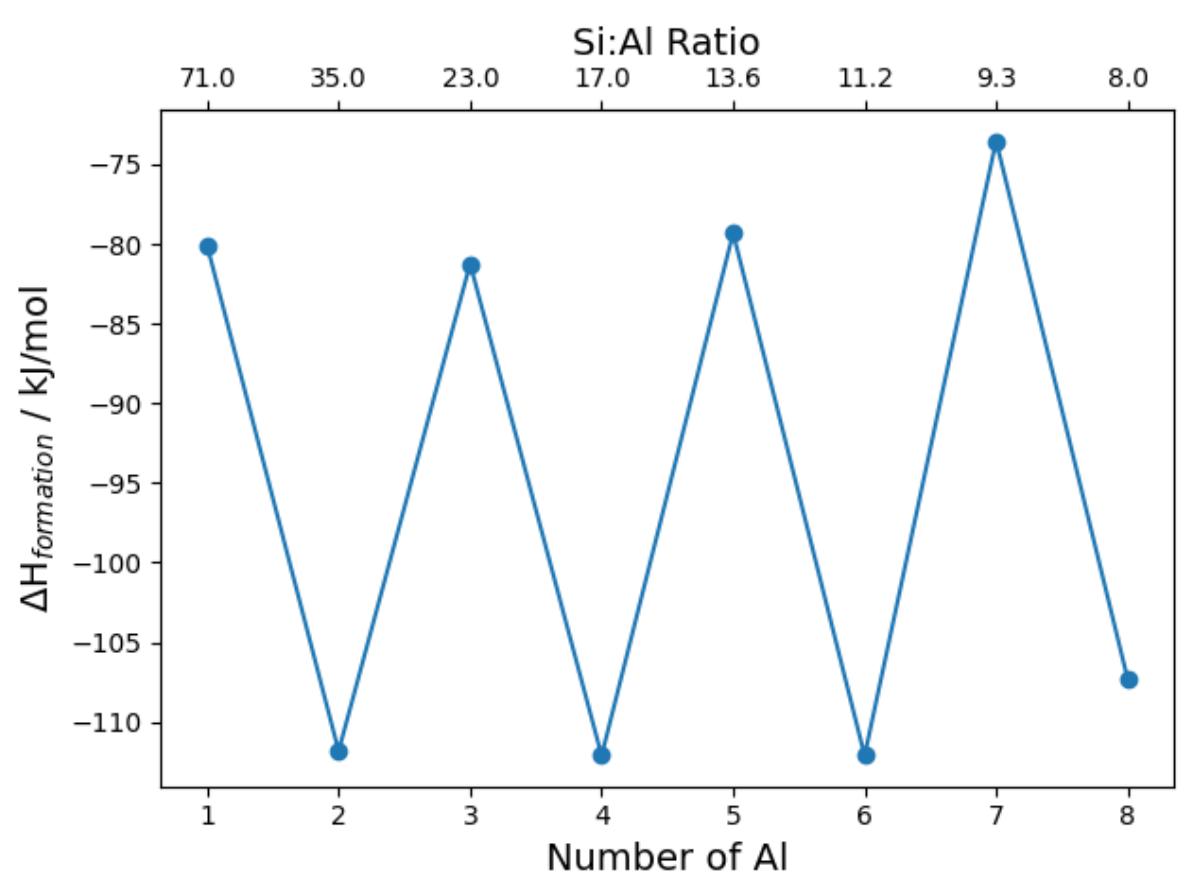

Fig. S2 Enthalpy change of formation for adding increasing amounts of aluminium in zeolite CHA (1x2x1).The Si:Al ratio is given above the figure for reference. An odd-even effect is again evident. Quantitatively, there is little difference between the absolute enthalpy changes for odd or even incorporation, though, for 4Al, the energy of incorporation is more than 5 kJ/mol more stable as the separation between the 2x2 Al motifs is larger in the 1x2x1 case.

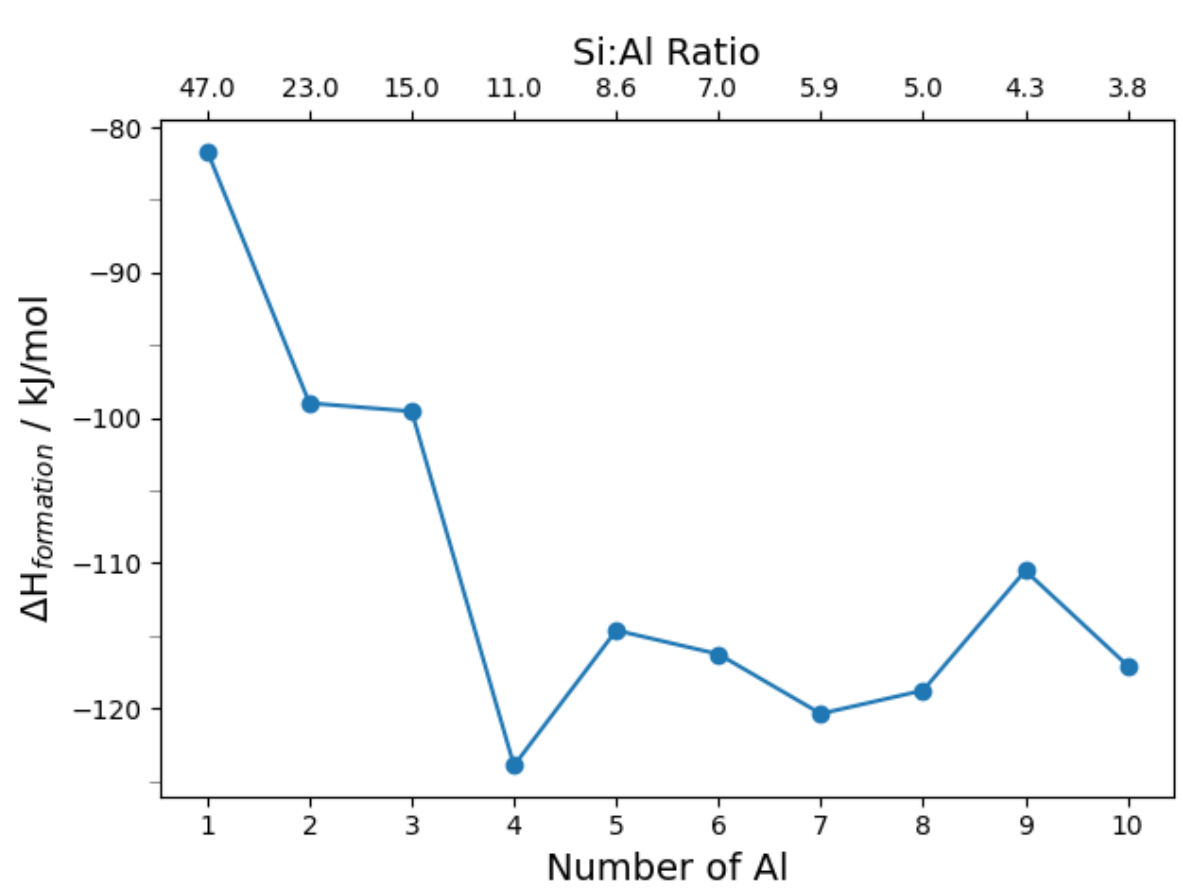

Fig. S3 Enthalpy change of formation for adding increasing amounts of aluminium in zeolite RHO 1x1x1. The energy of incorporation generally increases with increasing Al, due in part to a gradual contraction of the cell.

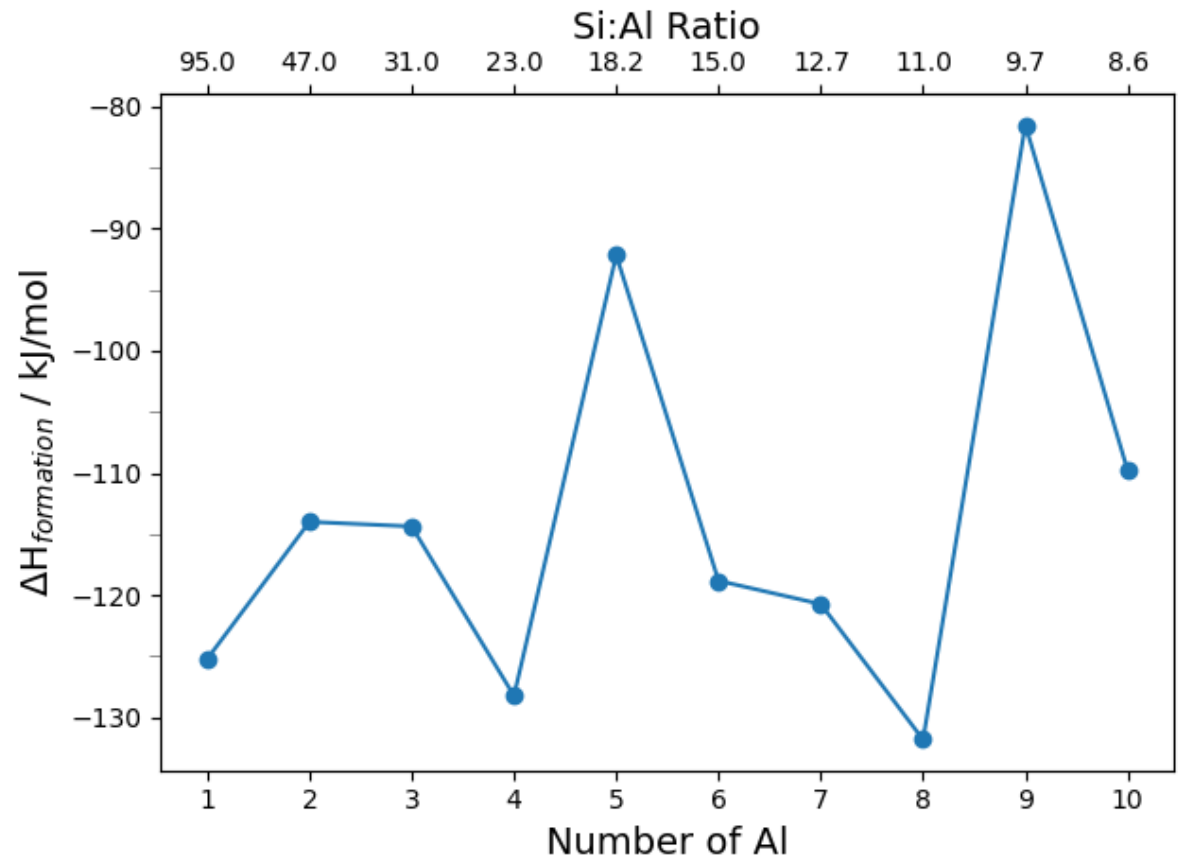

Fig. S4 Enthalpy change of formation for adding increasing amounts of aluminium in zeolite MOR (1x1x2). Odd-even effects are not evident as there is no evidence of preferred pairings between aluminium but instead there is selective occupation of channels as discussed in the main paper. The points at 5 and 9 Al are particularly endothermic and this is due to new channels being occupied. 1-4 Al occupy a single channel and 5-8 occupy a distinct channel. 9-10 occupy a distinct channel hence the relatively endothermic cost associated with 9 Al atoms.

3 Structural Analysis

The structural parameters associated with each putative global minimum structure for CHA (1x1x1), CHA (1x2x1), RHO (1x1x1) and MOR (1x1x2) are reported in tables S3, S4, S5 and S6. Noteworthy features are discussed in the captions.

**File**	**a** (Å)	**b** (Å)	**c** (Å)	α (°)	β (°)	γ (°)	Vol (Å^3)	Si–O mean	Si–O std	Al–O mean	Al–O std	Si–O–Si mean	Si–O–Si std	Si–O–Al mean	Si–O–Al std
CHA_1x1x1_1al.cif	13.55	13.58	14.84	90.14	90.01	119.52	2376.7	1.608	0.011	1.726	0.028	148.53	3.59	145.21	3.43
CHA_1x1x1_2al.cif	13.62	13.51	14.91	89.82	90.18	119.78	2382.4	1.608	0.008	1.732	0.018	148.56	4.46	143.24	5.69
CHA_1x1x1_3al.cif	13.73	13.56	14.89	90.03	90.30	120.30	2392.6	1.610	0.012	1.732	0.022	148.49	5.32	143.45	7.56
CHA_1x1x1_4al.cif	13.45	13.64	15.08	89.71	90.00	119.54	2405.9	1.610	0.016	1.734	0.020	148.18	5.92	144.17	6.68
CHA_1x1x1_5al.cif	13.76	13.45	15.06	89.87	90.53	119.85	2416.7	1.612	0.017	1.728	0.022	148.50	6.46	143.57	7.76
CHA_1x1x1_6al.cif	13.49	13.27	15.49	89.73	91.01	120.06	2399.1	1.610	0.013	1.738	0.013	149.28	9.72	143.45	11.44
CHA_1x1x1_7al.cif	13.98	13.98	12.85	89.95	90.68	120.50	2163.0	1.615	0.019	1.740	0.020	143.79	15.06	139.30	14.50

Table S3 CHA global minima structure analysis summary (1x1x1 cell). Examining the lattice parameters first, it can be seen that whilst a generally increases as does b there are some anomalies, particularly at 4, 6 and 7 Al. There is a very substantial contraction in c at 7 Al but a large expansion in a and b while α , β and γ show relatively minor deviations. For 2-5 Al there is a volume increase, as is expected for the larger aluminium ion but at 6 and 7 aluminium there is a volume decrease, which suggests some level of geometric frustration or possibly the identified structure may not be the global minimum. The Si-O and Al-O bonds all get longer with the introduction of more aluminium, as is expected when the larger aluminium ion replaces the silicon. There is a marked contraction in Si-O-Si-O and Si-O-Al angles and the standard deviation in angles becomes notably larger for 5, 6 and 7 aluminium atoms that potentially signals some level of geometric frustration.

**File**	**a** (Å)	**b** (Å)	**c** (Å)	α (°)	β (°)	γ (°)	Vol (Å^3)	Si–O mean	Si–O std	Al–O mean	Al–O std	Si–O–Si mean	Si–O–Si std	Si–O–Al mean	Si–O–Al std
CHA_1x2x1_1al.cif	13.58	27.13	14.82	89.98	89.94	119.75	4740.3	1.608	0.008	1.727	0.029	148.66	2.60	145.15	3.80
CHA_1x2x1_2al.cif	13.55	27.20	14.85	89.98	89.91	119.85	4746.6	1.608	0.007	1.733	0.019	148.64	3.19	143.59	6.08
CHA_1x2x1_3al.cif	13.62	27.18	14.88	90.04	90.08	120.24	4757.0	1.608	0.010	1.731	0.023	148.53	4.15	144.32	7.24
CHA_1x2x1_4al.cif	13.52	27.25	14.91	89.78	90.18	119.81	4768.3	1.608	0.008	1.733	0.019	148.53	4.19	143.72	6.46
CHA_1x2x1_5al.cif	13.53	27.24	14.93	89.76	90.12	119.64	4782.8	1.609	0.017	1.731	0.023	148.40	4.74	144.08	6.20
CHA_1x2x1_6al.cif	13.50	27.27	14.98	89.74	90.11	119.70	4790.7	1.609	0.011	1.733	0.019	148.37	5.02	144.21	6.86
CHA_1x2x1_7al.cif	13.56	27.27	14.95	89.92	89.85	119.72	4800.2	1.610	0.012	1.733	0.021	148.34	5.63	144.25	7.17
CHA_1x2x1_8al.cif	13.42	27.18	15.17	89.81	89.73	119.60	4810.8	1.609	0.011	1.735	0.019	148.64	6.07	143.94	9.25

Table S4 CHA global minima structure analysis summary (1x2x1 cell). Unlike the (1x1x1) results, the volume now systematically increases with increasing aluminium content. If we examine the Si-O and Al-O bonds, the expansion of both sets of bonds is less marked than (1x1x1) pointing to a level of geometric frustration imposed within the smaller unit cell. It is also very noticeable that the mean and standard deviation for Si-O-Si and Si-O-Al is much less pronounced than in 1x1x1. Despite the volume increasing reasonably uniformly with aluminium content, the cell parameters do show some minor contractions, which may reflect that larger cells still need to be considered to smoothly capture longer ranged relaxation effects. We should also note that the stress tensor was not a fitting variable in the development of the NNP and hence this may have an effect here.

File	a	b	c	α	β	γ	Vol	Si–O		Al–O		Si–O–Si		Si–O–Al	
	(Å)	(Å)	(Å)	(°)	(°)	(°)	($Å^3$)	mean	std	mean	std	mean	std	mean	std
RHO_1x1x1_1al.cif	14.91	14.91	14.91	90.02	90.05	90.05	3315.8	1.609	0.009	1.725	0.018	146.93	2.07	142.14	0.99
RHO_1x1x1_2al.cif	14.93	14.95	14.93	90.00	90.09	90.00	3331.2	1.610	0.011	1.728	0.016	146.92	2.51	142.34	0.93
RHO_1x1x1_3al.cif	14.39	14.42	14.38	89.94	90.19	89.69	2982.4	1.613	0.011	1.736	0.017	139.92	2.40	134.89	3.62
RHO_1x1x1_4al.cif	14.30	14.29	14.41	89.94	90.00	90.00	2945.3	1.614	0.013	1.736	0.019	138.96	2.74	133.76	4.03
RHO_1x1x1_5al.cif	14.33	14.28	14.35	89.75	89.82	90.19	2935.9	1.615	0.012	1.738	0.018	138.74	2.66	133.55	2.60
RHO_1x1x1_6al.cif	14.30	14.29	14.37	89.96	90.31	89.95	2937.2	1.615	0.012	1.739	0.018	138.67	2.79	133.69	3.62
RHO_1x1x1_7al.cif	14.31	14.30	14.28	89.96	90.19	89.77	2923.2	1.616	0.014	1.739	0.015	138.32	2.67	132.63	4.34
RHO_1x1x1_8al.cif	14.28	14.34	14.32	89.63	90.00	90.00	2930.3	1.616	0.016	1.740	0.018	138.10	3.20	133.47	4.05
RHO_1x1x1_9al.cif	14.31	14.32	14.35	90.03	90.03	90.04	2940.4	1.616	0.012	1.738	0.016	138.30	2.86	133.00	3.14
RHO_1x1x1_10al.cif	14.30	14.33	14.28	90.00	89.62	89.99	2927.7	1.617	0.015	1.740	0.016	137.93	3.23	133.44	3.17

Table S5 RHO 1x1x1 structure analysis summary. In line with the reasonably systematic decrease in the cell volume with increasing aluminium content (associated with a phase transition), there are gradual reductions in the cell length parameters, while the cell remains quasi-cubic/orthorhombic. Interestingly, the Si-O and Al-O bonds get longer despite the cell volume shrinking by approximately 10%, while the bond angles decrease but in a modest way. The small standard deviation of bond angles is in marked contrast to CHA which is also a cage zeolite. CHA has two asymmetric cages in the 1x1x1 rhombohedral cell, while RHO has a two supercages that are approximately spherical and hence relatively symmetric. Presumably, the more isotropic RHO cages are able to dissipate strain more effectively than in CHA.

File	a	b	c	α	β	γ	Vol	Si–O		Al–O		Si–O–Si		Si–O–Al	
	(Å)	(Å)	(Å)	(°)	(°)	(°)	($Å^3$)	mean	std	mean	std	mean	std	mean	std
MOR_1x1x2_2Al.cif	18.05	20.12	14.94	90.01	90.01	89.37	5424.8	1.607	0.007	1.742	0.026	151.04	9.48	143.45	16.32
MOR_1x1x2_3Al.cif	18.04	20.06	14.94	90.00	90.01	89.24	5407.4	1.608	0.007	1.741	0.021	150.40	9.87	143.58	16.03
MOR_1x1x2_4Al.cif	18.02	20.05	14.97	90.00	90.00	91.04	5405.4	1.607	0.006	1.746	0.029	151.11	9.65	141.18	18.78
MOR_1x1x2_5Al.cif	17.98	19.93	14.98	89.98	90.07	89.46	5370.0	1.609	0.009	1.734	0.023	149.06	9.83	144.24	15.68
MOR_1x1x2_6Al.cif	17.96	19.88	15.00	90.01	89.98	89.69	5354.4	1.609	0.009	1.735	0.026	148.76	9.91	144.13	15.57
MOR_1x1x2_7Al.cif	17.95	19.84	15.00	90.00	90.00	90.11	5341.1	1.610	0.008	1.739	0.025	148.35	10.38	143.60	15.71
MOR_1x1x2_8Al.cif	17.93	19.80	15.02	90.00	90.00	89.99	5330.4	1.610	0.007	1.739	0.027	148.16	10.28	143.13	16.13
MOR_1x1x2_9Al.cif	17.91	19.80	15.04	89.99	90.04	89.71	5335.5	1.610	0.009	1.741	0.026	148.61	10.32	142.41	16.11
MOR_1x1x2_10Al.cif	17.90	19.78	15.06	90.03	89.89	89.58	5332.0	1.610	0.009	1.740	0.025	148.39	10.32	142.39	16.14

Table S6 MOR 1x1x2 structure analysis summary. It can be seen that a and b contract with increasing aluminium contract while c shows a modest expansion, all accompanied by a small contraction in the cell volume. While the Si-O and Al-O bonds show small expansions, these are comparable with RHO for example. What is particularly noticeable is that the standard deviations in the Si-O-Si and Si-O-Al bond angles are comparatively large, even at maximum dilution of aluminium. Presumably, this is due to the four distinct T sites found in MOR.

3.1 Analysis of Al distribution separated by Si atoms

In the following figures, we compare the probability of finding pairs of Al ions separated by 0 to 8 Si atoms, with different amounts of Al ions. We consider all unique structures within 30 kJ/mol of the global minimum as found by basin hopping and structures are Boltzmann weighted at 450K according to equation 3. All structures are verified for "uniqueness" using `StructureMatcher` within `PyMatgen`.

$$w_i = \frac{e^{-(E_i - E_{\min})/kT}}{\sum_{j=1}^{N} e^{-(E_j - E_{\min})/kT}} \tag{3}$$

Where, w_i is the Boltzmann weight for structure i, E_i is the energy of structure i, $E_{\min} = \min(E_1, E_2, \ldots, E_N)$ is the global minimum energy, k is the Boltzmann constant, $T = 450$K the temperature and N is the total number of structures considered.

The percentage is measured relative to the population of finding pairs averaged over 5000 randomly generated structures (i.e. the appropriate number of Al atoms are randomly substituted in an all silica model). Hence, an under- or overpopulation at particular separation of Si atoms between Al pairs implies a directing or site ordering effect. Note that in the random structure generation, non-Lowenstein structures are forbidden.

In the following figures, blue and positive percentages indicate that the envelope of thermally accessible structures contains a greater probability of finding structures with a given separation of Si atoms between Al pairs compared to a random, unbiased structure. Conversely, a red and negative percentage conveys an under-representation of structures in the thermodynamically accessible envelope of structures with a given number of Si atoms between Al pairs, compared to a random distribution.

3.1.1 CHA 1x1x1 overpopulation analysis

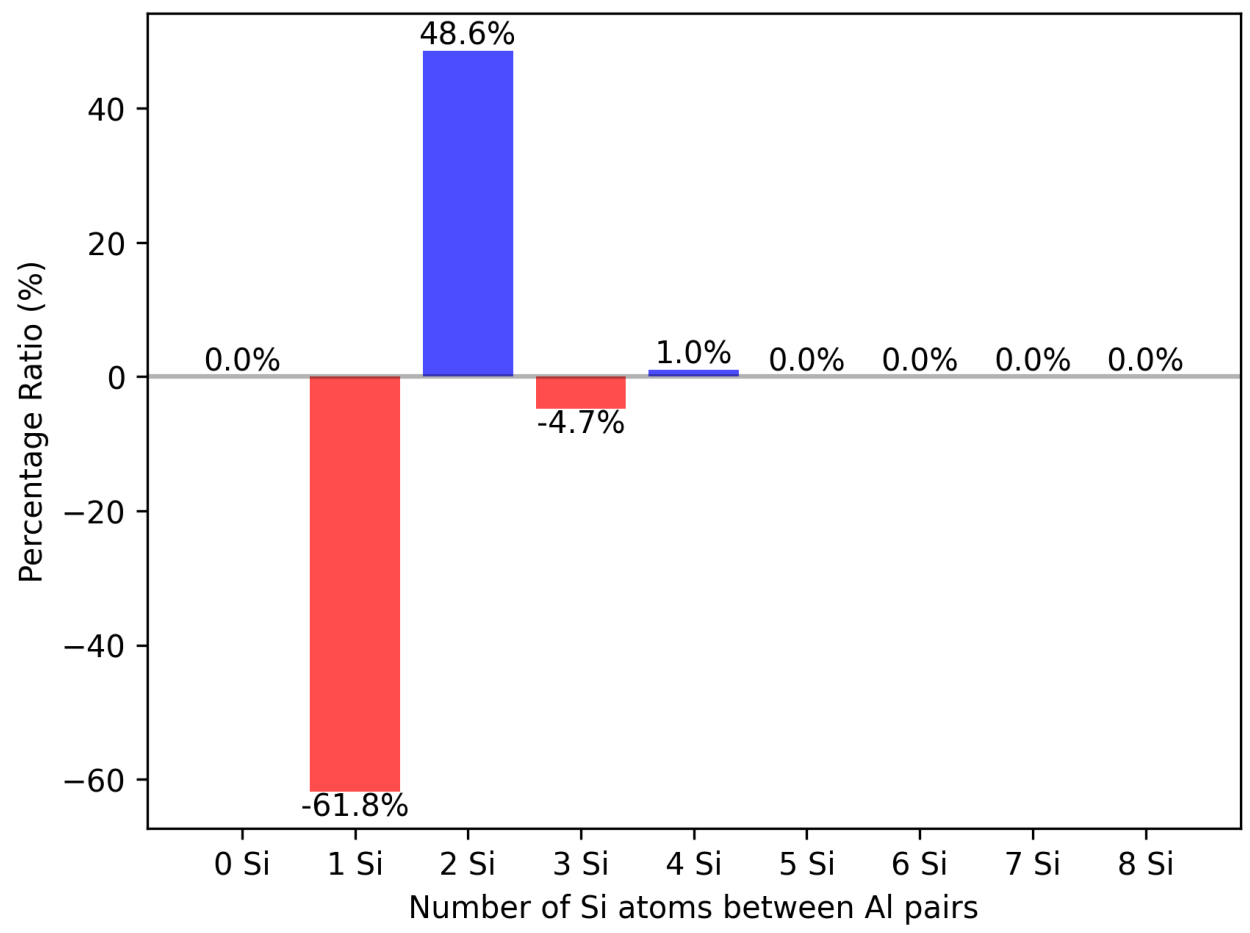

Fig. S5 3 Al CHA 1x1x1 overpopulation analysis. 1 and 3 Si atoms between Al pairs are disfavoured in the thermodynamically accessible structures whereas Al-Si-Si-Al sequences dominate significantly, weak preference for 4 Si separations.

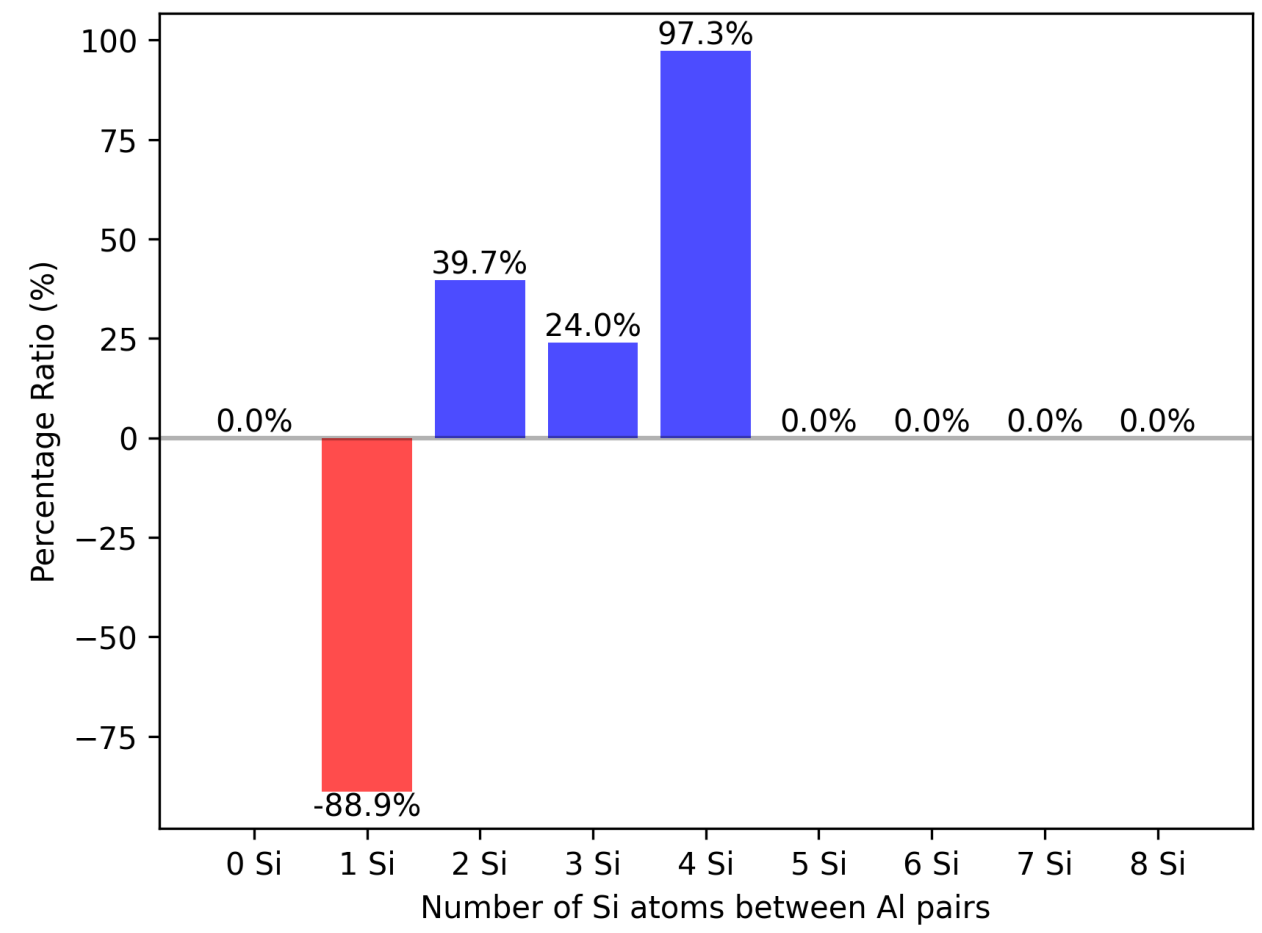

Fig. S6 4 Al CHA 1x1x1 overpopulation analysis. Here there is a clear, strong disincentive to form Al-Si-Al linkages, and instead a preference to form structures with larger separations between Al pairs, but preferably Al-Si-Si-Si-Al linkages.

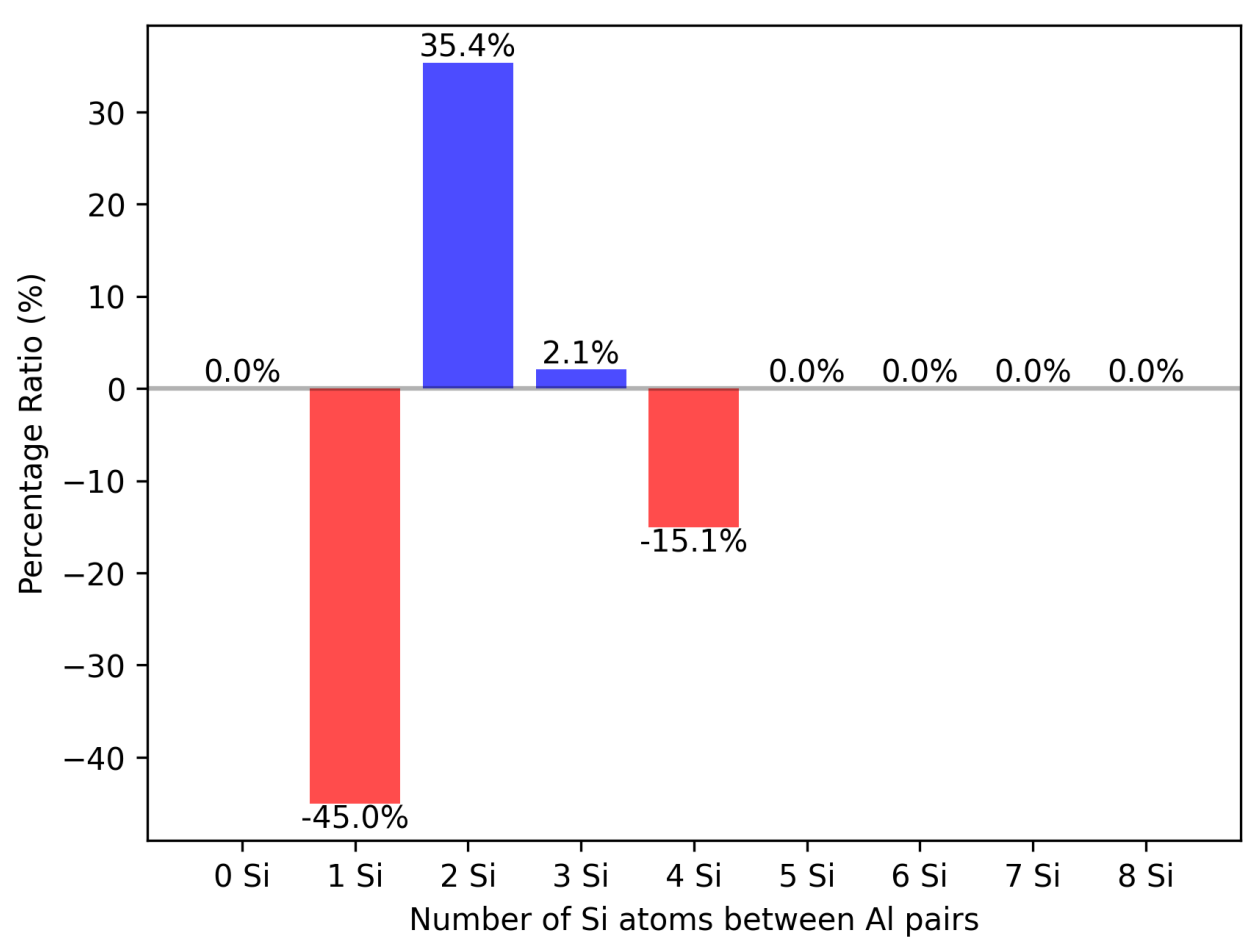

Fig. S7 5 Al CHA 1x1x1 overpopulation analysis. Again a strong preference for Al-Si-Si-Al is seen, and a disincentive to form Al-Si-Al linkages. The higher concentration of Al with a preference for 2 and 3 Si between Al pairs, depopulates the other possible separations.

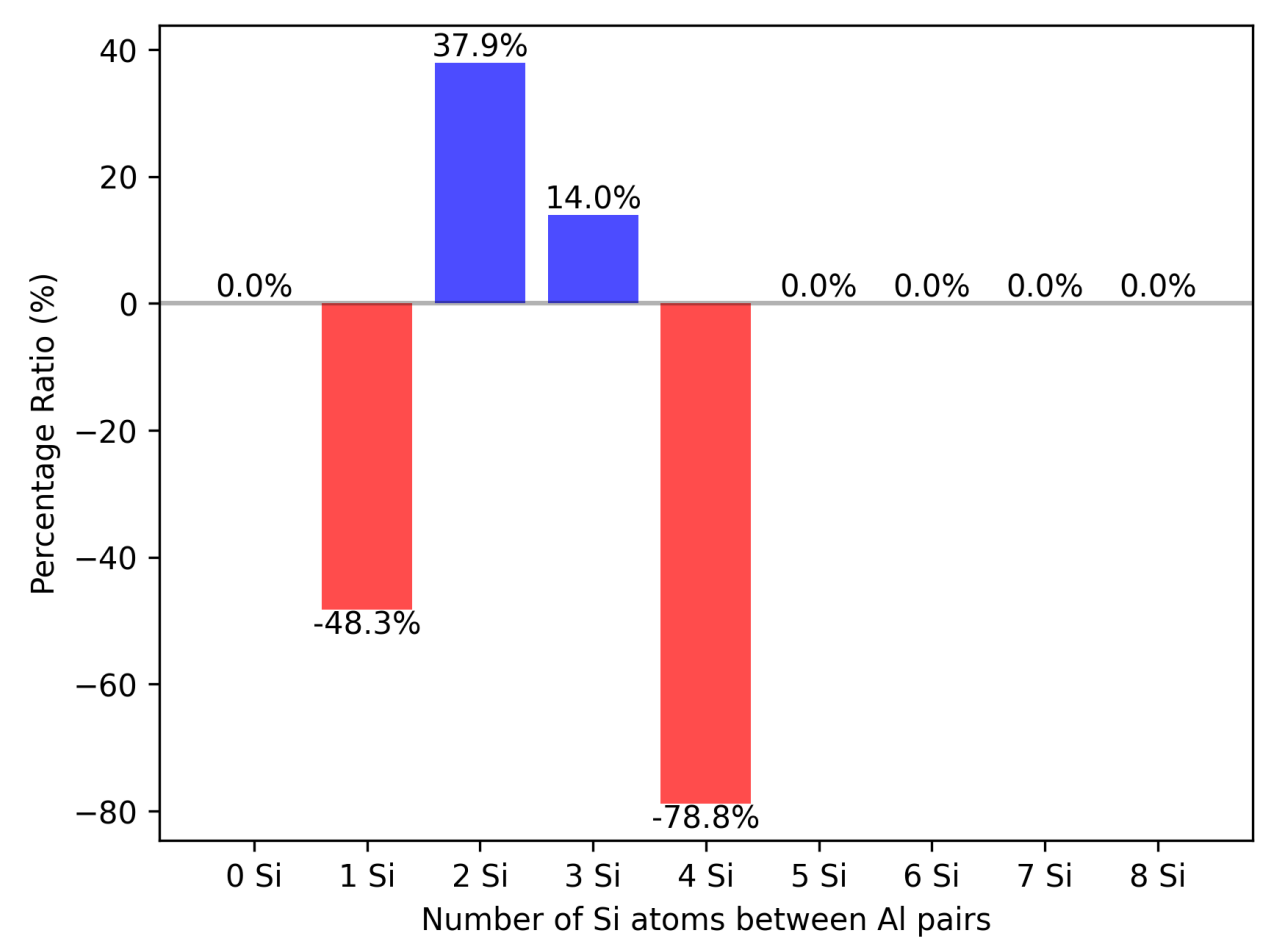

Fig. S8 6 Al CHA 1x1x1 overpopulation analysis. The distribution is somewhat similar to the 5 Al case, except there is a more pronounced underpopulation of cases where Al-Si-Si-Si-Si-Al linkages are seen due to the preference for forming pairs at shorter separations.

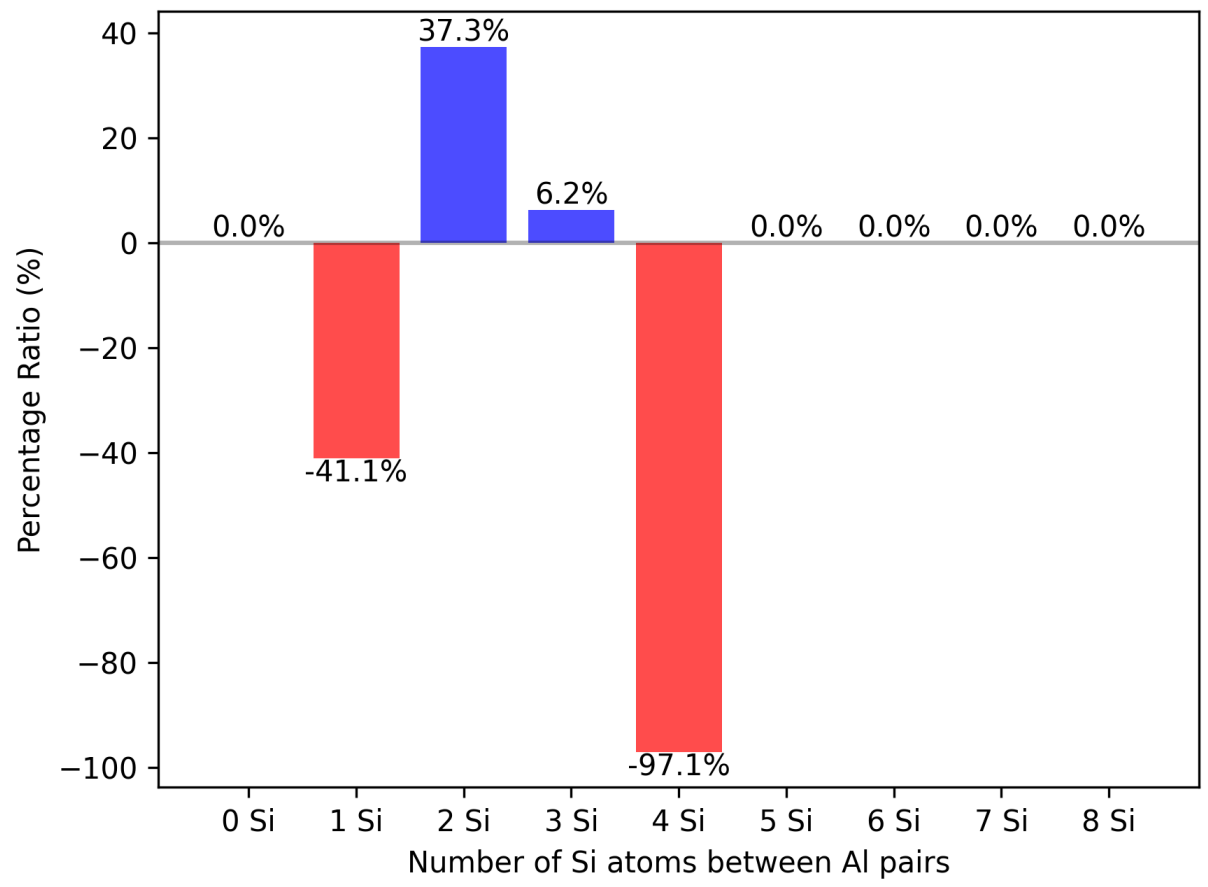

Fig. S9 7 Al CHA 1x1x1 overpopulation analysis. The distribution is again qualitatively similar to the 5 and 6 Al cases except due to the even higher concentration of Al, the Al-Si-Si-Si-Si-Al linkages are more strongly disincentivised.

3.1.2 CHA 1x2x1 overpopulation analysis

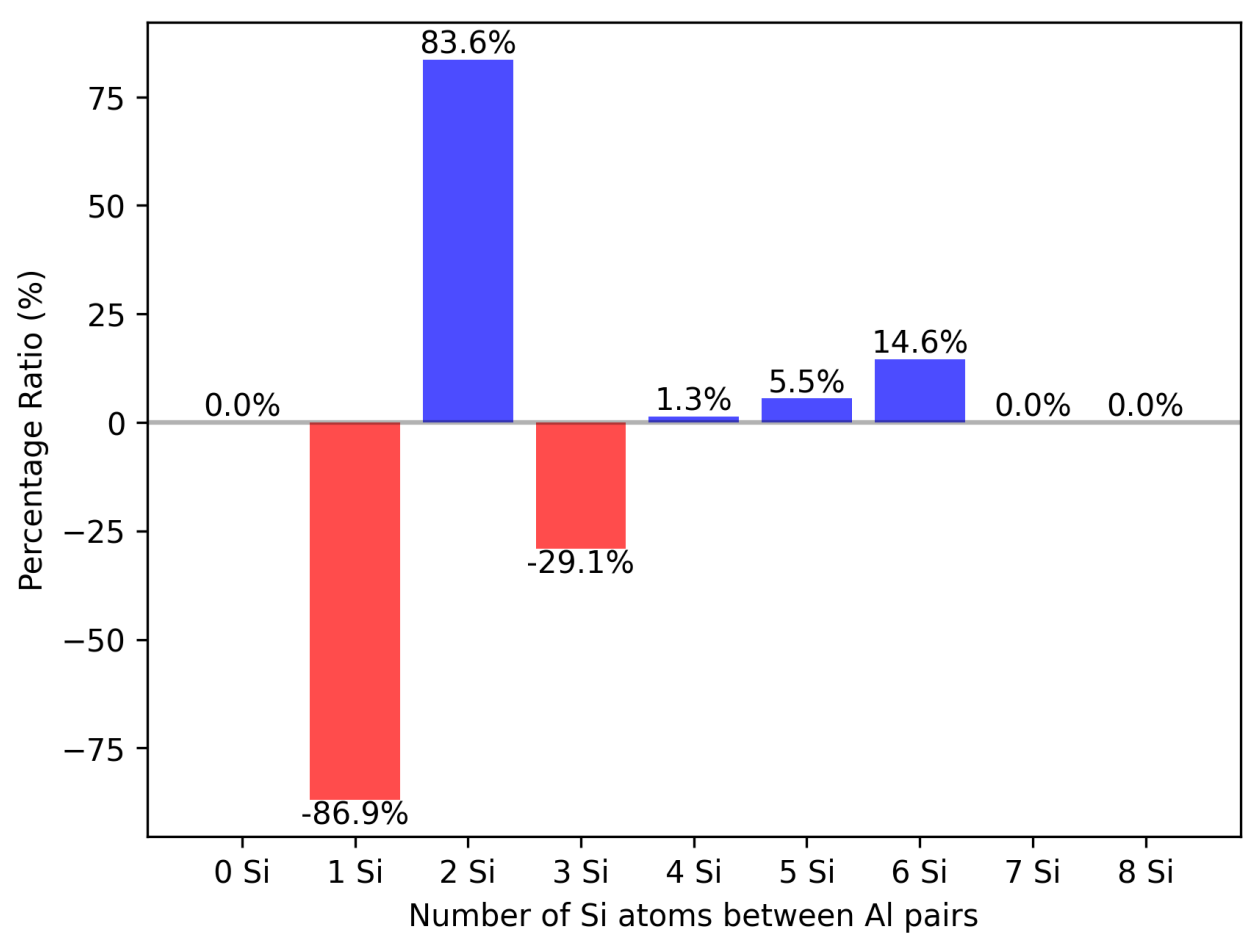

Fig. S10 3 Al CHA 1x2x1 overpopulation analysis. In comparison to the 1x1x1 case, the longer b direction allows for greater separation of Al pairs which means randomly generated structures can contain linkages of Al-Si-Si-Si-Si-Si-Si-Al but consistent with the 1x1x1 results, there is a strong preference for thermodynamically stable structures to adopt Al-Si-Si-Al linkages.

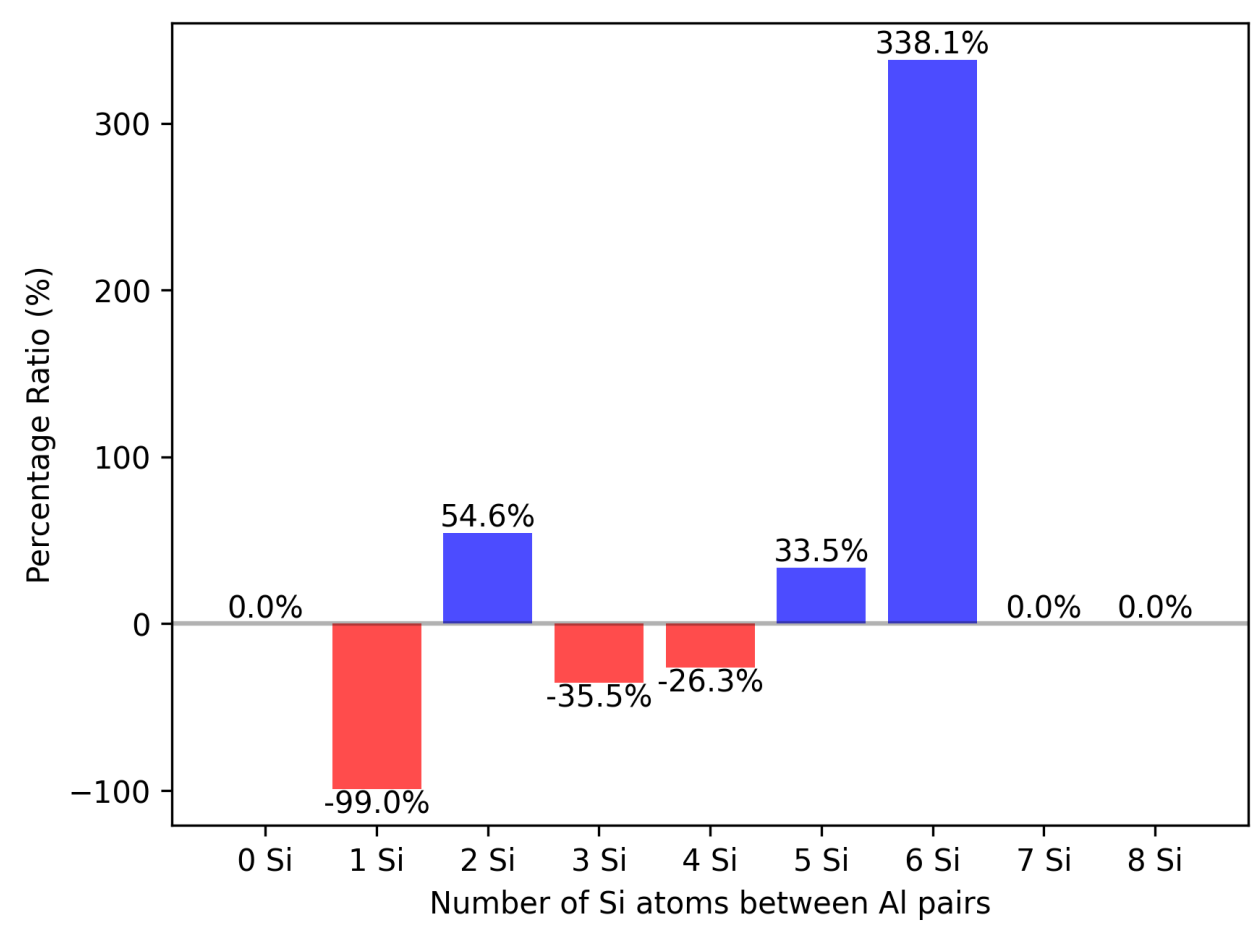

Fig. S11 4 Al CHA 1x2x1 overpopulation analysis. The 4 Al case is quite distinct from the 3 Al case, the two most pronounced peaks being at 2 and 6 Si atoms separating Al pairs. The reason for this slightly peculiar distribution is that in the 1x2x1 structure, the Al pairs in D6Rs, but unlike in the 1x1x1 case, the D6Rs are in independent CHA supercages, and hence separated by a much greater distance.

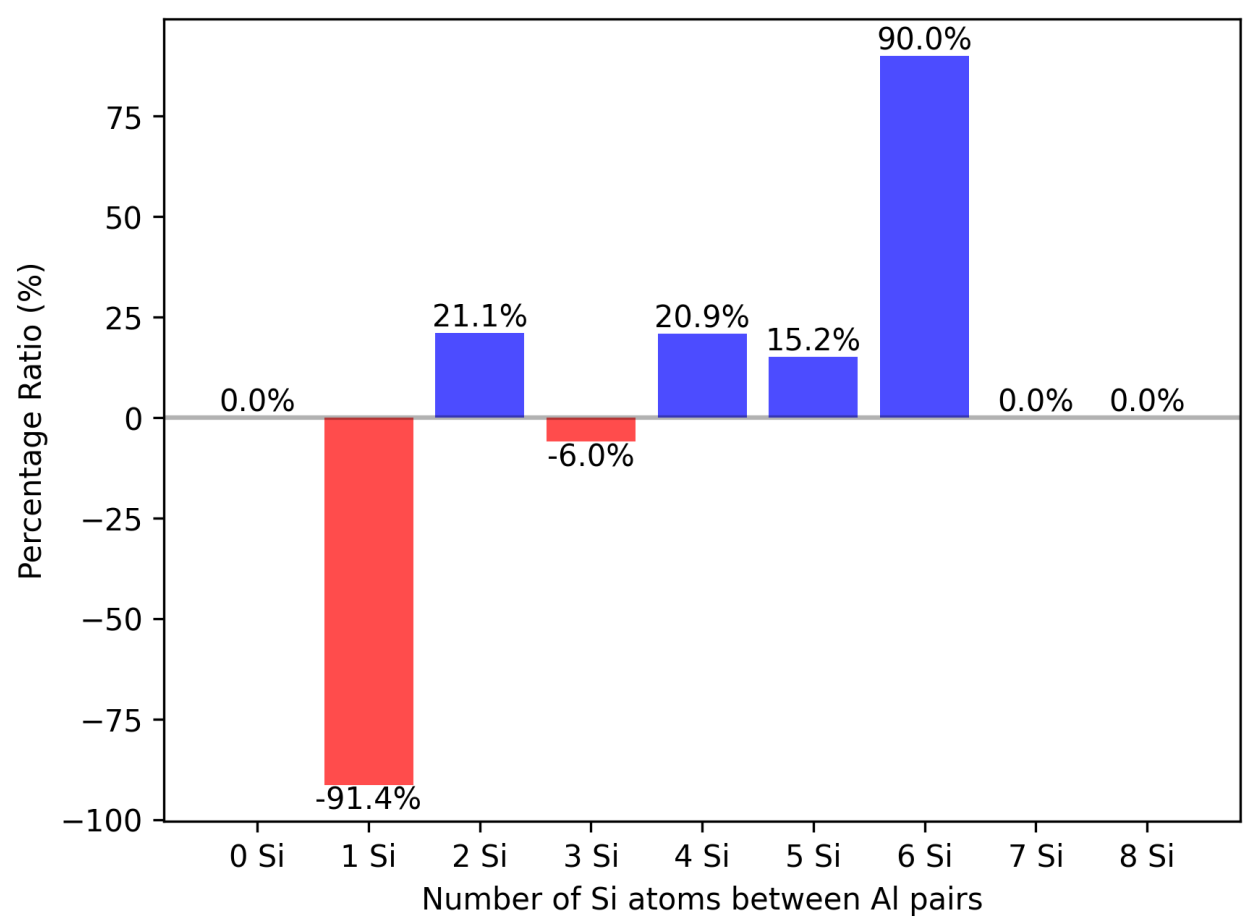

Fig. S12 5 Al CHA 1x2x1 overpopulation analysis. Again, the pronounced disincentive to form Al-Si-Al sequences is evident and preference to form Al-Si-Si-Al linkages.

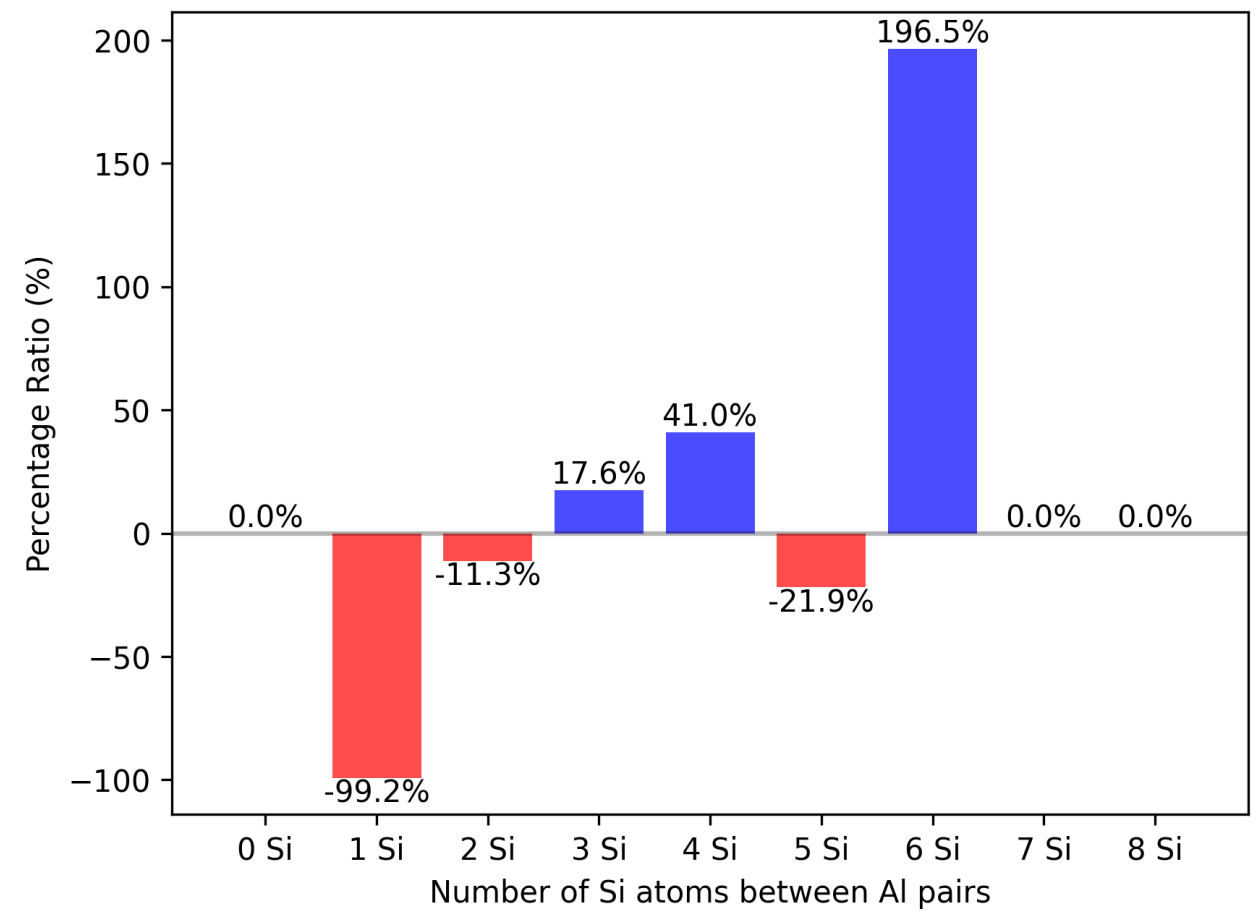

Fig. S13 6 Al CHA 1x2x1 overpopulation analysis. Aside from the disincentive to form Al-Si-Al linkages, the most striking feature of the figure is perhaps the strong overpopulation of Al-Si-Si-Si-Si-Si-Si-Al linkages which is again attributable to the Al forming Al-Si-Si-Al linkages in distinct D6Rs. What the data indicates is that it would appear that thermodynamics tends to favour segregation of these populated D6Rs rather than aggregation, in clear contrast to the 1x1x1 results. This result does highlight the perils of using simulation cells that are too small to allow the adoption of more favourable Al arrangements.

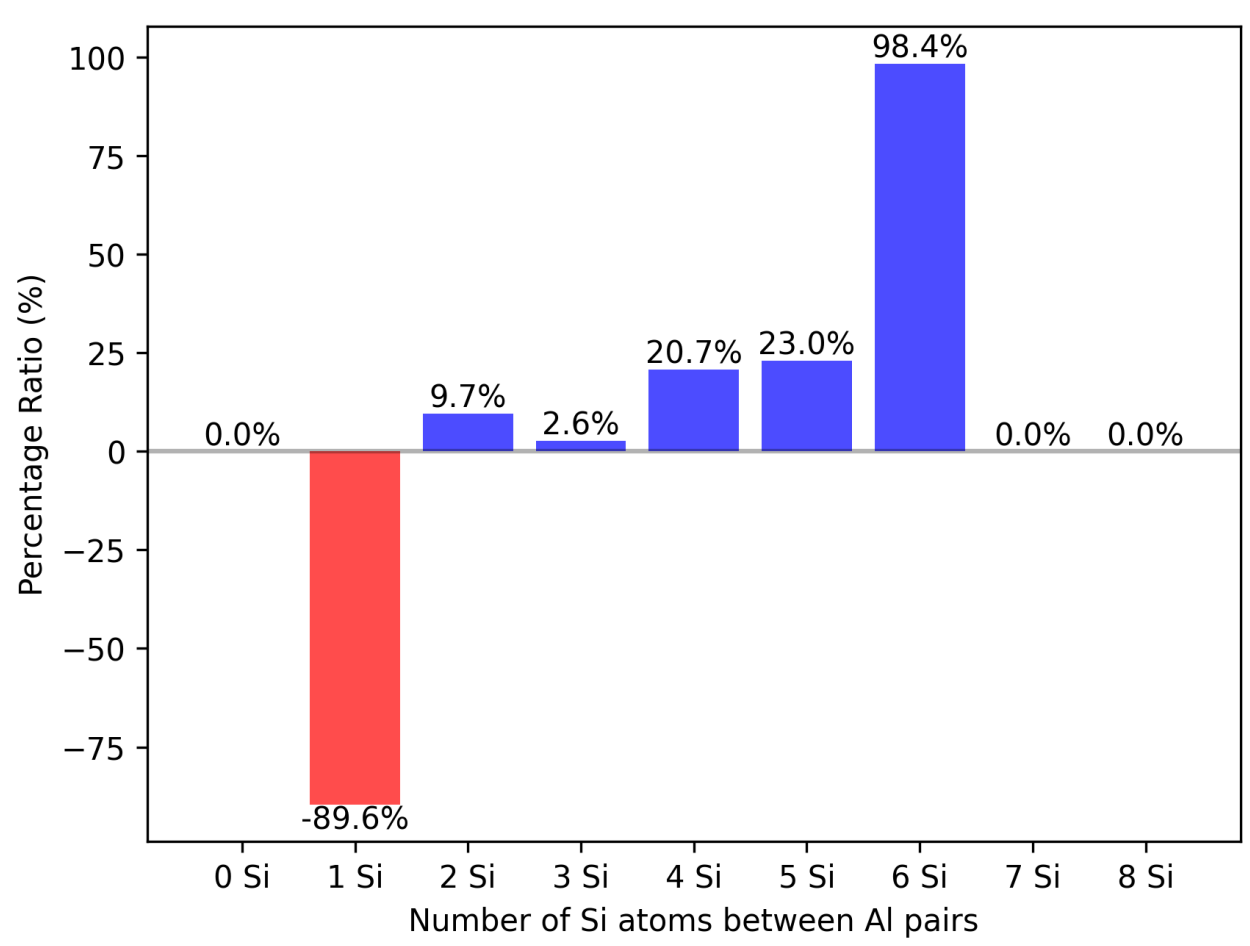

Fig. S14 7 Al CHA 1x2x1 overpopulation analysis. Again, it can be seen there is a very pronounced disincentive to form Al-Si-Al linkages, instead the Al is distributed over a greater number of continuous Si linkages. The enhanced overpopulation of Al-Si-Si-Si-Si-Si-Si-Al linkages points to a tendency for the Al to group in supercages but for the Al to be reasonably well separated.

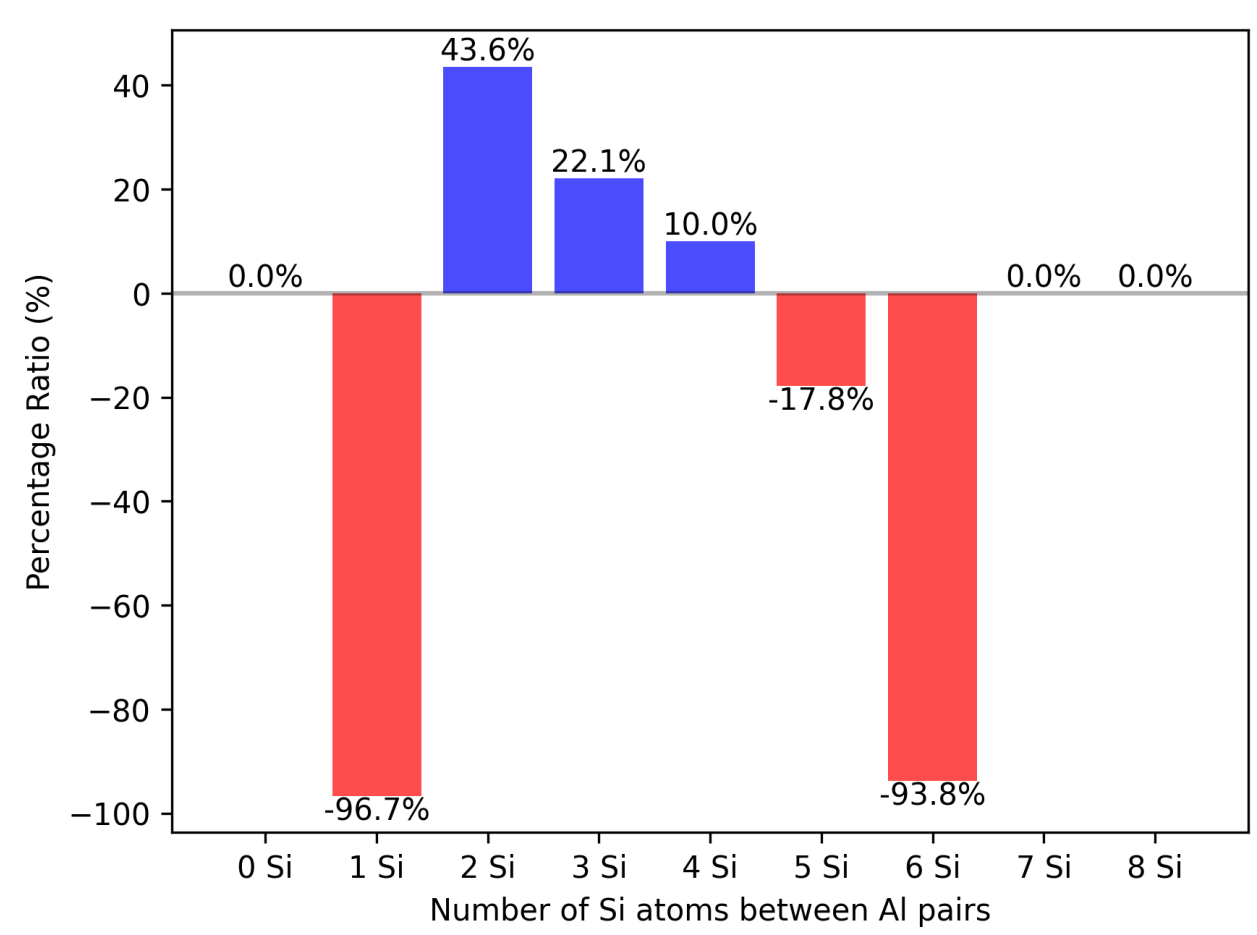

Fig. S15 8 Al CHA 1x2x1 overpopulation analysis. It is interesting to compare this figure with 4 Al CHA 1x1x1 (figure S6), since the concentrations of Al are identical. The distribution over 1-4 Si atoms follows a very similar relative trend with the strong disincentive to form Al-Si-Al, and incentive to form linkages with 2, 3 and 4 Si atoms between Al pairs due to Al pairing within neighbouring D6Rs. This data shows there is tendency for the occupied D6Rs to be in close proximity rather than maximally separated, hence the underpopulation at 5 and 6 Si atoms.

3.1.3 RHO 1x1x1 overpopulation analysis

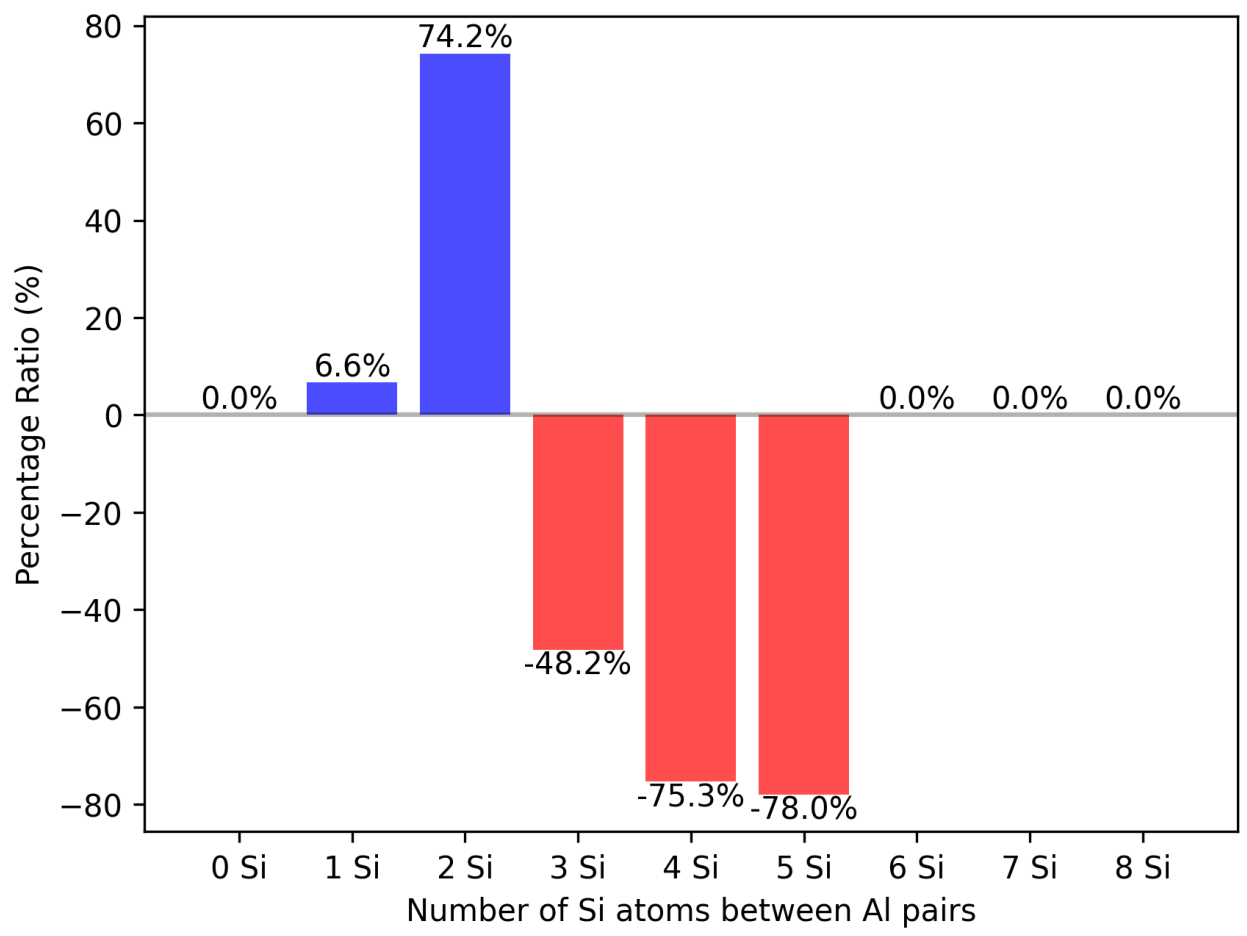

Fig. S16 3 Al RHO 1x1x1 overpopulation analysis. Weak overpopulation of Al-Si-Al pairings in comparison to random structures and a very strong overpopulation of Al-Si-Si-Al. The pronounced under population of Al-Si-Si-Si-Si-Al and Al-Si-Si-Si-Si-Si-Al signals grouping of Al in the single supercage of RHO. Note that as one can see from the cell volumes in table S5, the phase transition resulting in an increase in density has occurred at 3 Al/unit cell (Si:Al ratio = 15), which presumably further enhances the localisation or aggregation of Al.

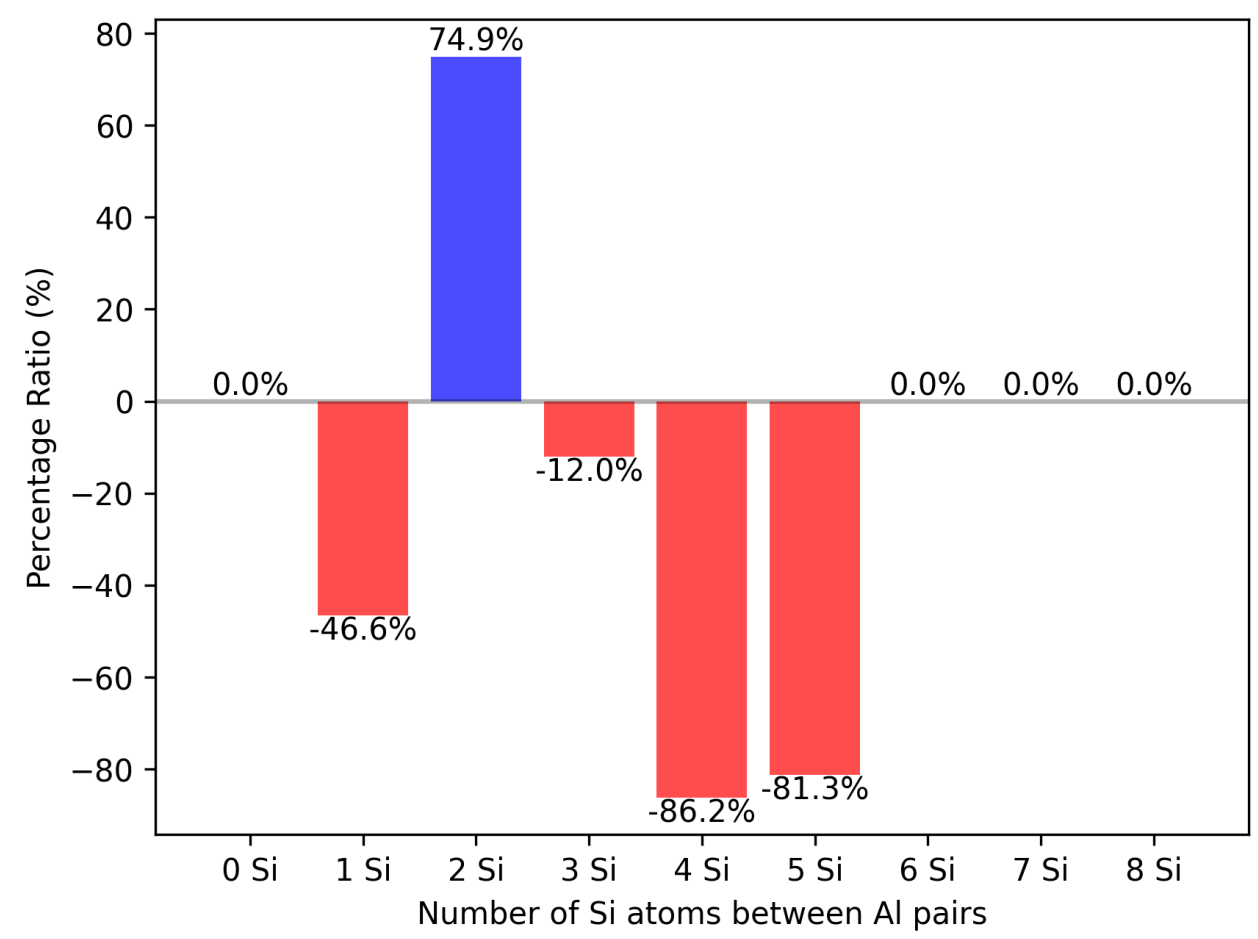

Fig. S17 4 Al RHO 1x1x1 overpopulation analysis. As with figure S16, the pronounced underpopulation at higher separations between Al, indicates a strong preference to aggregate. Interestingly Al-Si-Al linkages are now underpopulated.

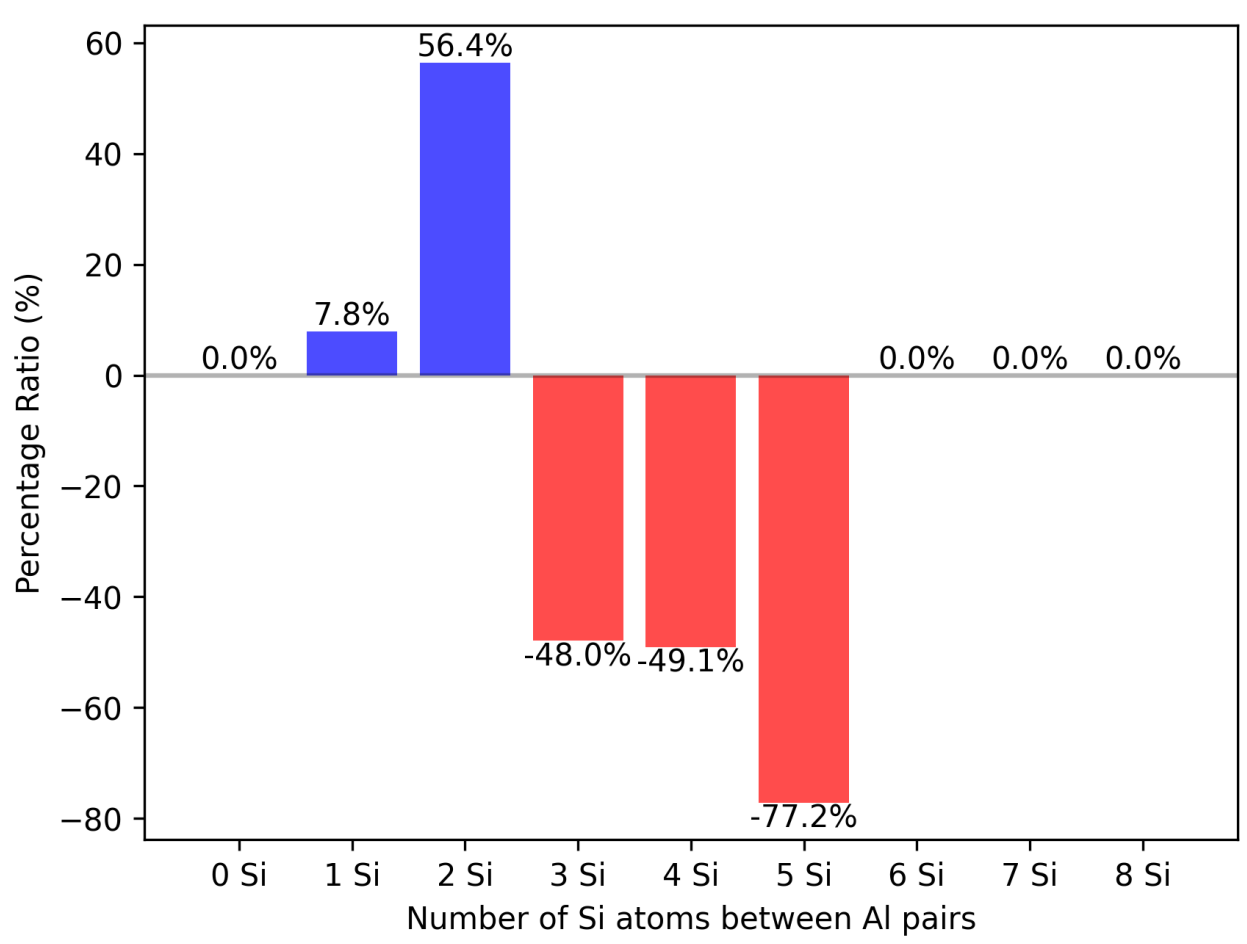

Fig. S18 5 Al RHO 1x1x1 overpopulation analysis. There is now a very minor but in comparison to MOR and CHA, overpopulation of Al-Si-Al and Al-Si-Si-Al pairings. Additionally, a strong depopulation of higher Si Al pair separations is very evident again showing aggregation.

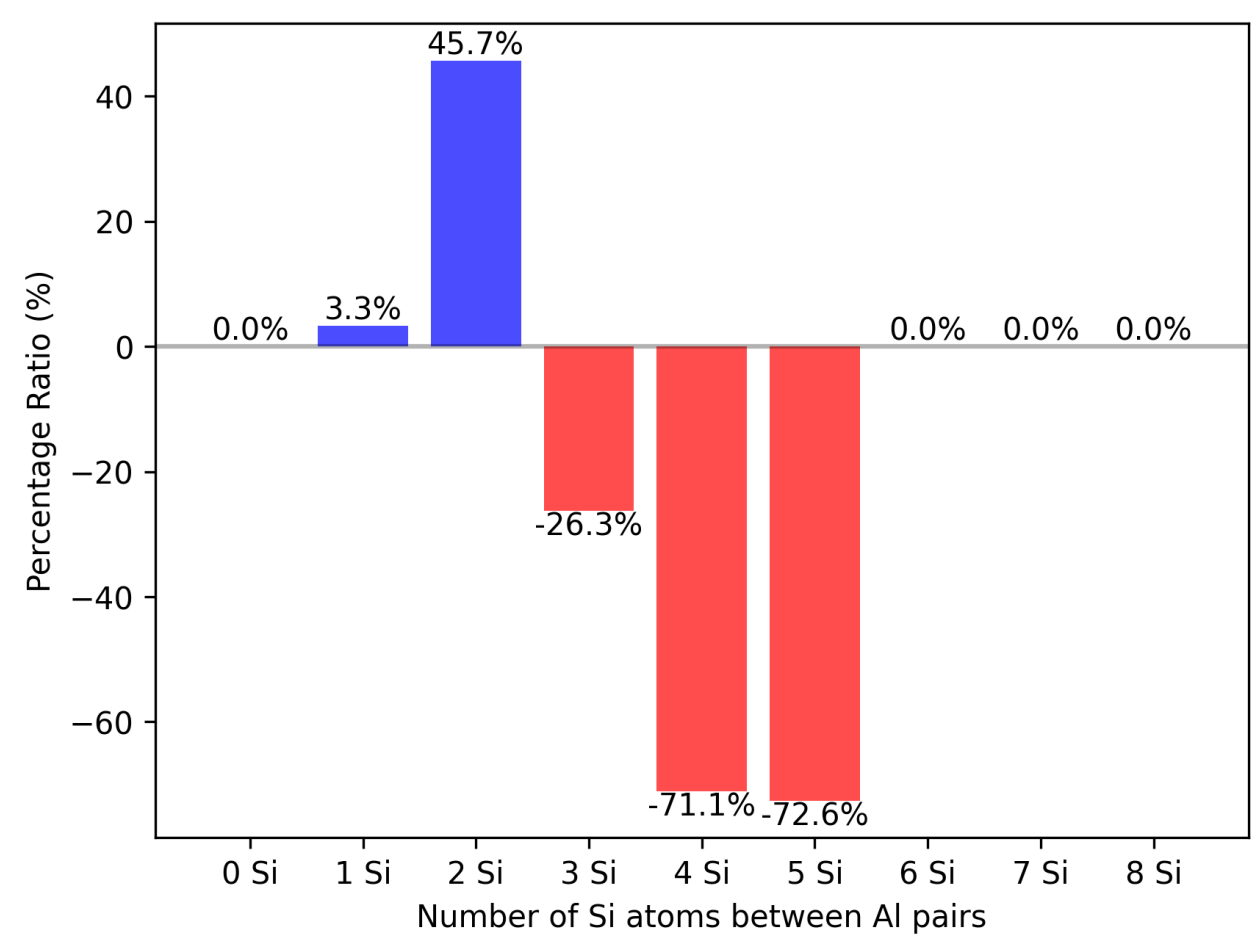

Fig. S19 6 Al RHO 1x1x1 overpopulation analysis. The populations are very similar to figure S18 with a pronounced overpopulation at low Si separations between Al pairs and underpopulation at high Si separations between Al pairs.

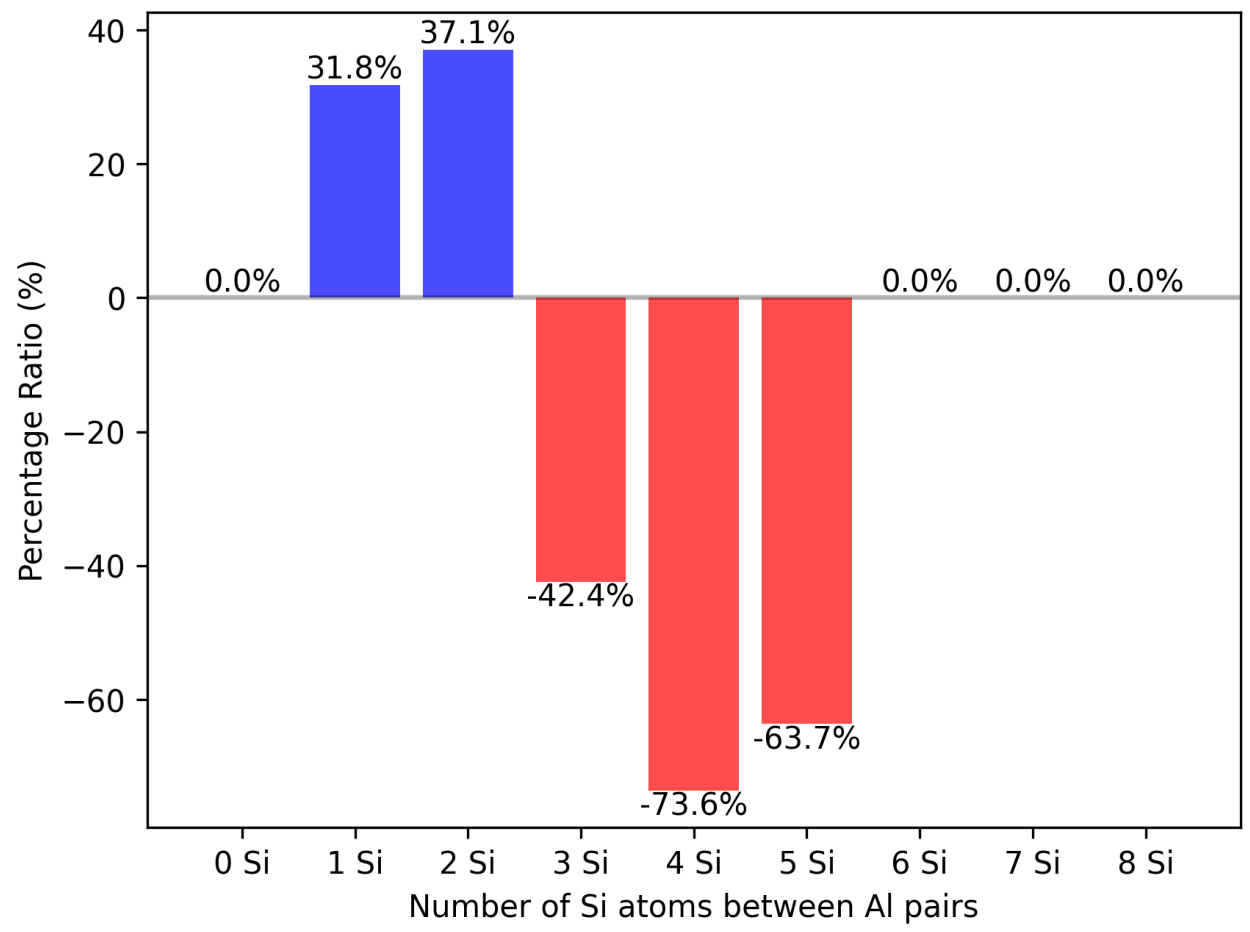

Fig. S20 7 Al RHO 1x1x1 overpopulation analysis. There is a surprising consistency in the distributions for 6 and 7 Al atoms/per cell. This appears to be driven by pairing at shorter distances.

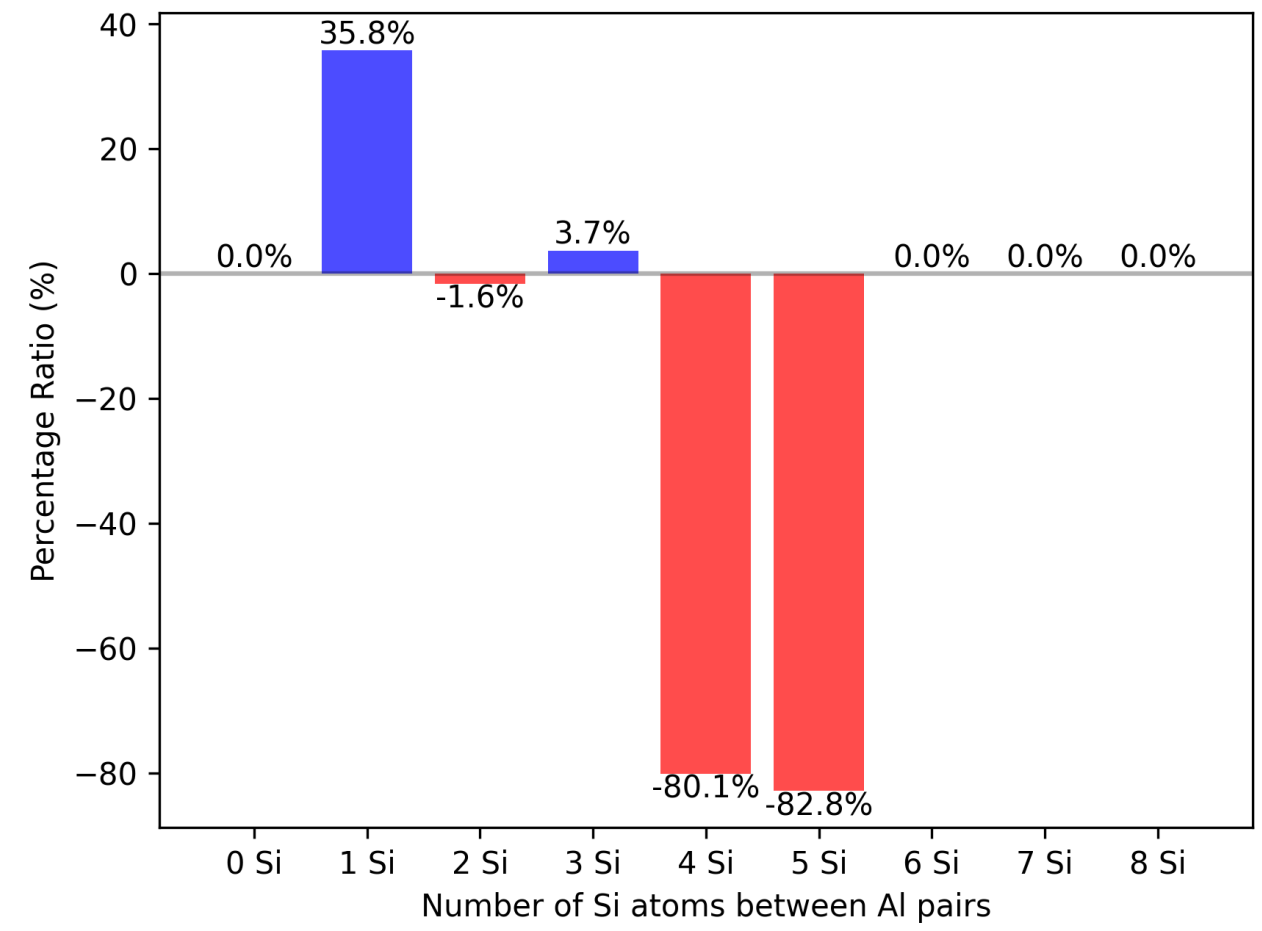

Fig. S21 8 Al RHO 1x1x1 overpopulation analysis, consistent with strong overpopulation of Al-Si-Al.

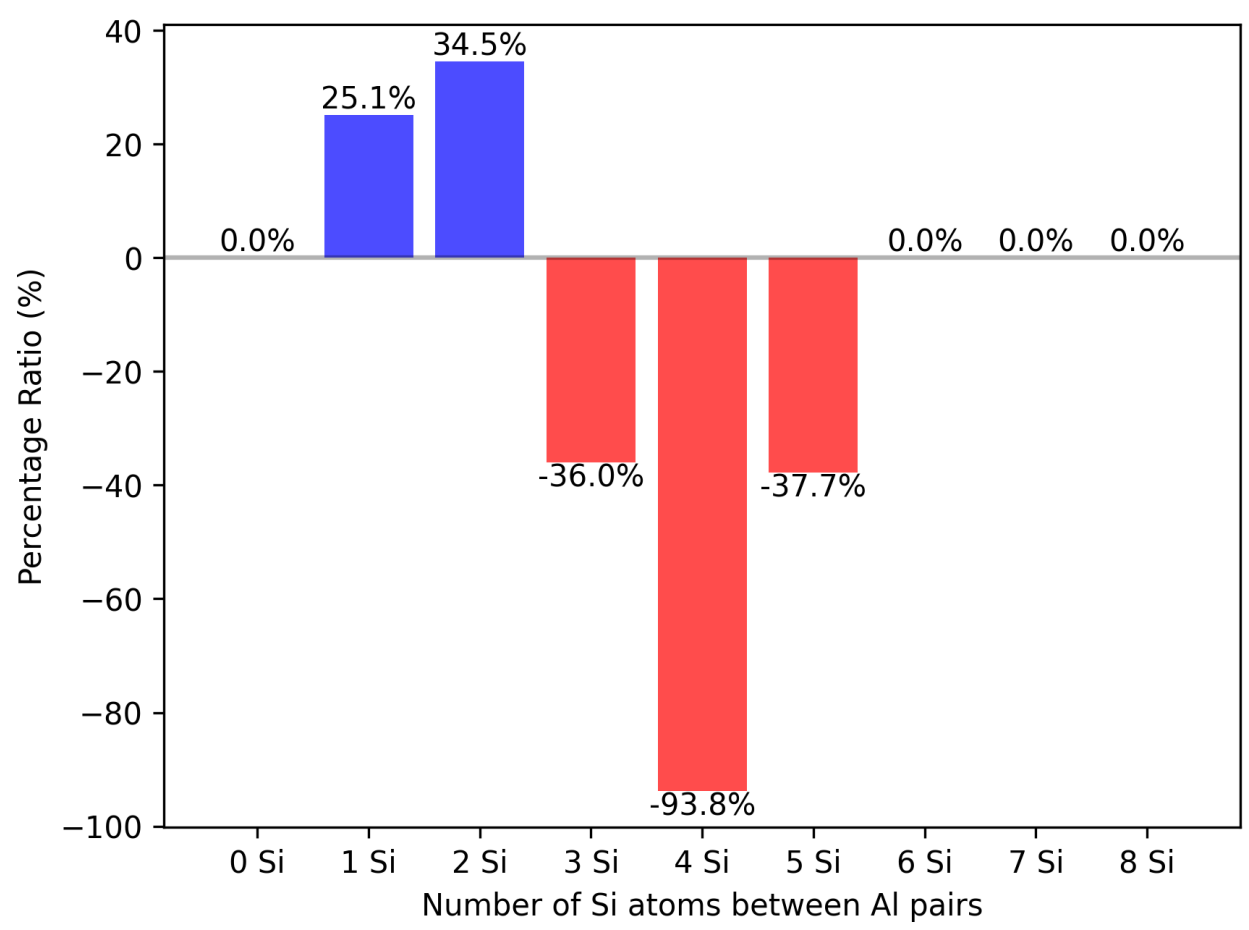

Fig. S22 9 Al RHO 1x1x1 overpopulation analysis. For figure 9 and 10 Al per unit cell, the profiles are very similar. The most striking feature which commensurate with strong aggregation is the pronounced overpopulation of Al-Si-Al pairings in marked contrast to both MOR and CHA.

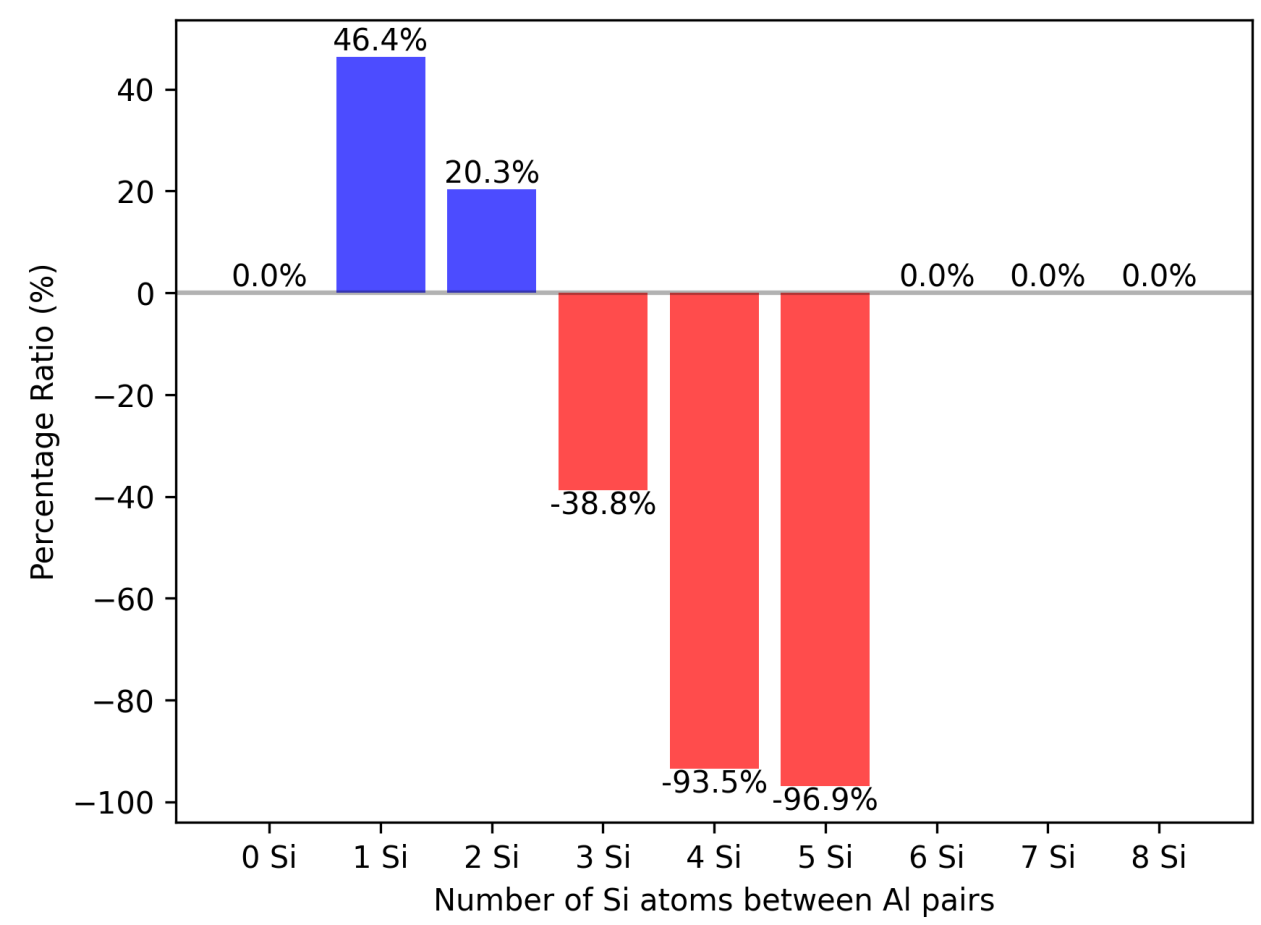

Fig. S23 10 Al RHO 1x1x1 overpopulation analysis that shows the strong overpopulation associated with aggregation in and surrounding the RHO supercage, observed through the Al-Si-Al and Al-Si-Si-Al pairings.

3.1.4 MOR 1x1x2 overpopulation analysis

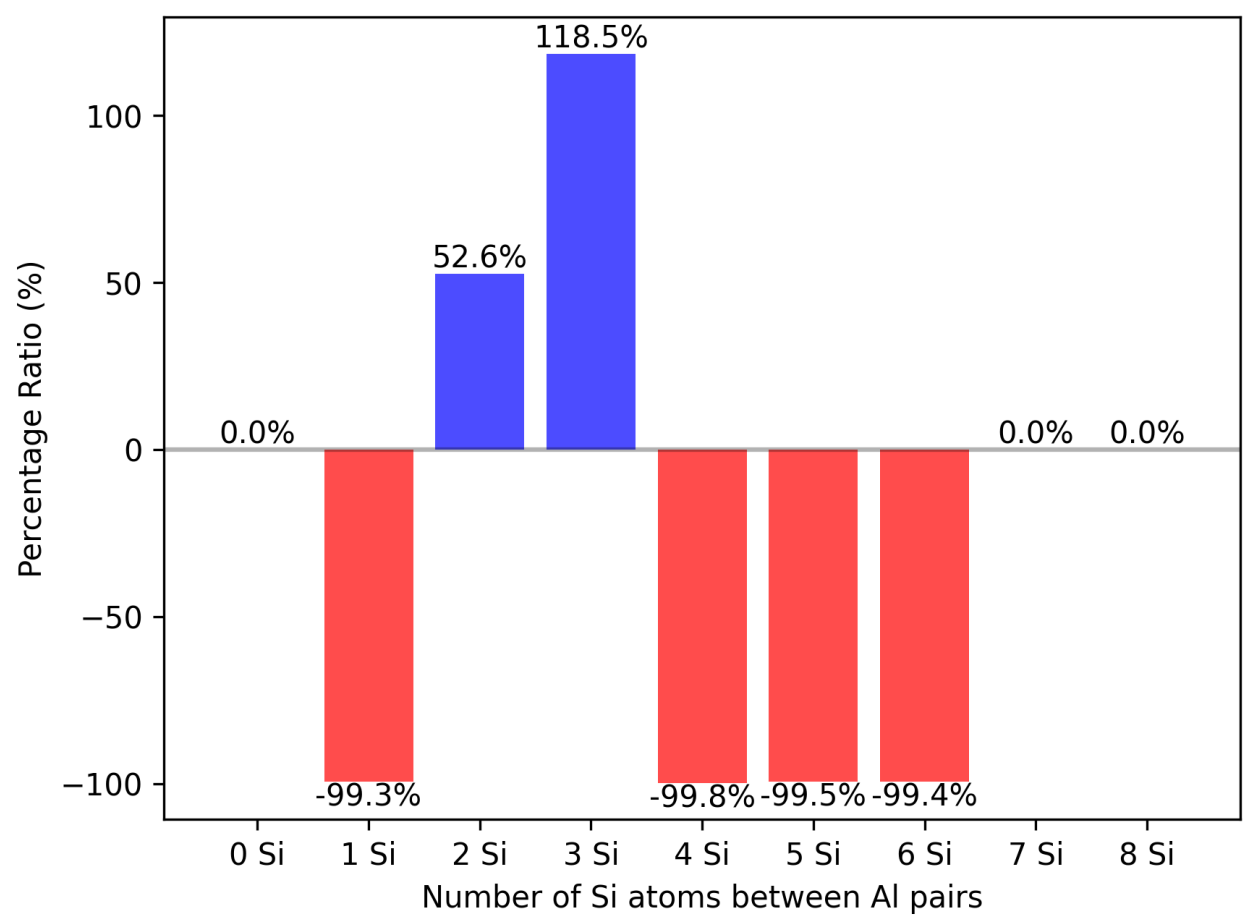

Fig. S24 3 Al MOR 1x1x2 overpopulation analysis. Similar to CHA at this high Si:Al ratio, there is a strong underpopulation of Al-Si-Al pairings, overpopulation of Al-Si-Si-Al pairings and underpopulation of higher Si separations between pairings.

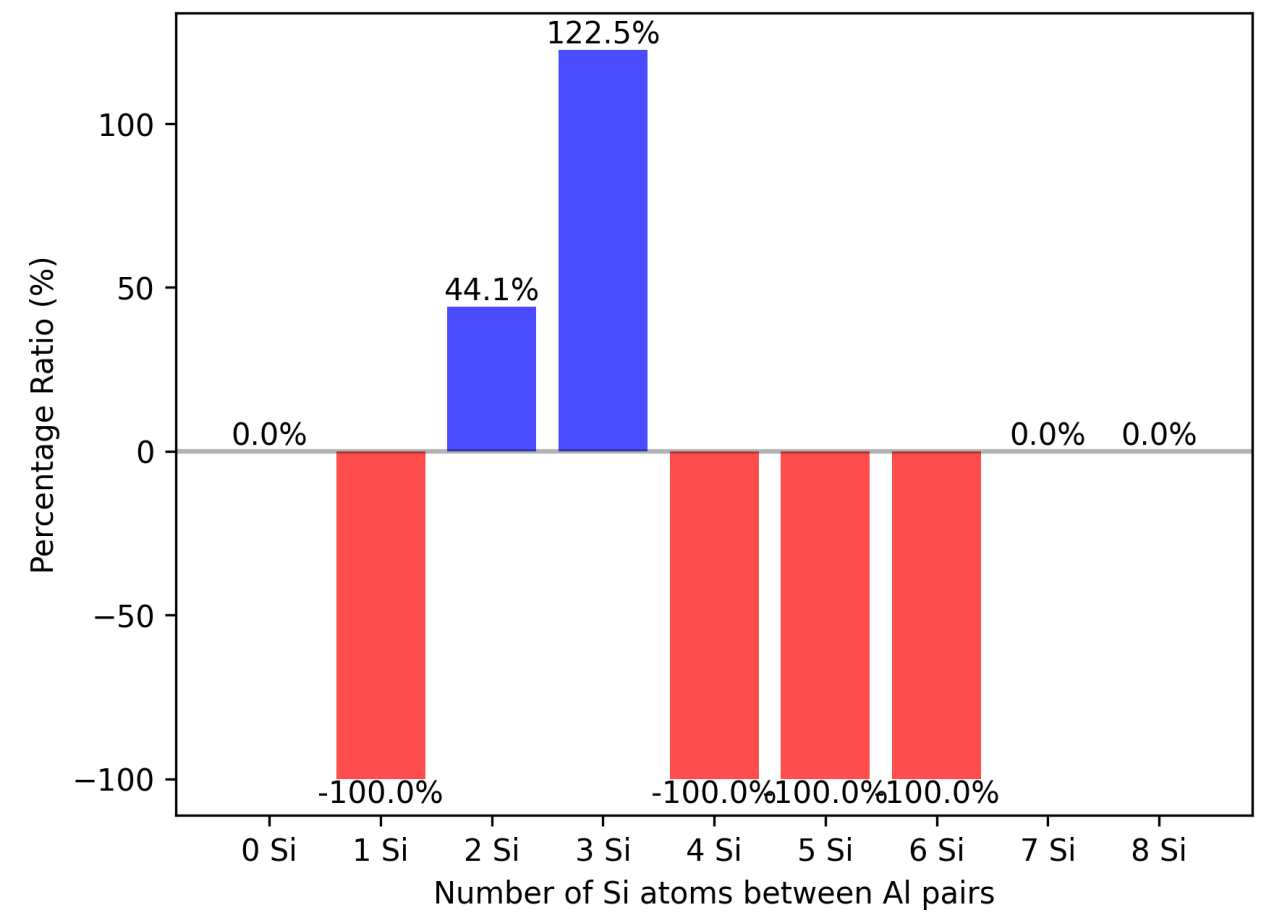

Fig. S25 4 Al MOR 1x1x2 overpopulation analysis. There is strong underpopulation of Al-Si-Al, Al-Si-Si-Si-Si-Al, Al-Si-Si-Si-Si-Si-Al and Al-Si-Si-Si-Si-Si-Si-Al and overpopulation of Al-Si-Si-Al and Al-Si-Si-Si-Al pairings. This is explicable as there systematic occupation of a single channel in MOR.

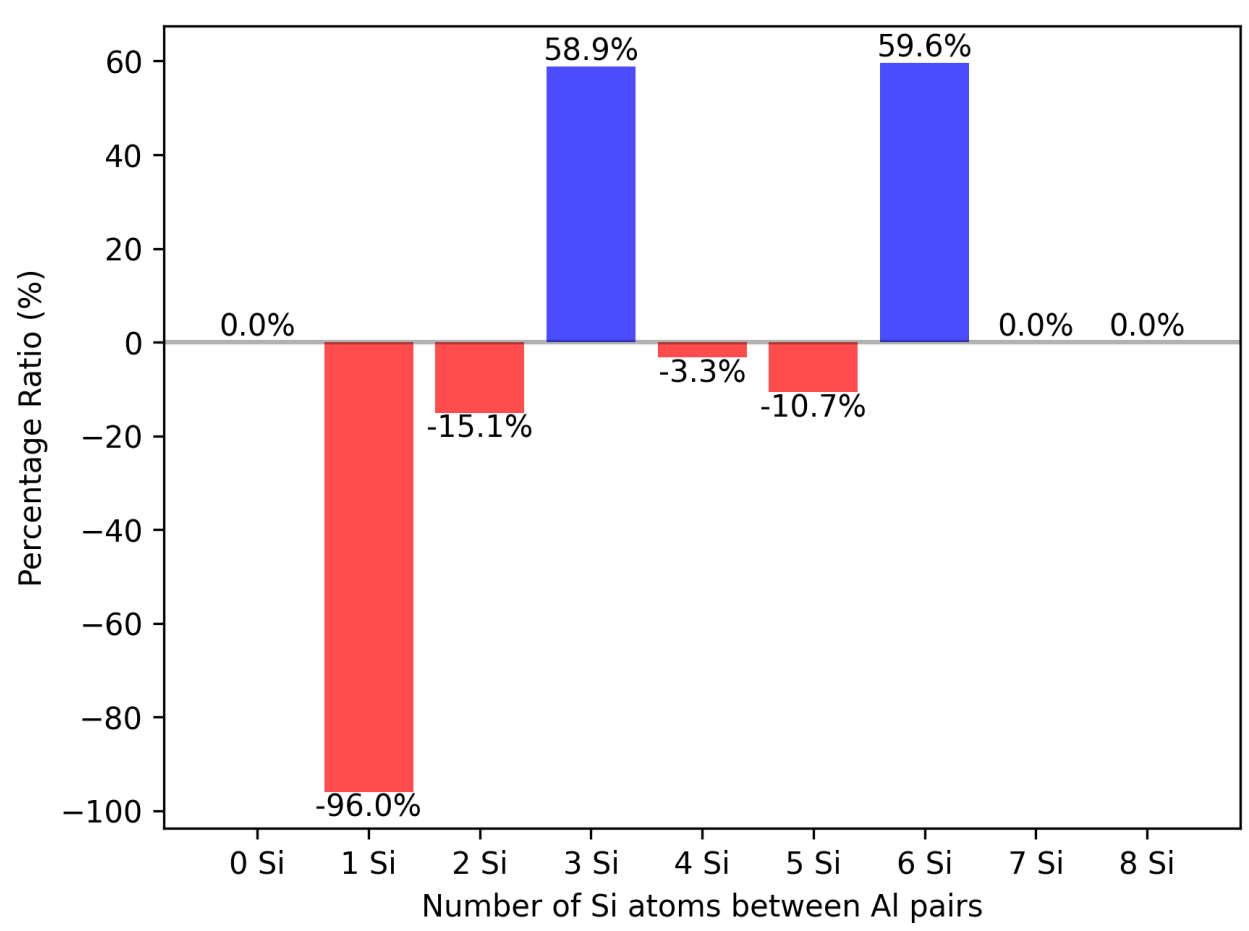

Fig. S26 5 Al MOR 1x1x2 overpopulation analysis. At this Si:Al ratio, one of the channels in the [001] direction is saturated and hence a new channel in the [001] direction is populated. Hence, there is a strong deviation from the previous pattern of pairing and in general terms, since the channels are well separated from one another, there is an underpopulation of short pairings between Al and an overpopulation of larger separations between Al atoms.

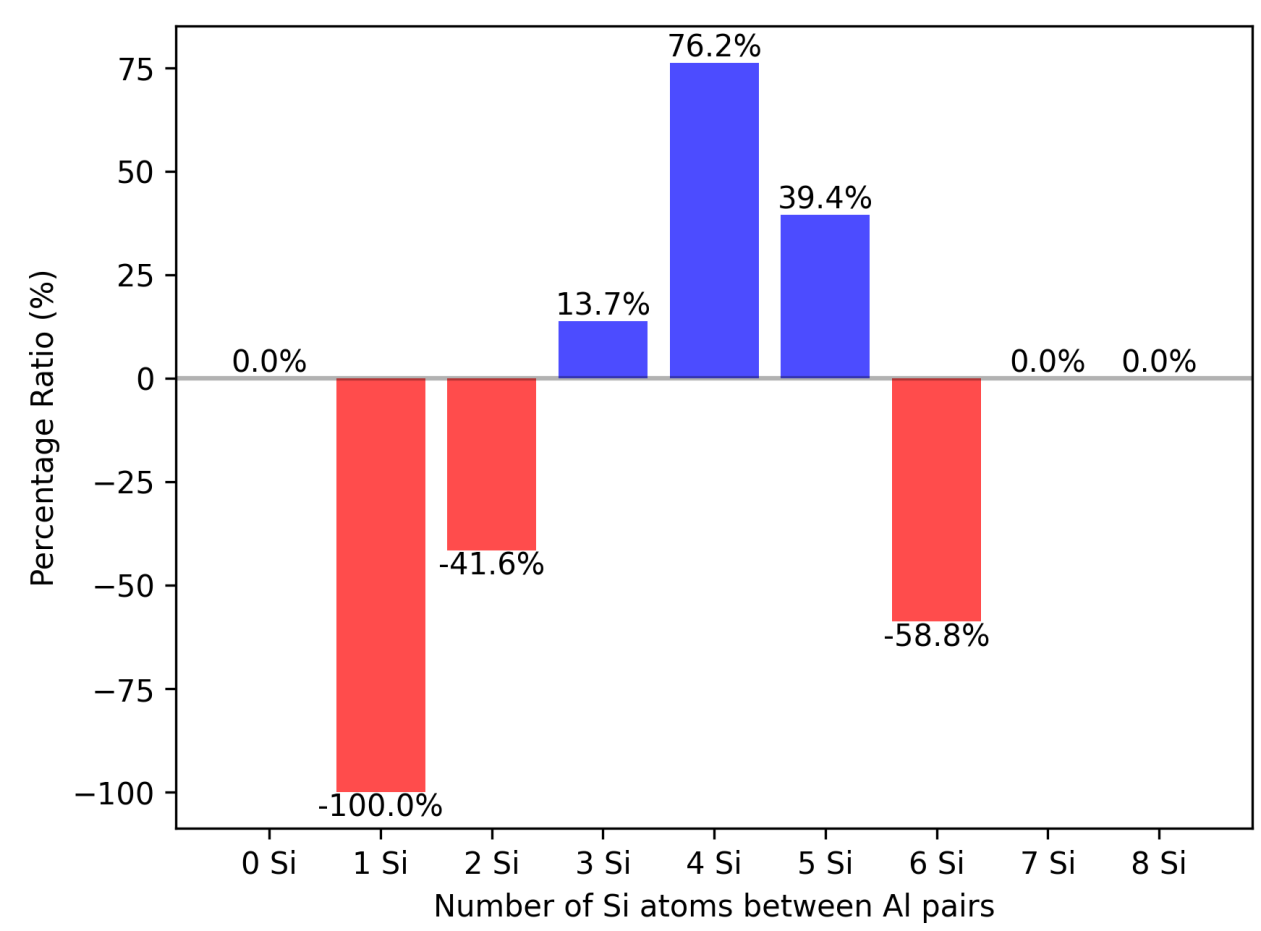

Fig. S27 6 Al MOR 1x1x2 overpopulation analysis. Very similar to figure S26. Two channels parallel to [001] are populated so along the channels there is strong pairing but the channels are well separated, hence the overpopulation at higher Si chain separations.

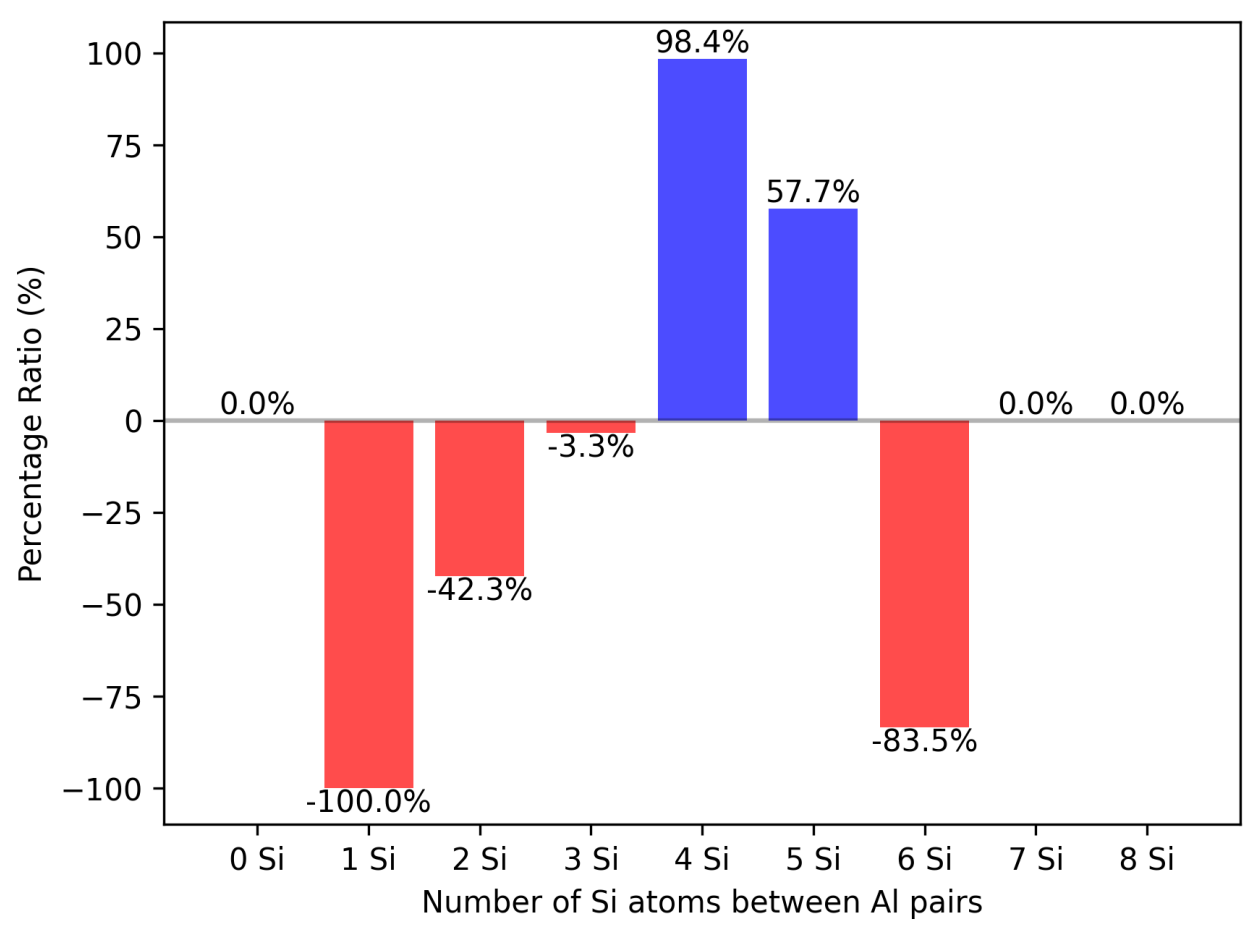

Fig. S28 7 Al MOR 1x1x2 overpopulation analysis. Very similar to 5 and 6 Al. Again, two channels parallel to [001] are populated so along the channels there is strong pairing but the channels are well separated, hence the overpopulation at higher Si chain separations.

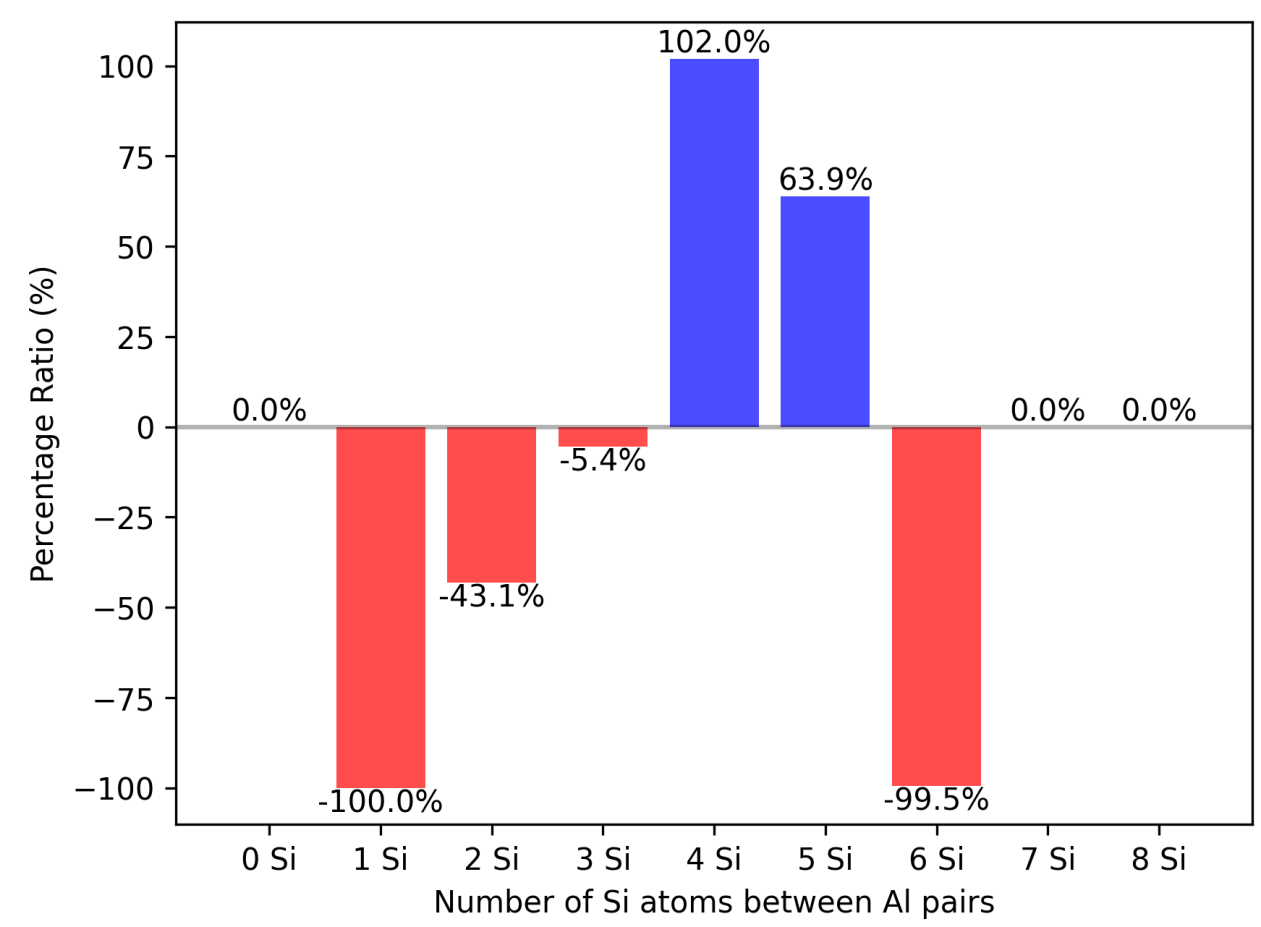

Fig. S29 8 Al MOR 1x1x2 overpopulation analysis. There is a shift with respect to higher Si:Al ratios; two channels along [001] are fully populated but well separated leading to the overpopulation at 4 and 5 Si atoms separating Al pairs.

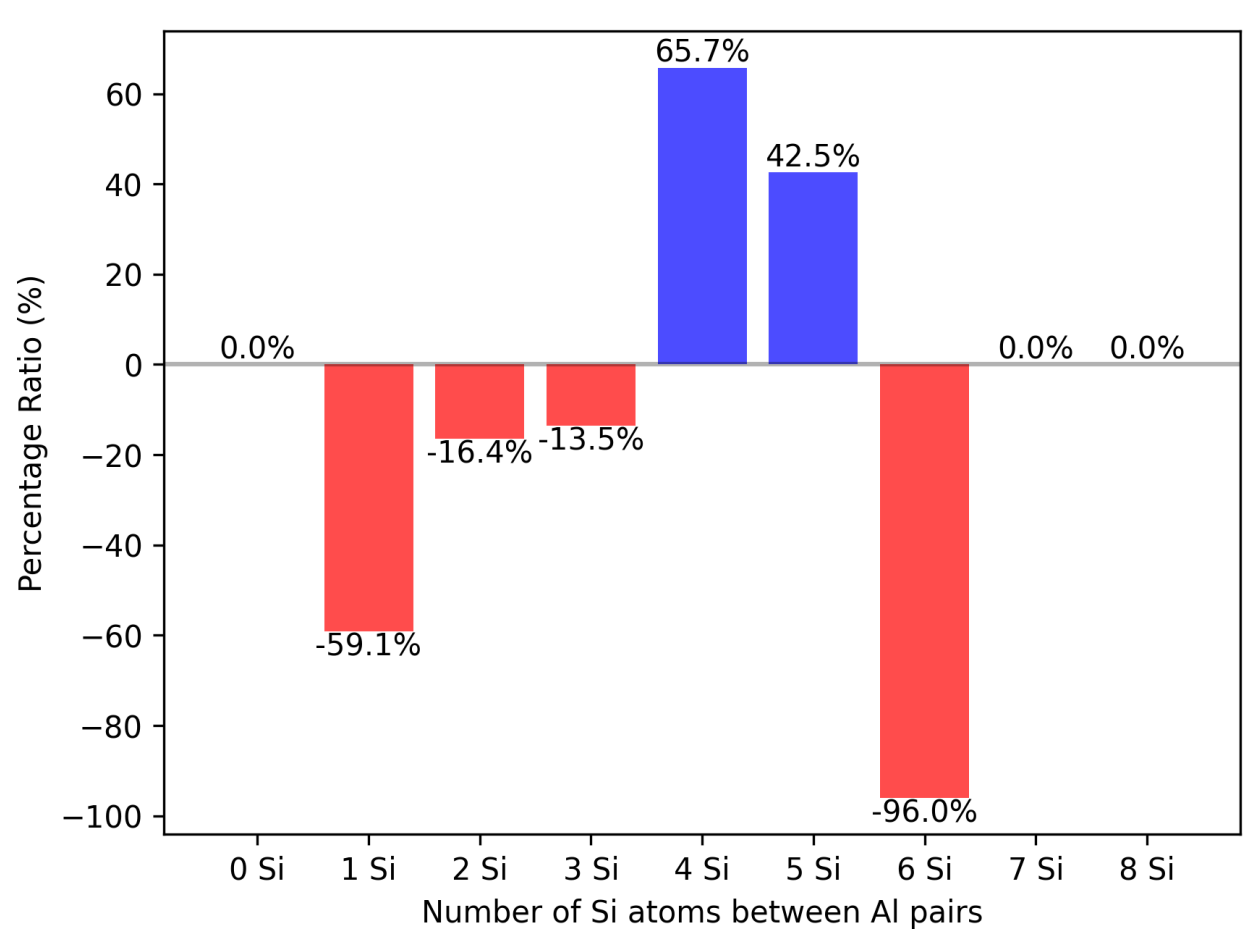

Fig. S30 9 Al MOR 1x1x2 overpopulation analysis. A new channel is populated parallel to [001] but neighbouring another populated channel hence there is net reduction of the population at higher separations of Si atoms between Al pairings.

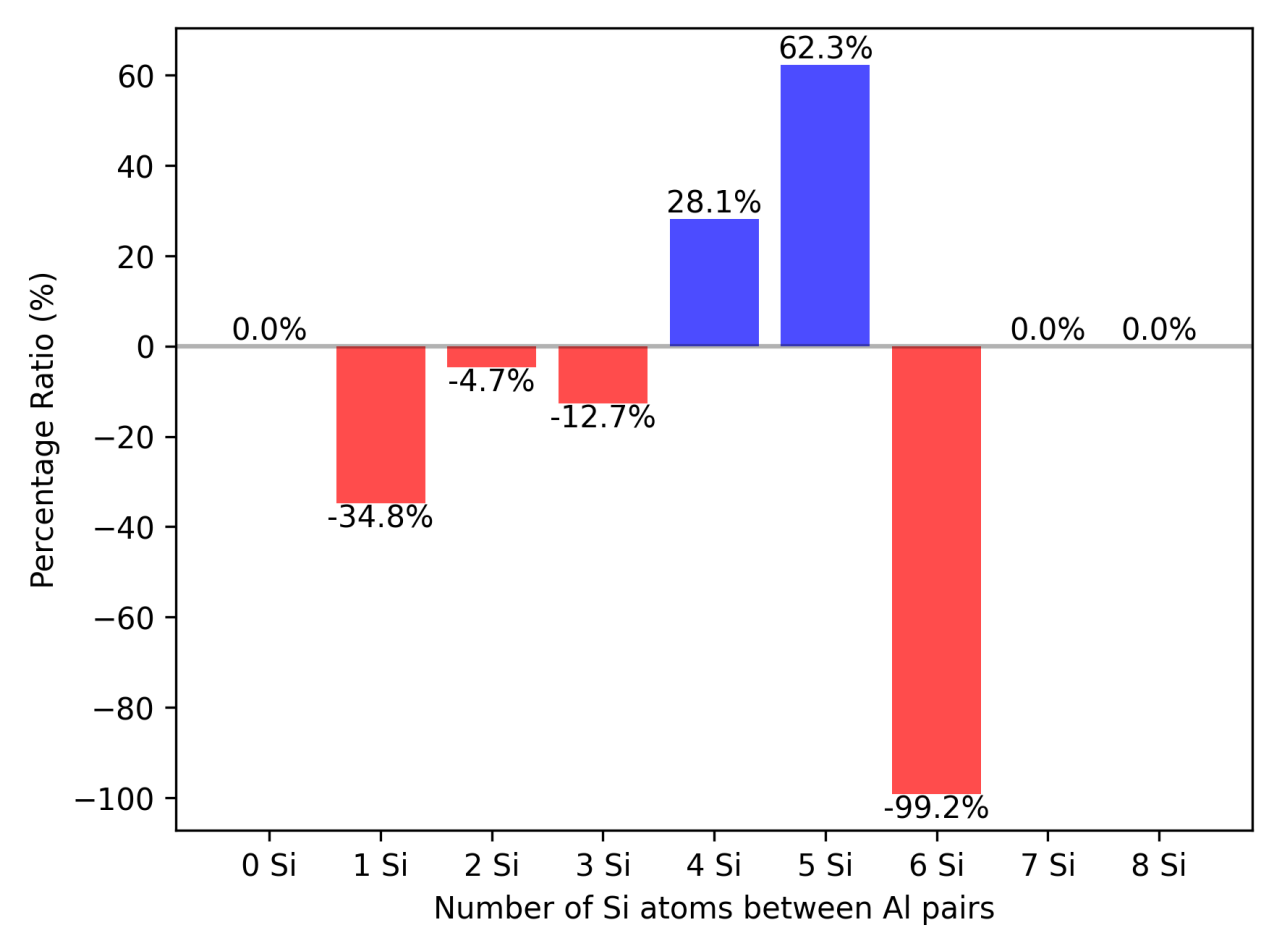

Fig. S31 10 Al MOR 1x1x2 overpopulation analysis. Very similar to figure S30 except there is now population of a 12MR. Interestingly even at this relatively high concentration of Al, there is an under-population at low separations of Al but the Al-Si-Si-Al is preferred relative Al-Si-Al and Al-Si-Si-Si-Al.

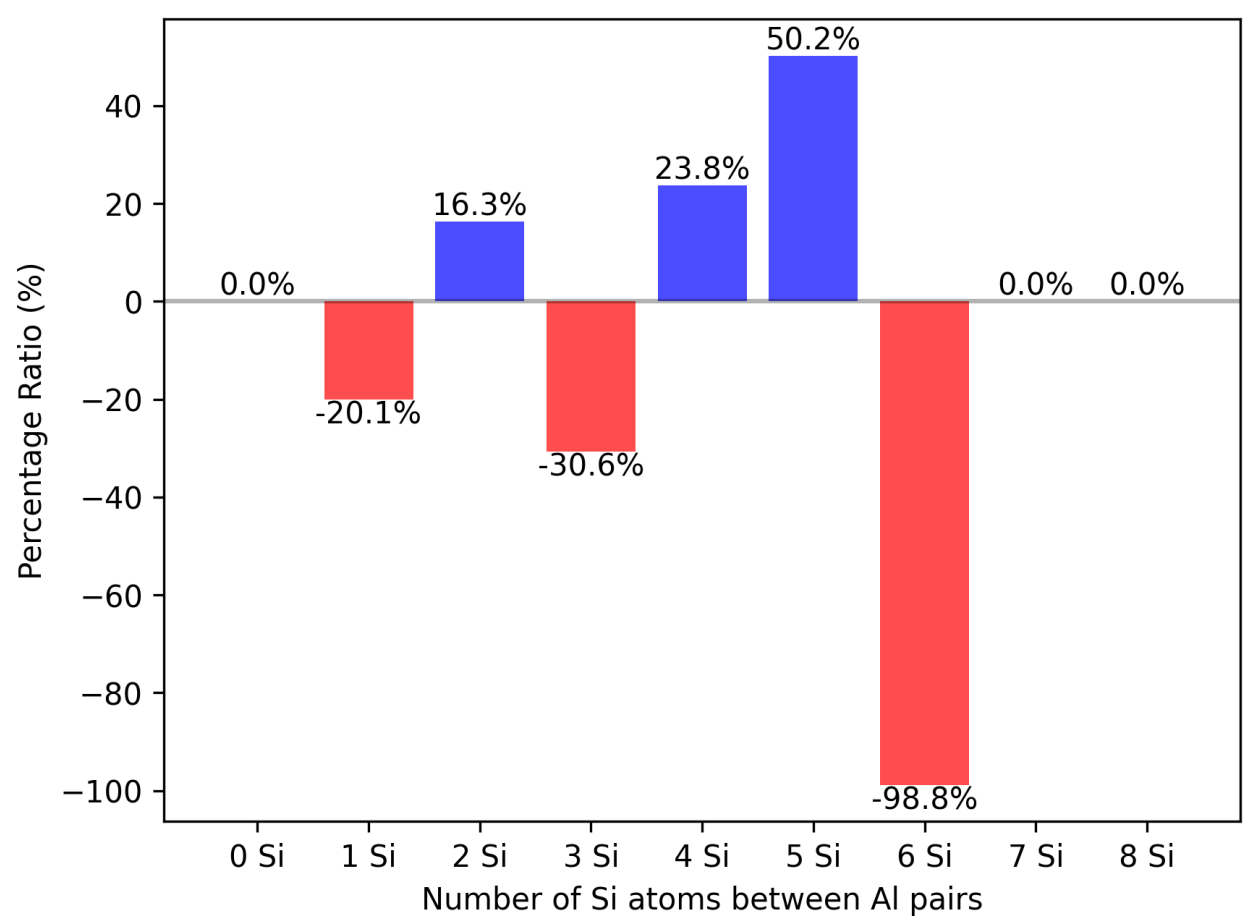

Fig. S32 11 Al MOR 1x1x2 overpopulation analysis. Similar to figure S31 but with an enhanced population of Al-Si-Si-Al pairings due to additional population of a 12MR channel parallel to [001].

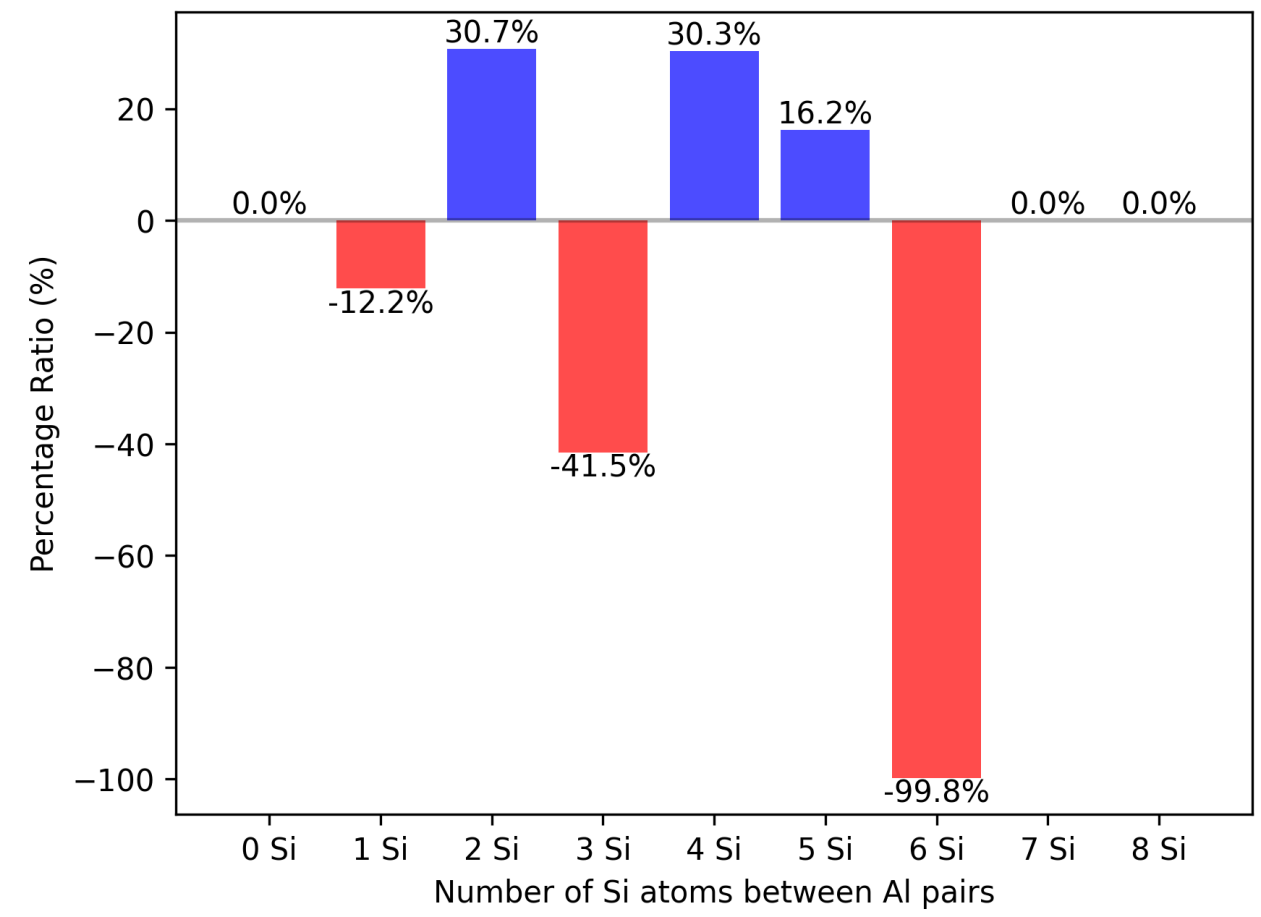

Fig. S33 12 Al MOR 1x2x1 overpopulation analysis. At this concentration there is population of a second 12MR parallel to [001] and there is a preference for Al-Si-Si-Al, Al-Si-Si-Si-Si-Al and Al-Si-Si-Si-Si-Si-Al due to the reasonably well separated channels all populated along the [001] direction i.e. an orientational preference.

4 CP2K settings

All DFT calculations were performed in CP2K[2] using Gaussian basis sets and auxiliary plane wave approach within the Quickstep method[3]. TZV2P-MOLOPT-PBE was the basis set of choice using a cutoff of 1400 Ry, GTH pseudopotentials were also used to model the core electrons. Structures were geometry optimised using the LBFGS[4] algorithm with a force tolerance of 1E-4, cell optimisation was performed with the BFGS approach at a pressure tolerance of 10 bar. All functionals were supplemented with the well validated Grimme "D3" dispersion correction to model the van der Waals interactions[5]. For the PBE0 functional, the Hartree-Fock term was calculated using the ADMM (auxiliary density matrix method)[6] approach with 25% Hartree-Fock exchange.

5 Implementation of Basin Hopping

The algorithm was developed in Python, making use of the ase library[1] to run optimisations with the NNP, with LBFGS being the optimiser of choice due to its efficiency in larger systems[4]. All optimisations were run at 0K neglecting thermal effects, first at a constant volume and then at constant pressure to allow for cell relaxation effects. A key benefit to using an NNP instead of DFT is that machine learning potentials typically scale linearly with system size[7] as they involve atom-wise contributions. Conversely, DFT based approaches will scale as N^3/N^4[8] which limits their utility in larger systems. A single basin-hopping run can run quite effectively on a local machine; however, to run many concurrent runs, it was necessary to utilise the HPC cluster myriad at UCL (University College London). In our approach, 10 structures were generated with completely random aluminium distributions, and for each aluminium atom, the corresponding sodium was also chosen based on a randomly selected location from the BVP approach. Using these random structures as the starting point, 10 independent basin-hopping runs were then submitted for 72 hours on GPUs for 10,000 maximum steps using a metropolis temperature of 450K and 1e-2 eV/Å as the optimiser tolerance, first at constant volume and then constant pressure. All structures across all runs within some energy window of the lowest-energy sampled structure were collected. Using the Python module `Pymatgen`[9], these were then filtered by symmetry such that only symmetry-unique configurations were collected. Con-

figurations were then re-optimised at a 1e-3 eV/Å tolerance at constant pressure. Provided sufficient sampling, this approach yields a representative sample of the lowest energy configurations.

5.1 Investigating relaxation effects

Figure S34 shows the relationship between initial and final (relaxed) structures for all possible configurations in a 2 Al/Na MOR 1×1×2 cell generated using the exhaustive approach. This shows a very poor degree of correlation, implying standard MC is insufficient for sampling realistic configurations.

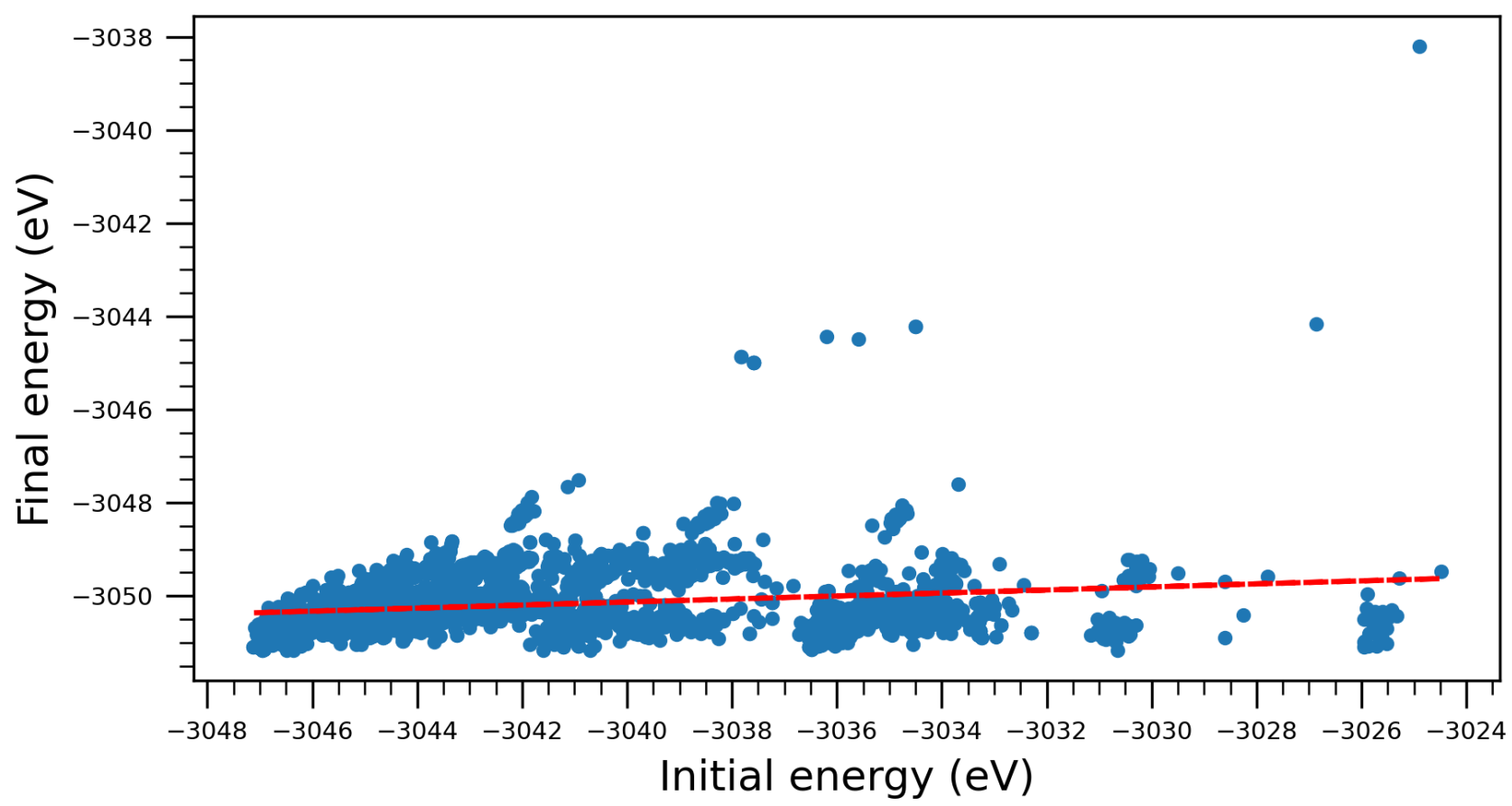

Fig. S34 Initial vs final relaxed energies in MOR (1x1x2). For the purposes of using more simplistic models for screening configurations, or classification approaches, ideally this would be a well correlated plot. It is clear from the line of best fit that the correlation is very weak where most initial configurations relax to within approximately 4eV of the global minimum but those initial configurations have a range of ≈ 22eV. Clearly the relaxation and structure generation needs greater understanding before the standard of correlation seen for silicates [10] is achievable.

For systems where certain cell lengths are significantly larger than others there is the possibility of introducing spatial relaxation artifacts as the cell may be able to relax in certain dimensions more than others, thus potentially biasing the distribution of aluminium. To investigate the degree of this bias 50 configurations in a 10 Al/Na CHA 1×2×1 cell were generated and relaxed, in each configuration the Al were placed randomly and the positions of their corresponding Na chosen based on randomly selected BVP positions. Once relaxed the cells were expanded to $2 \times 2 \times 2$ supercells such that each supercell was comprised of 4 periodic replicas of the original cell, each configuration was then re-relaxed. Figure S35 shows the absolute energies of each configuration at its relaxed energy in the $1 \times 2 \times 1$ cell compared to its expanded $2 \times 2 \times 2$ analogue. There is a high degree of correlation with the line of best fit having an R^2 of 0.999, suggesting the expanded cell does not permit significantly greater relaxation and thus using non-cubic cells does not significantly bias the global search.

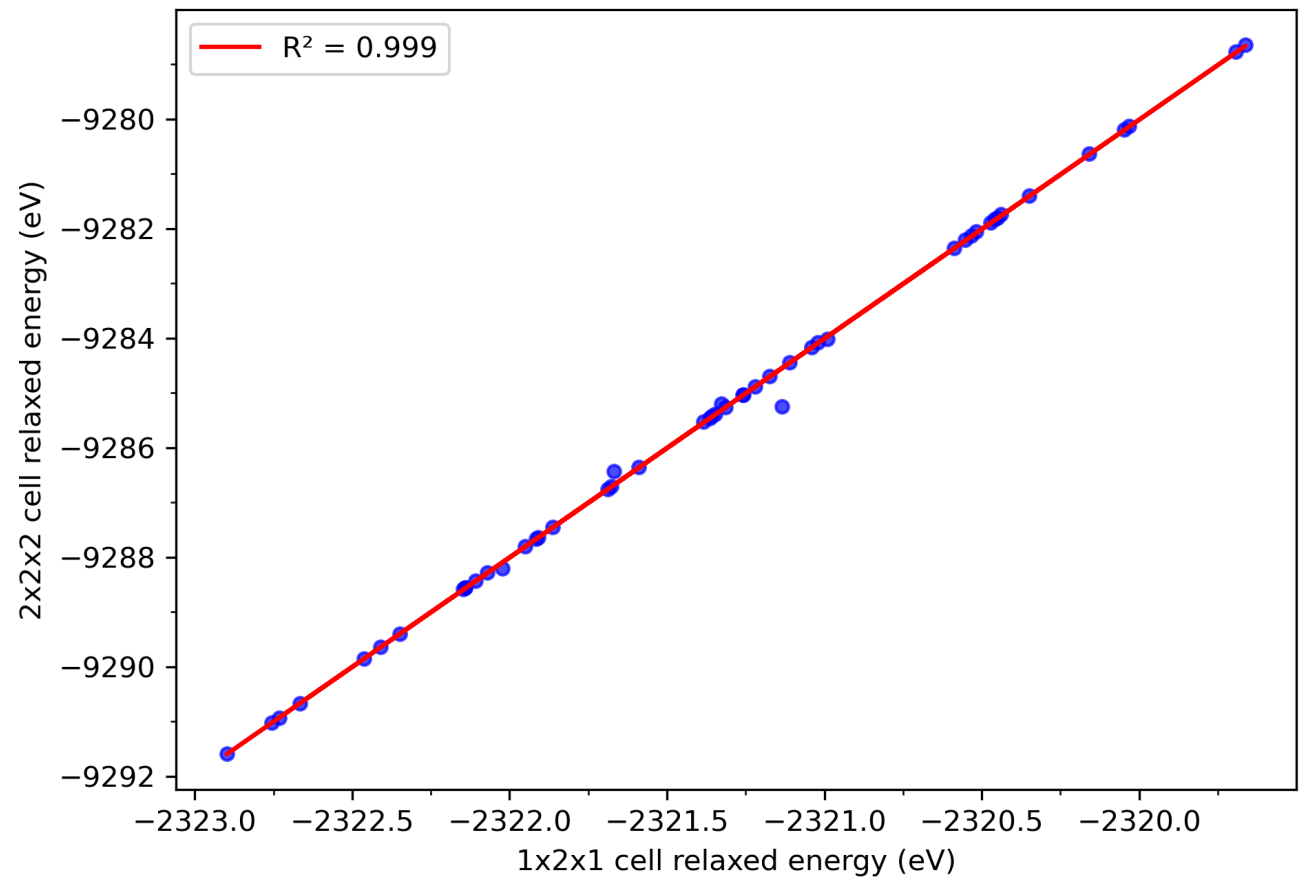

Fig. S35 Relaxed energy of 1x2x1 10 Al CHA configurations vs their 2x2x2 analogues which have been fully relaxed. Just one of the fifty structures shows that in the 1x2x1 cell, the structure is under-relaxed.

5.2 Move-class details

As mentioned in the main paper, one aspect of implementing the move-class that is surprisingly involved is identifying which sodium ion is associated with which aluminium. Intuitively one assumes that the simply using a distance based criterion to assess these pairings would be unambiguous. However, consider the scenario shown figure S36 where the left aluminium is paired with multiple sodium atoms. Either Na1 or Na2 could be closest to the old aluminium site, but ideally the algorithm should identify Na2, since Na1 is the sole compensator for the right aluminium site, so removing Na1 would result in a chemically implausible configuration

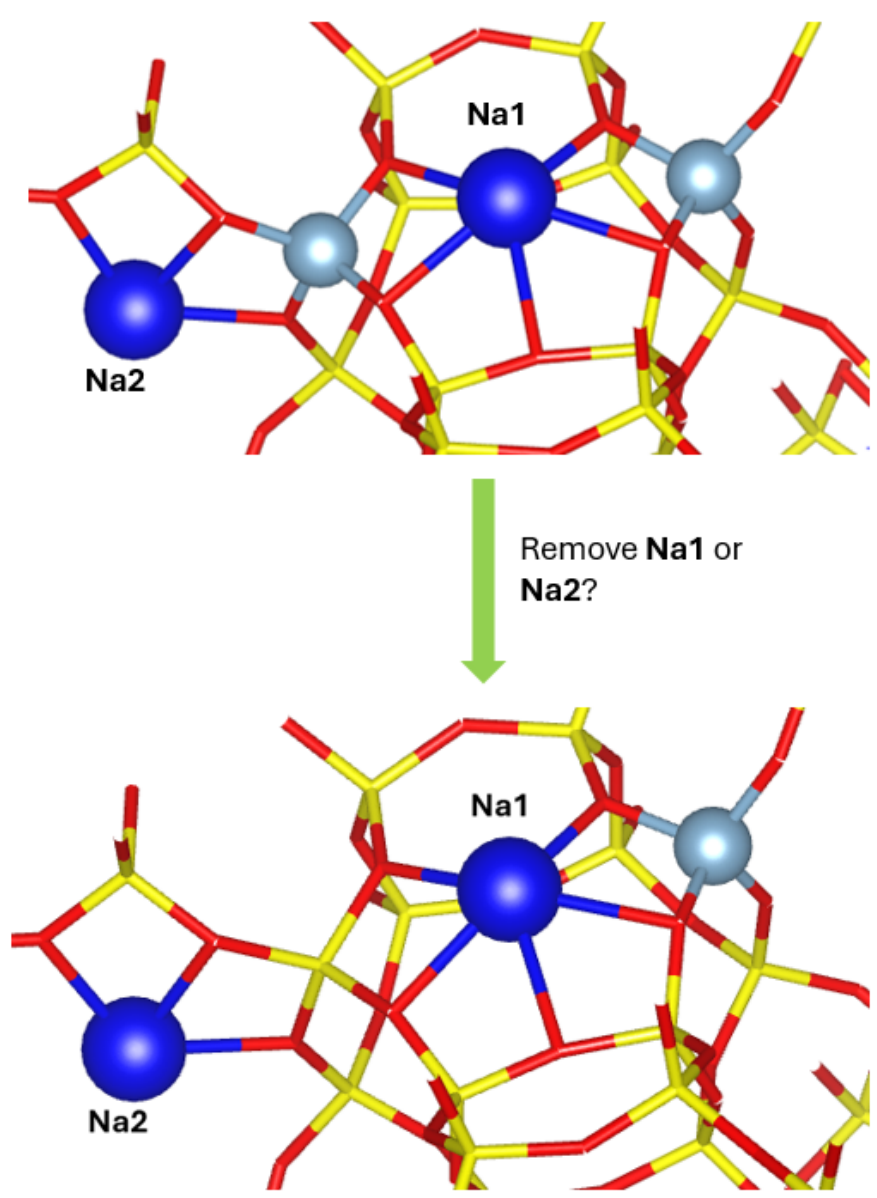

Fig. S36 Move-class scenario where the choice of which sodium to remove is not without ambiguity. In the upper figure there two aluminium atoms, but in the lower figure the aluminium on the left is replaced by silicon leaving a net charge associated with a supplementary sodium.

To address this scenario, the move-class utilises the "Hungarian algorithm"[11]implemented within the linear sum assignment module in scipy[12]. This establishes valid sodium/aluminium pairings by constructing a cost matrix where each element of the matrix corresponds to the distance between a given aluminium and sodium, accounting for periodic boundary conditions. If the distance is beyond

some cutoff threshold, typically 5-6.5 Å then the distance is assigned a value of infinity in the matrix. The algorithm then assigns pairs based on minimising the total cost, the sum of distances, yielding the best set of pairs. The next stages of the move-class can then be implemented by replacing a silicon with an aluminium and then inserting a sodium at a randomly selected position from the BVP approach at the new site. This approach may result in some asymmetries if the Al/Na pairing assignments change throughout the evolution of the algorithm, meaning accepted moves are not guaranteed to be directly reversible within a single subsequent BH step.

5.3 Improving efficiency

A few modifications to the algorithm were made to improve efficiency, these allowed for the algorithm to avoid high-energy regions of the energy landscape and also sample a greater number of configurations. During the initial structure generation, all configurations were typically constrained to be Lowenstein, meaning non-Lowenstein structure with Al-O-Al units were excluded, this is because it is generally accepted that NL structures are energetically unfavourable due to large electrostatic repulsion between aluminium atoms[13]. The random structure procedure also ignores structures where any pair of sodium atoms is less than 1Å apart since the associated electrostatic repulsion would result in a very high energy structure and lengthy relaxation times. Similar considerations were usually applied to the move class, any structures generated that were NL or of < 1 Å sodium-sodium distance would be automatically discarded without further optimisation.

6 Sampling

6.1 Assessing convergence

Although it is generally impossible to prove that basin-hopping is arriving at the correct global minimum, one can establish a degree of confidence in the sampling based on the tendency of independent runs to find similar structures. Consider the scenario where a large number of runs converge to the same low-energy structure, and no runs improve upon this threshold, then this would suggest that lower energy configurations either don't exist or are in basins that constitute a tiny percentage of the configuration space. The lowest energy configuration sampled across all runs will be referred to as the tentative global minimum in this section. To investigate the degree of convergence, we considered two main systems and multiple runs at differing temperatures for each. The systems of choice were 9 aluminium/sodium atoms in a 48 T-sites RHO unit cell, and 7 aluminium/sodium atoms in a 96 T-site $1 \times 1 \times 2$ MOR super-cell. For each system, 10 runs at 5 different temperatures (300K, 450K, 600K, 750K and 900K) were considered, yielding 50 independent runs per system.

In the MOR system it was found that 48/50 runs sampled the same lowest energy configuration, whilst the 2 remaining runs sampled a structure 0.04 eV lower in energy. However, upon inspection, it was evident that this lower energy configuration corresponded to an equivalent Al distribution to the slightly metastable but more commonly sampled configuration, and the sodium atoms were just in slightly different positions. In the RHO system, 9/50 runs identified the tentative global minimum, with 27/50 being within 0.1 eV of this energy and 46/50 within 0.2 eV. Overall, this represents a tendency for independent runs to converge to similar or identical configurations, implying sufficient representative sampling. It does not appear that any particular temperature used was significantly better or worse at identifying low-energy configurations, though this may be system-dependent.

6.2 Algorithm evolution

Below are two plots showing the evolution of a selection of 450K runs for the 7 Al MOR system Fig.S38 and the 9 Al RHO system Fig.S37, the plots only show the energies of accepted moves. It would appear that in MOR the energy landscape is much deeper than in RHO, with random structures being around 4 eV above the lowest energy structures the algorithm finds in the landscape, compared to RHO where this seems closer to 2 eV. In both systems there are early rapid improvements in energy with many downhill moves followed by a longer period of gradual refinement with less dramatic improvements in energy, though it would appear that in RHO uphill moves are more frequently accepted. This is possibly because in RHO configurations are separated by smaller energy differences, likely due to the higher symmetry, thus accepted moves are more probable, suggesting higher temperatures may be optimal in MOR.

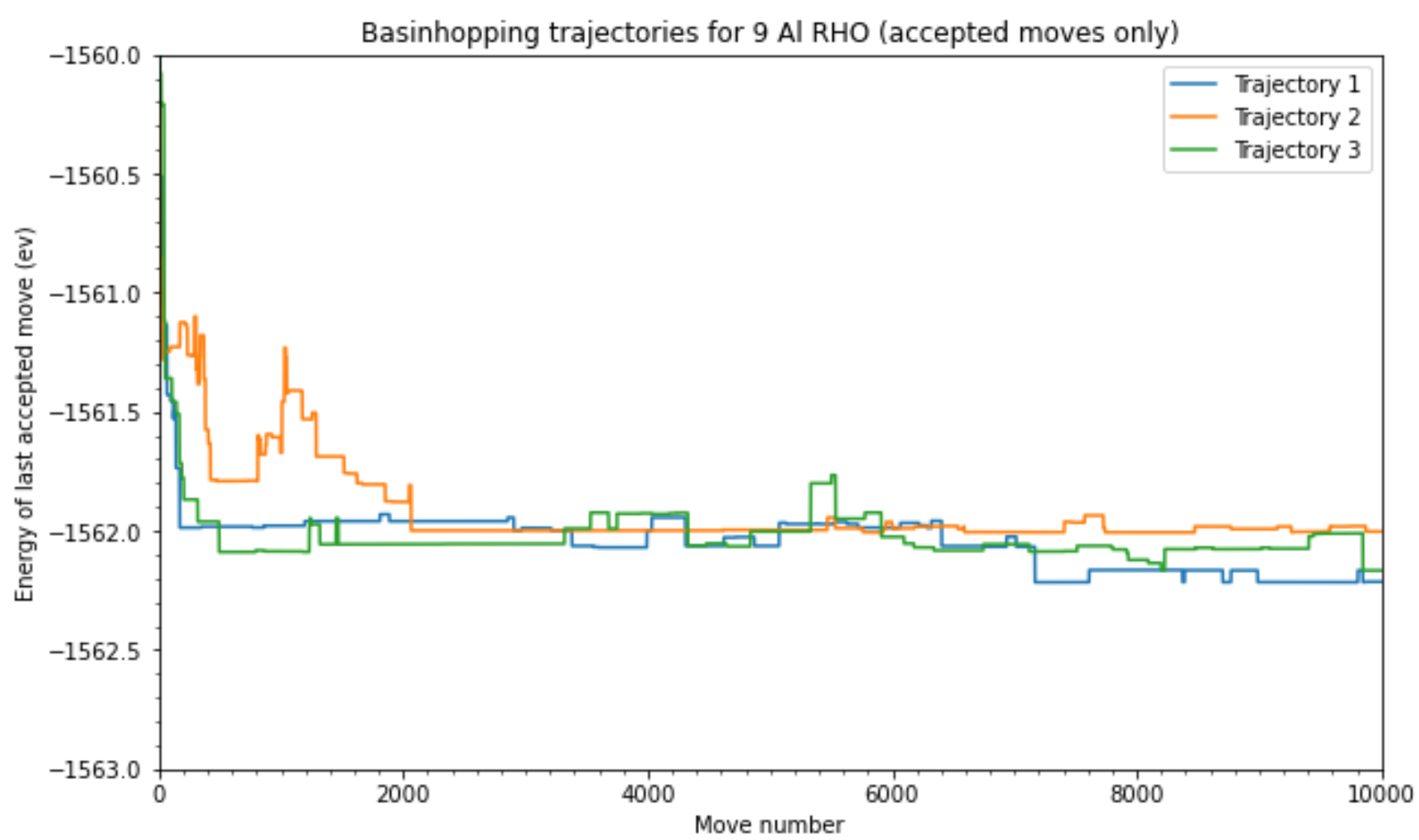

Fig. S37 Evolution of 3 different BH runs for 9 aluminium atoms in a $1 \times 1 \times 1$ RHO unit cell

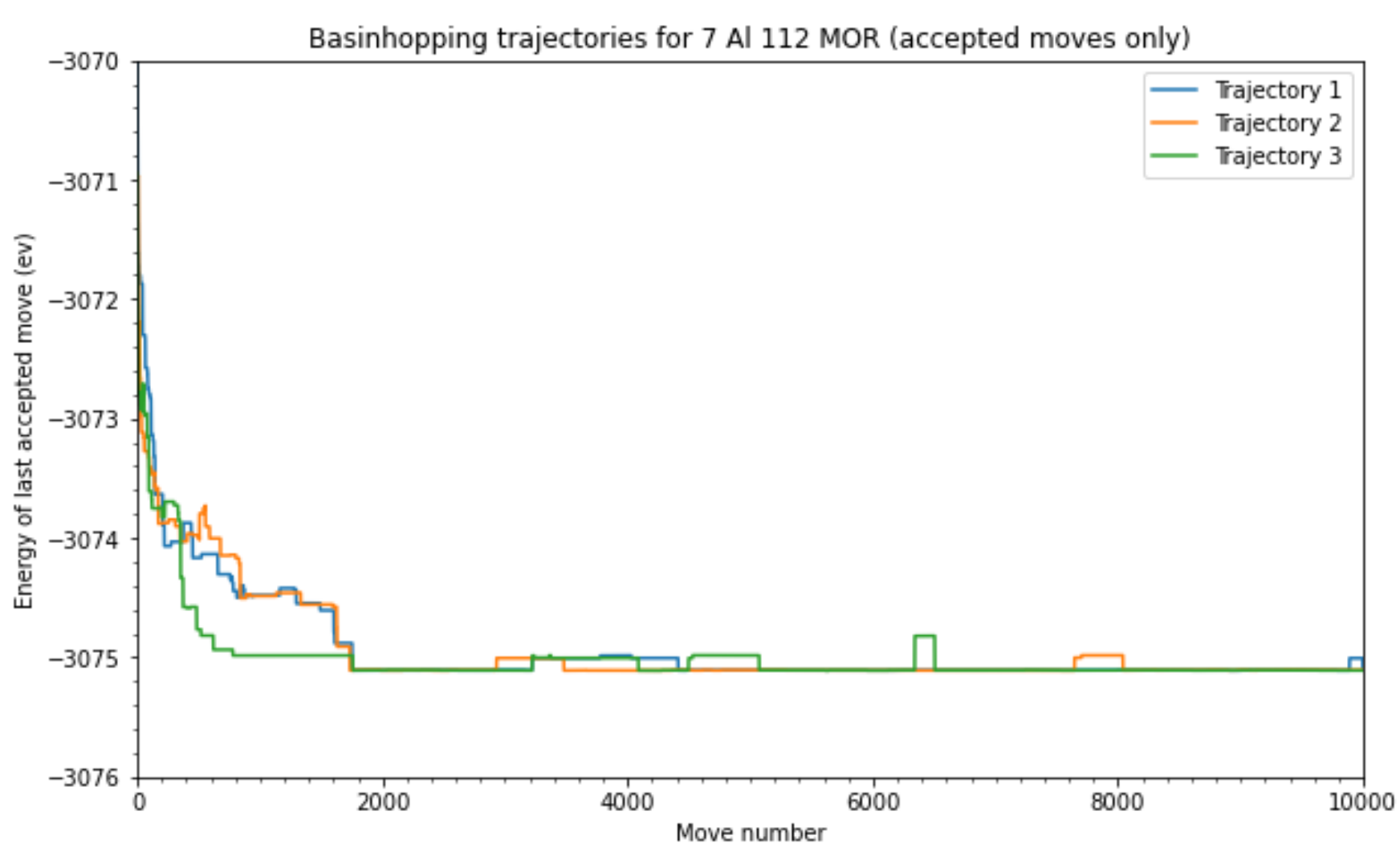

Fig. S38 Evolution of 3 different BH runs for 7 aluminium atoms in a $1 \times 1 \times 2$ MOR unit cell

6.3 Comparison with exhaustive search

This approach was initially developed by one of the authors, Dan Hewitt[14], and involved utilising the Python package `bsym`[15] to generate all symmetry unique configurations at a given number of aluminium atoms in a given system. This code would also generate all possible sodium permutations at a given number of aluminium atoms N_{Al}, which would scale as $4^{N_{Al}}$ or $6^{N_{Al}}$ depending on which sodium positioning algorithm was used. Because both the number of aluminium distributions and the number of sodium permutations would scale exponentially with the aluminium content, this meant that optimising all configurations became intractable beyond a few substitutions. For N_T T-sites and N_{Al} aluminium atoms the number of possible aluminium distributions scales as the binomial coefficient $\Omega(N_T, N_{Al})$:

$$\Omega(N_T, N_{Al}) = \binom{N_T}{N_{Al}} = \frac{N_T!}{N_{Al}!(N_T - N_{Al})!} \tag{4}$$

However, given that bsym filters out symmetry redundant configurations the true number of distributions is lower than the binomial coefficient, though it will still scale exponentially with system size. Thus, the total number of configurations N_{total} when using the vector projection algorithm will be proportional as:

$$N_{total} \propto \binom{N_T}{N_{\mathrm{Al}}} \cdot 4^{N_{\mathrm{Al}}} \tag{5}$$

And using the edges algorithm will be:

$$N_{total} \propto \binom{N_T}{N_{\mathrm{Al}}} \cdot 6^{N_{\mathrm{Al}}} \tag{6}$$

Where in both cases, different sodium positioning algorithms have been used. The degree to which symmetry reduces the number of configuration is dependent on the particular topology, in a very symmetric system like chabazite the number of configurations is greatly reduced by this constraint, whereas in a lower symmetry zeolite like MFI, a lower percentage of configurations are filtered. As the total number of T-sites N_T increases, the exponential scaling becomes progressively worse, making brute-force evaluation often intractable beyond a very small number of aluminium atoms. For instance, in the MFI framework, two aluminum and two sodium ions yield $\approx$ 22,000 possible structures; with three aluminium and three sodium ions, this grows to $\approx$ 3,000,000, and with four aluminium and sodium ions, it exceeds $\approx$ 700,000,000 configurations for a single unit cell.

Given that this brute force approach can exhaustively calculate all feasible configurations, it serves as a natural benchmark to compare with basin-hopping approach. In $1 \times 1 \times 1$ chabazite it was found that for 3 aluminium atoms and 3 sodium atoms 9 of 10 independent basin-hopping runs identified at or near global minimum configurations.

6.4 Algorithm modification to assess sodium bias

Since the standard basin-hopping approach constrains the possible initial guesses for the sodium positions, this may bias the algorithm towards regions of the landscape that are not necessarily lowest in energy. For example in RHO for one aluminium, it was found by combining molecular dynamics with quenching that the BVP approach identifies an initial position which upon relaxation yields the global minimum of the system. However, as more aluminium and sodium is added, this may no longer be the preferred position since the additional degrees of freedom and interactions between charged species may result in a more complex energy surface with new minima. Thus, if the positions of the sodium

are constrained to positions that aren't always globally optimal this may result in an error which propagates as more aluminium is added to the system. To test this a "Basin-hopping with sodium perturbation" approach was introduced, this takes the original approach and modifies the moveclass such that after a sodium is placed on a selected "edge" it is then randomly displaced. The random displacement is achieved by generating a unit vector in a random direction and scaling it by a random number between 0 and 2, the sodium is then perturbed along this vector, resulting in a displacement of the sodium position of 0 to 2 angstroms in a random direction (avoiding close contacts). The structure is then optimised and accepted/rejected as per the usual criterion. It was found that when applied to a 9 aluminium 48 T-site RHO system this approach did not yield any structures lower in energy than the lowest energy structure sampled within the standard approach, suggesting that our assumption of constraining the sodium positions is valid.

6.5 Stepwise configuration approach

Another algorithm developed was a stepwise configuration algorithm, which would bias the sampling towards lower energy regions of the landscape. This algorithm would essentially use the lowest-energy structure for a given number of aluminium atoms as a seed for the next substitution, keeping all aluminium and sodium atoms from the previous loading fixed and generating configurations by systematically replacing all unoccupied T-sites with an aluminium, considering all possible sodium positions at each site. These configurations were then optimised first at constant volume and then at constant pressure, with the lowest energy structure being taken as the GM for that Al loading, which would then be used as a seed for the next iteration. This approach was very computationally efficient as it would typically only involve optimising a few hundred configurations at each Al loading whilst ensuring a bias toward low-energy configurations. However, this bias may steer the sampling towards regions of the configuration space that, although relatively low in energy, may not be within the lowest energy basin of the system. Figure S7 shows a comparison of the stepwise approach with basin-hopping in a $1 \times 2 \times 1$ CHA system, figure S8 shows a comparison in $1 \times 1 \times 1$ RHO, in both tables the energies correspond to the most stable structure found within each approach.

Table S7 121 CHA BH comparison with Stepwise algorithm

N_{Al}	BH (eV)	Stepwise (eV)	ΔE (eV)
2	-2288.76	-2288.76	0.00
3	-2293.18	-2293.18	0.00
4	-2297.92	-2297.90	0.02
5	-2302.32	-2302.31	0.01
6	-2307.06	-2307.05	0.01
7	-2311.40	-2311.40	0.00
8	-2316.09	-2315.97	0.12

Table S8 111 RHO comparison with Stepwise algorithm

N_{Al}	BH (eV)	Stepwise (eV)	ΔE (eV)
3	-1533.45	-1533.27	0.18
4	-1538.31	-1537.89	0.38
5	-1543.08	-1542.33	0.75
6	-1547.86	-1546.84	1.02
7	-1552.68	-1551.35	1.33
8	-1557.49	-1556.01	1.48

7 Comparing Topologies

We now seek to make more general comments, looking across the three topologies of CHA, MOR and RHO. Comparison of average number of Si between Al for each topology and the thermodynamic plot.

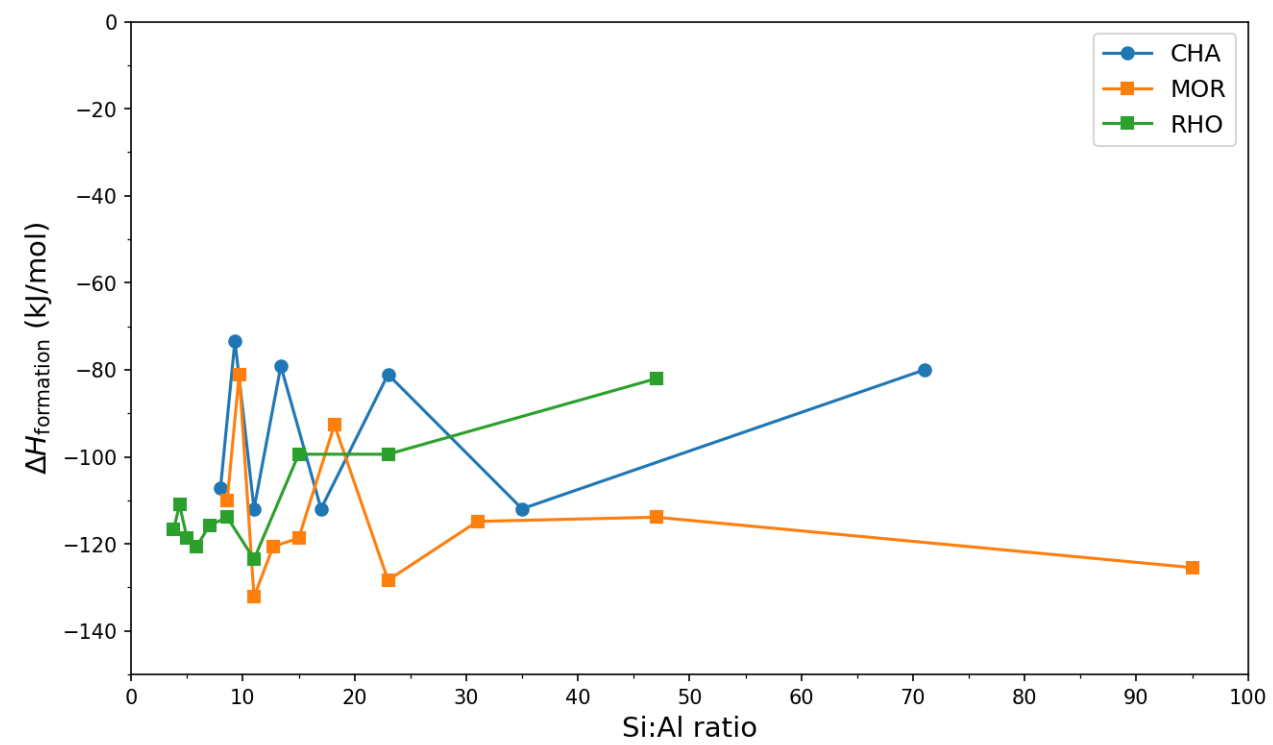

Fig. S39 The substitution or incorporation energy of Al and Na in place of Si as function of Si:Al ratio for CHA, MOR and RHO.

In figure S39 we can see how the energy of incorporation varies across topology. The plots are very non-linear but this is an artefact, in part, of using a relatively small simulation cell. It is expected that in larger cells, which capture Si:Al ratio more continuously, the line of best fit might be easier to identify. Naively, one expects that it might be more favourable to incorporate the larger Al^{3+} ion into lower density structures, as these structures are expected to contain more distorted environments, as this is a well established link between density and stability[16]. Indeed, deformation of O-T-O and T-O-T have been shown to be key descriptors[17 18 19], though more sophisticated descriptor sets are necessary to capture structural relaxation effects well[10]. However, it would appear that the energetics of aluminium incorporation are largely dictated by electrostatics, rather than alleviating strain, where the coordination environment of the sodium is found to be particularly important. Initial analysis of chabazite using `RandomForest` and `LASSO` regression algorithms using a range of descriptors, identifies the most important factor is the sodium coordination with oxygen atoms that are bonded to the nearest aluminum, which we call the "NaOAl" descriptor, rather than a general sodium-oxygen coordination metric. This is presumably due to the greater electronegativity of Al-O bonds compared with Si-O, though more work on this is needed and it is unclear how well this trend generalises across topologies. Details of how the "NaOAl" descriptor was calculated is described in section 10.

Figure S39 also shows that at low to medium silica ratios, the formation energies are rather similar. In fact, the average formation energies for RHO and MOR are -110.2 and -113.6 kJ/mol. According to Navrotsky and Tian[20] a generic fitted model averaged over sodiated zeolites (most with higher aluminium content than has been explored here) with calorimetric data gives an estimate of around -135 kJ/mol, again in fair quantitative agreement.

8 Validation of chosen energy window

In this research we use an arbitrary 30 kJ/mol window for the Boltzmann weighted plots, to validate that our conclusions aren't strongly dependent on this window our results for T-site occupations in MOR were compared to using a 20 kJ/mol window. As is shown in Tables S9 and S10 the T-site occupations are effectively unchanged by using this smaller window with only minor deviations in T-site occupations.

Table S9 Boltzmann weighted T-site occupations in $1\times1\times2$ MOR supercell at various Al loadings using 30 kJ/mol energy window

Aluminium number	T1	T2	T3	T4
1	0.415	0.002	0.580	0.003
2	0.558	0.001	1.440	0.001
3	0.004	0.000	2.996	0.001
4	0.000	0.000	4.000	0.000
5	0.180	0.001	4.817	0.001
6	0.034	0.000	5.966	0.000
7	0.001	0.000	6.999	0.000
8	0.000	0.000	8.000	0.000
9	0.996	0.002	7.989	0.013
10	2.000	0.000	8.000	0.000
11	3.003	0.004	7.989	0.003
12	4.000	0.000	8.000	0.000

Table S10 Boltzmann weighted T-site occupations in $1\times1\times2$ MOR supercell at various Al loadings using 20 kJ/mol energy window

Aluminium number	T1	T2	T3	T4
1	0.416	0.000	0.584	0.000
2	0.553	0.000	1.447	0.000
3	0.000	0.000	3.000	0.000
4	0.000	0.000	4.000	0.000
5	0.178	0.000	4.822	0.000
6	0.033	0.000	5.967	0.000
7	0.000	0.000	7.000	0.000
8	0.000	0.000	8.000	0.000
9	0.999	0.000	7.993	0.008
10	2.000	0.000	8.000	0.000
11	3.008	0.000	7.992	0.000
12	4.000	0.000	8.000	0.000

9 Additional Structure Figures

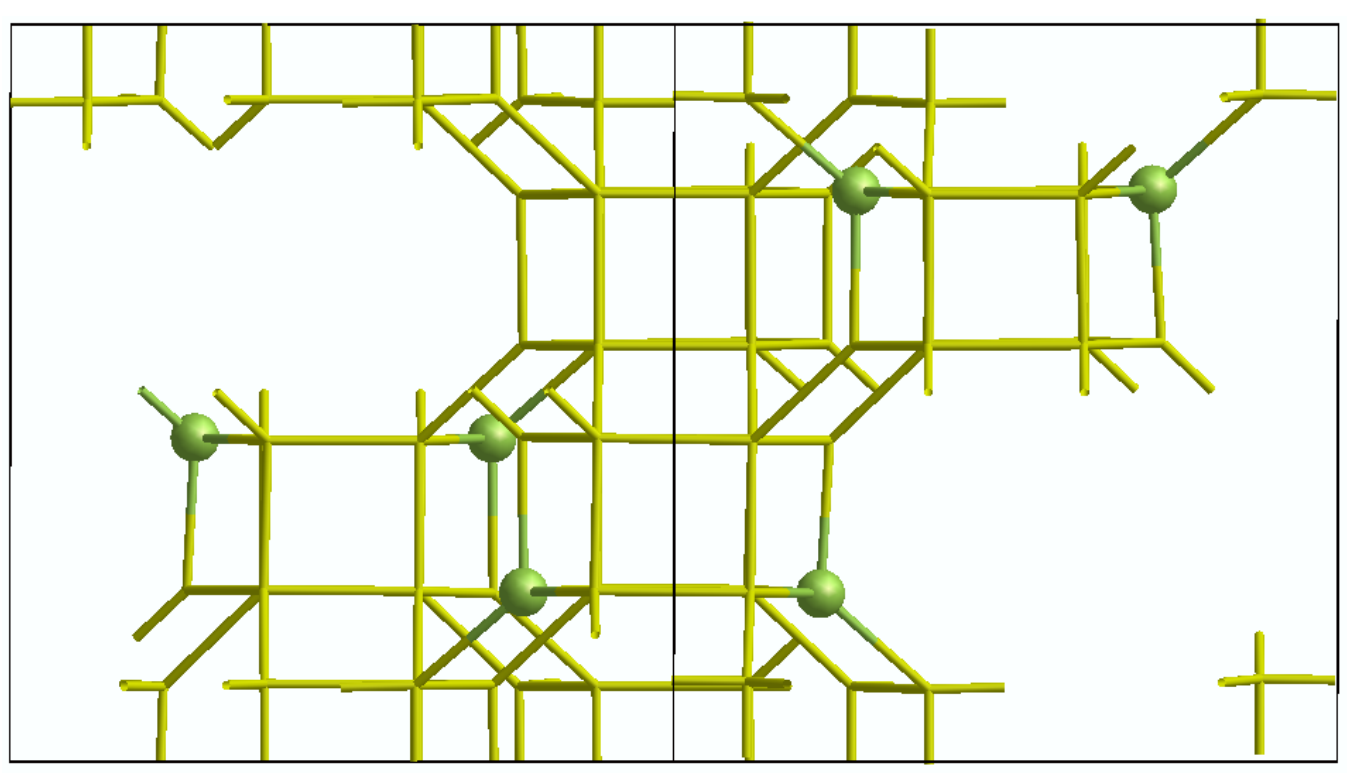

Fig. S40 $1\times2\times1$ CHA global minimum for 6 aluminium atoms with oxygen and sodium atoms omitted for clarity, silicon sites in yellow and aluminium in light green

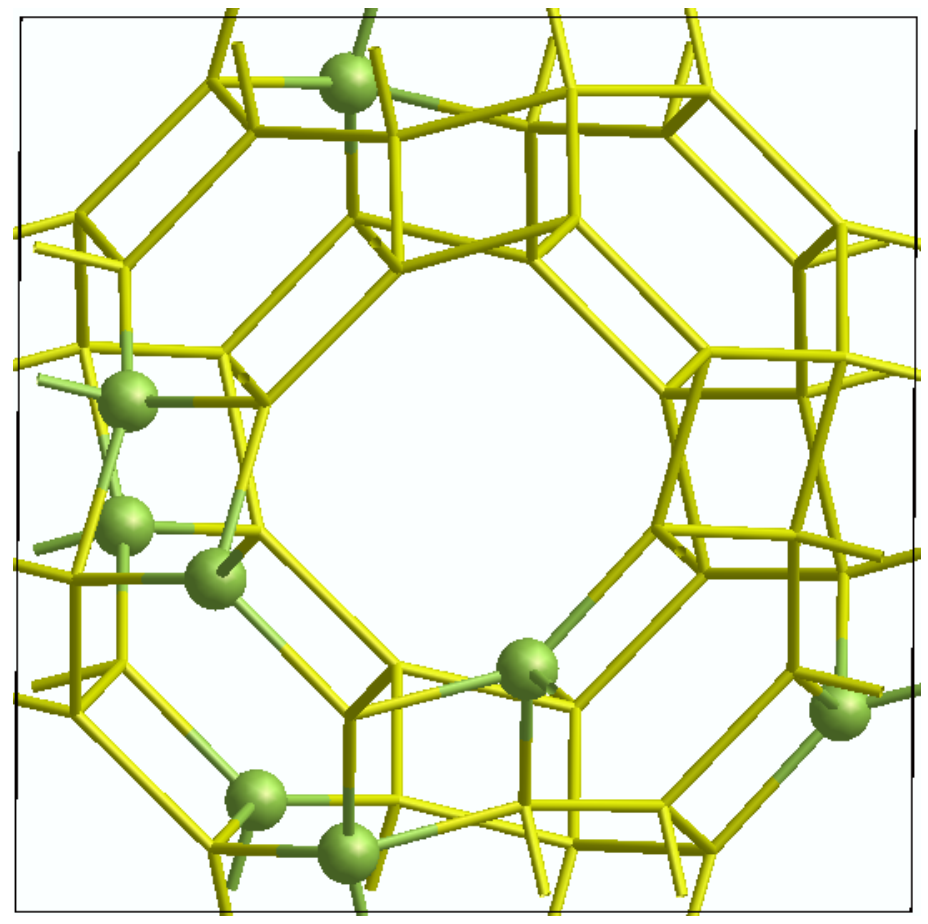

Fig. S41 $1 \times 1 \times 1$ RHO global minimum with 8 aluminium atoms, sodium and oxygen omitted. Viewed along the [100] axis.

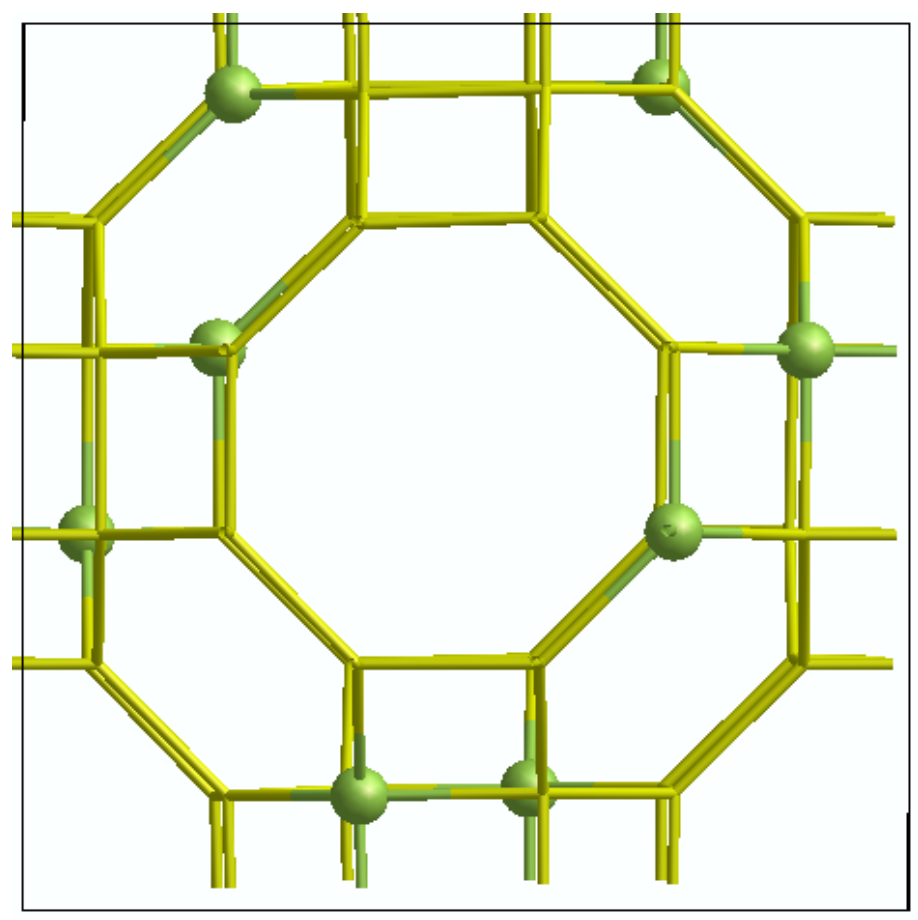

Fig. S42 $1 \times 1 \times 1$ RHO global minimum according to the stepwise approach with 8 aluminium atoms, sodium and oxygen omitted. Viewed along the [100] axis.

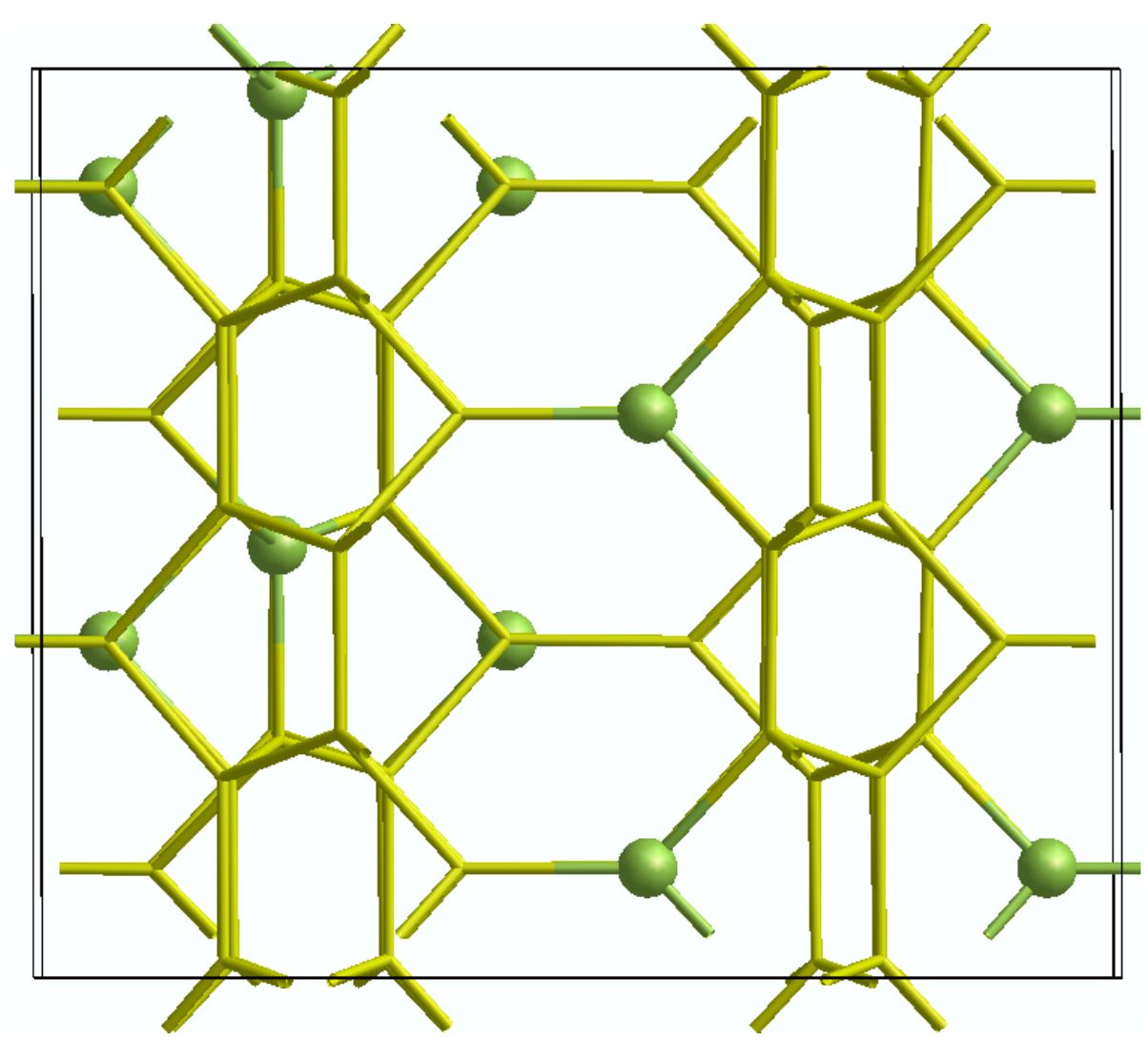

Fig. S43 $1 \times 1 \times 2$ MOR global minimum with 10 aluminium atoms, sodium and oxygen omitted. Viewed along the [010] axis.

10 Regression

In order to gauge the which structural features are driving the energetics of the landscape Lasso regression and Random forest regression algorithms were implemented in Scikit-learn. Features ranged from descriptors related to framework strain such as mean/variance of TOT angles to electrostatic features such as the degree of sodium coordination.

10.1 Coordination Descriptors

To measure the degree of sodium coordination two separate descriptors were formulated, the first relates to the total sodium oxygen coordination "NaO" and the second, "NaOAl", explicitly considers the coordination to oxygens bonded with aluminum sites. Each descriptor is essentially a sum over all sodium atoms in the system, each weighted by the degree of coordination with framework oxygens. The coordination of each sodium is defined by the sum of all oxygen coordinations within a 3Å radius of said sodium, where each coordination is taken as 1/r where r is the sodium-oxygen distance.

$$NaO = \sum_{i=1} Na_i O \tag{7}$$

$$Na_i O = \sum_{j=1} 1/r_j \tag{8}$$

$$NaOAl = \sum_{i=1} Na_i OAl \tag{9}$$

$$Na_i OAl = \sum_{j=1} 1/r_j \tag{10}$$

References

1 A. Hjorth Larsen, J. Jørgen Mortensen, J. Blomqvist, I. E. Castelli, R. Christensen, M. Dułak, J. Friis, M. N. Groves, B. Hammer, C. Hargus, E. D. Hermes, P. C. Jennings, P. Bjerre Jensen, J. Kermode, J. R. Kitchin, E. Leonhard Kolsbjerg, J. Kubal, K. Kaasbjerg, S. Lysgaard, J. Bergmann Maronsson, T. Maxson, T. Olsen, L. Pastewka, A. Peterson, C. Rostgaard, J. Schiøtz, O. Schütt, M. Strange, K. S. Thygesen, T. Vegge, L. Vilhelmsen, M. Walter, Z. Zeng and K. W. Jacobsen, *Journal of Physics: Condensed Matter*, 2017, **29**, 273002.

2 T. D. Kühne, M. Iannuzzi, M. Del Ben, V. V. Rybkin, P. Seewald, F. Stein, T. Laino, R. Z. Khaliullin, O. Schütt, F. Schiffmann, D. Golze, J. Wilhelm, S. Chulkov, M. H. Bani-Hashemian, V. Weber, U. Borštnik, M. Taillefumier, A. S. Jakobovits, A. Lazzaro, H. Pabst, T. Müller, R. Schade, M. Guidon, S. Andermatt, N. Holmberg, G. K. Schenter, A. Hehn, A. Bussy, F. Belleflamme, G. Tabacchi, A. Glöß, M. Lass, I. Bethune, C. J. Mundy, C. Plessl, M. Watkins, J. VandeVondele, M. Krack and J. Hutter, *The Journal of Chemical Physics*, 2020, **152**, 194103.

3 J. VandeVondele, M. Krack, F. Mohamed, M. Parrinello, T. Chassaing and J. Hutter, *Computer Physics Communications*, 2005, **167**, 103–128.

4 D. C. Liu and J. Nocedal, *Mathematical programming*, 1989, **45**, 503–528.

5 S. Grimme, J. Antony, S. Ehrlich and H. Krieg, *The Journal of Chemical Physics*, 2010, **132**, 154104.

6 M. Guidon, J. Hutter and J. VandeVondele, *Journal of chemical theory and computation*, 2010, **6**, 2348–2364.

7 J. Behler, *The Journal of Chemical Physics*, 2016, **145**, 170901.

8 N. Hine, P. Haynes, A. Mostofi, C.-K. Skylaris and M. Payne, *Computer Physics Communications*,

2009, **180**, 1041–1053.
9 S. P. Ong, W. D. Richards, A. Jain, G. Hautier, M. Kocher, S. Cholia, D. Gunter, V. L. Chevrier, K. A. Persson and G. Ceder, *Computational Materials Science*, 2013, **68**, 314–319.
10 S. R. Balestra, N. Rodríguez-Sánchez, D. Mena-Torres and A. R. Ruiz-Salvador, *Crystal Growth & Design*, 2024, **24**, 938–946.
11 H. W. Kuhn, *Naval Research Logistics Quarterly*, 1955, **2**, 83–97.
12 P. Virtanen, R. Gommers, T. E. Oliphant, M. Haberland, T. Reddy, D. Cournapeau, E. Burovski, P. Peterson, W. Weckesser, J. Bright *et al.*, *Nature methods*, 2020, **17**, 261–272.
13 C.-S. Yang, J. M. Mora-Fonz and C. R. A. Catlow, *The Journal of Physical Chemistry C*, 2011, **115**, 24102–24114.
14 D. Hewitt, *PhD Thesis; Rapid Screening of Zeolites using Computational and Machine Learning Techniques, UCL*, `https://discovery.ucl.ac.uk/id/eprint/10197750/`, 2024.
15 B. J. Morgan, *Journal of Open Source Software*, 2017, **2**, 370.
16 N. J. Henson, A. K. Cheetham and J. D. Gale, *Chem. Mater.*, 1994, **6**, 1647–1650.
17 G. A. Tribello, B. Slater, M. A. Zwijnenburg and R. G. Bell, *Phys. Chem. Chem. Phys.*, 2010, **12**, 8597–8606.
18 M. A. Zwijnenburg, S. T. Bromley, M. D. Foster, R. G. Bell, O. Delgado-Friedrichs, J. C. Jansen and T. Maschmeyer, *Chemistry of Materials*, 2004, **16**, 3809–3820.
19 Y. Li, J. Yu and R. Xu, *Angewandte Chemie International Edition*, 2013, **52**, 1673–1677.
20 A. Navrotsky and Z.-R. Tian, *Chemistry – A European Journal*, 2001, **7**, 769–774.
